# Effectiveness of Telehealth Care Interventions for Women’s Mental Health During and After the COVID-19 Pandemic: Systematic Review and Meta-Analysis

**DOI:** 10.2196/85494

**Published:** 2026-09-21

**Authors:** Serena Kao, Athina Marina Metaxa, Ranin Soliman

**Affiliations:** 1 Department of Nutritional Science, School of Life Course and Population Sciences, King’s College London, Franklin-Wilkins Building, 150 Stamford Street, London, SE1 9NH, United Kingdom, 44 (0)20 7836 5454, 44 (0)20 7123 484; 2 Center for Evidence-Based Medicine, Nuffield Department of Primary Care Health Sciences, University of Oxford, Oxford, United Kingdom; 3 School of Life and Medical Sciences, University of Hertfordshire Hosted by Global Academic Foundation, Cairo, Egypt; 4 Evidence and Data to Policy Unit, WHO Regional Office for the Eastern Mediterranean (WHO EMRO), World Health Organization, Cairo, Egypt

**Keywords:** depression, anxiety, stress, telemental health, telemedicine, mobile health, mental health, digital health, telehealth, eHealth, women’s health, review, meta-analysis, PRISMA, evidence-based health care, systematic review

## Abstract

**Background:**

The COVID-19 pandemic has increased telemental health interventions. However, evidence of effectiveness in improving women’s mental health outcomes, acceptability, and user satisfaction remains fragmented.

**Objective:**

This study examined the effectiveness of telehealth interventions in alleviating depression, anxiety, and stress in women during and after the pandemic and descriptively summarized the acceptability of and satisfaction with these interventions.

**Methods:**

This systematic review and meta-analysis compared telemental health interventions with control conditions (waitlists or standard care) for women experiencing anxiety, depression, or stress. Databases (PubMed/MEDLINE, Embase, Web of Science, PsycINFO, and Cochrane Central Register of Controlled Trials) were searched through March 19, 2026. Eligible studies evaluated interventions in women aged 18 to 64 years (≥80% female participants and female-specific outcomes) and included randomized controlled trials (RCTs), nonrandomized trials, and observational studies with ≥5 participants using outcome measures. Random-effects meta-analyses were conducted for depression, anxiety, and stress using RCT data. Risk of bias was assessed using Cochrane tools and certainty of evidence using Grading of Recommendations, Assessment, Development and Evaluation.

**Results:**

Fifty-seven studies (N=13,457) from 24 countries, reported the effectiveness, acceptability, and satisfaction with telehealth interventions. Meta-analysis of 23 studies (n=3269) demonstrated significant benefits of telehealth interventions for depression, anxiety, and stress. Depression showed the greatest improvements, with a moderate reduction in symptoms (Hedges *g*=−0.56, 95% CI −0.86 to −0.26; *P*<.001), followed by anxiety (small to moderate reduction; Hedges *g*=−0.45, 95% CI −0.62 to −0.28; *P*<.001). Stress showed a smaller effect (Hedges *g*=−0.25, 95% CI −0.47 to −0.04; *P*=.02), indicating modest benefits. Heterogeneity was high, with 95% prediction intervals of −1.97 to 0.84, −1.11 to 0.21, and −0.98 to 0.40 for depression, anxiety, and stress, respectively. Subgroup analyses favored telehealth over waitlist and standard care for depression (waitlist: Hedges *g*=−0.60, 95% CI −0.86 to −0.33; standard care: Hedges *g*=−0.57, 95% CI −1.11 to −0.02; both *P*<.001) and anxiety (waitlist: Hedges *g*=−0.47, 95% CI −0.68 to −0.25; standard care: Hedges *g*=−0.52, 95% CI −0.80 to −0.25; both *P*<.001). Insufficient studies were available to evaluate stress in subgroup analyses. Treatment effects remained significant in female-only and predominantly female samples. Evidence certainty was downgraded due to heterogeneity, risk of bias, and methodological limitations.

**Conclusions:**

This is the first systematic review and meta-analysis focused on telemental health interventions for women. Telehealth was associated with moderate, small to moderate, and modest reductions in depression, anxiety, and stress, respectively, with benefits across female-only and predominantly female samples. Moderate to high heterogeneity in average effects (CIs), wide distributions of effects across settings (prediction intervals), and risk of bias resulted in moderate certainty of evidence. Overall, telehealth is a scalable approach for improving access to mental health care for women, although high-quality research is needed to strengthen the evidence base.

## Introduction

### Background

The COVID-19 pandemic has profoundly disrupted lives, leaving a lasting impact on mental health and well-being. Globally, the pandemic triggered an unparalleled mental health crisis, driven by a fear of the virus, prolonged lockdowns, and pervasive social isolation [1]. In the aftermath of the COVID-19 pandemic, ongoing challenges such as uncertainty, rapid societal changes, shared grief, and the lingering psychological impact of the pandemic have deeply shaped mental health in complex and evolving ways [2], with a particularly sharp increase in the prevalence of depression, anxiety, and stress worldwide [3-6]. This situation has revealed significant gaps in accessing mental health care, particularly for those who are most vulnerable.

According to data from the World Health Organization scientific brief published in 2022, the global prevalence of anxiety and depression rose by 25% during the first year of the pandemic [7]. While women have historically been at greater risk than men for developing stress, anxiety, and depression, whether as standalone conditions or co-occurring disorders [5,8-12], recent studies have indicated that the prevalence of these mental health conditions has further increased among women during the COVID-19 pandemic [13-15]. Recent reviews have highlighted challenges related to mental health of women and underscored the importance of public health systems addressing the specific needs of female populations [16]; the stress of household and familial responsibilities, compounded by isolation measures and the fear of illness that were prominent during the COVID-19 pandemic, are highlighted as key factors that increased the emotional burden faced by women [17-26]. Moreover, restricted access to maternal health services, including prenatal, delivery, and postpartum care, further compromised the well-being of many women [16]. In this context, technologies enabling remote support and consultations by mental health professionals through internet platforms, mobile apps, and telephone or video calls [27] can provide essential assistance to women facing mental health difficulties. To date, there are no systematic reviews or meta-analyses specifically addressing gender-focused care in the context of telemental health for women.

The COVID-19 pandemic has also significantly disrupted access to traditional in-person mental health services due to the restrictions placed across countries during quarantine and lockdown, which greatly increased the reliance on telehealth options to provide health care services remotely [28-30]. While the field of telehealth interventions (THIs) was slowly growing before 2020, it expanded rapidly following the relaxation of regulatory barriers in response to the pandemic, and it has become a permanent component of health care delivery ever since [31-36]. THIs offer significant benefits, such as increased accessibility and flexibility, reduced dependency on direct therapist involvement, time saved from traveling, and the ability to address health crises effectively [37,38]. While THIs have shown promising results for the treatment of depression and anxiety in the general population [39-43], concerns about the use of THIs remain. Specifically, knowledge gaps persist around optimal patient selection strategies; data security; and the level of acceptability, safety, and cost-effectiveness of such interventions [37,44,45]. Understanding patient satisfaction with THIs is also crucial, given its direct influence on treatment adherence and effectiveness [38].

### Study Objectives

Despite evidence of a severe negative impact of the recent COVID-19 pandemic on women’s mental health [13-15,46], published data on THIs for women are scarce. While the shift toward telehealth care presents an opportunity to address disparities in care, the effectiveness and acceptability of and patient satisfaction with THIs require further evaluation. This systematic review and meta-analysis aimed to investigate the effectiveness of THIs for depression, anxiety, and stress among women during and after the COVID-19 pandemic. A secondary aim was to explore the acceptability of and the reported patient satisfaction with these interventions among women participants in the identified studies.

## Methods

### Ethical Considerations

This systematic review and meta-analysis did not involve the collection of primary data or recruitment of participants, and all data analyzed were obtained from previously published studies that had received appropriate ethical approval. As no individual patient data were accessed, and only aggregated results were used, requirements for formal ethical approval have been waived by the Center of Evidence-Based Medicine and the ethics committee of the University of Oxford Nuffield Department of Medicine.

### Reporting Guidelines

This study followed and adhered to the Preferred Reporting Items for Systematic Reviews and Meta-analysis (PRISMA) 2020 guidelines [47], PRISMA-S (PRISMA Literature Search extension) guidelines [48], and guidance by the Cochrane Handbook for systematic reviews of interventions [49]. The protocol that was followed was registered in PROSPERO international database for systematic reviews and meta-analysis (CRD42022360516).

### Search Strategy

The following databases of published and gray literature were searched from inception to March 19, 2026: MEDLINE (OvidSP), Embase (OvidSP), CINAHL (EBSCOHost), Science Citation Index, Social Science Citation Index and Conference Proceedings Citation Index (Web of Science), Scopus (Elsevier), Sage Journals, PsycINFO (OvidSP), Cochrane Central Register of Controlled Trials (Cochrane Library, Wiley), EconStor, JSTOR, Google Scholar, and CoCites. Two trial registries, Clinicaltrials.gov COVID-19 collection [50] and WHO ICTRP [51], were also searched to January 25, 2024. The search included title, abstract, author keywords, and subject headings for our key concepts of COVID-19, depression or anxiety, and telehealth. The National Institute for Health and Care Excellence (NICE) MEDLINE and Embase (Ovid) health app search filters were adapted to include additional keywords for telehealth [52]. Methodological search filters were applied to restrict the search to trials and nonrandomized studies. No date or language limits were applied. Records were imported into Covidence, where duplicates were identified and removed; additional duplicates were then identified manually. A list of relevant citations was generated from these databases. The title and abstract of these citations were screened to identify all potentially relevant articles, which were eligible for full-text evaluation. A detailed description of each search strategy is given in Multimedia Appendix 1.

### Inclusion and Exclusion Criteria

The inclusion and exclusion criteria were developed using the PICOS (population, intervention, comparison, outcome, and study design) framework to guide the selection of relevant studies and ensure a thorough and comprehensive review (Table 1) [53]. This systematic review focused on interventional studies (randomized controlled trials [RCTs], observational studies, cohort studies, and case-control studies) conducted since the start of the COVID-19 pandemic (2020). Studies were eligible if they included at least 1 intervention delivered entirely online without face-to-face components and reported depression, anxiety, and stress as well as the effectiveness, patient satisfaction, and acceptability outcomes. Eligible studies included female participants aged 18 to 64 years (regardless of menopausal stage) [54,55], constituting more than 80% of the intervention group or being a distinct subgroup for which the outcomes were reported. There were no restrictions on race, ethnicity, or geographical location. Peer-reviewed articles reporting original research data were also eligible. Eligible comparators included usual care, including in-person interventions, and waitlists. Studies were eligible for inclusion in the meta-analysis if they had a fully female participant sample, were RCTs, and reported outcomes related to the effectiveness of the intervention for depression, anxiety, and/or stress. Studies reporting on fewer than 5 participants; fully qualitative studies with no quantitative outcomes related to depression, anxiety, and/or stress; commentaries; expert opinions; editorials; review articles; conference abstracts; case reports or series; policy briefs; preprints; non–peer-reviewed studies; studies for which the full text could not be retrieved; and studies not published in English were excluded.

**Table 1.** Inclusion and exclusion criteria.

	Inclusion criteria	Exclusion criteria
Population	At least 5 adult female participants, ≥80% female participants in the total sample, mean age ≥18 and ≤64 years (regardless of menopause stage) with any health condition using remote mental health care services in any geographical location	Fewer than 5 female adult participants,<80% female participants in the total sample
Intervention	Telemental health consultations involving both asynchronous communication and 2-way, synchronous patient-remote consultations delivered via, for example, the web, apps, telephone, text messages, or videoconference by primary care health care professionals	Nonremote consultations involving face-to-face or in-person care with remote care at the same time (hybrid approach setting)
Comparator	Standard care, in-person consultations, waitlists, prior in-person care experience (as measured by survey questions)	Other remote settings
Outcome	Studies presenting quantitative data with valid psychometric measuring tools for depression and anxiety pertaining to the primary outcome of effectiveness (eg, clinical or health outcomes) and secondary outcome of patient-centeredness, including measures of acceptability and satisfaction (eg, patient-reported satisfaction and improvements in health-related quality of life)	Qualitative studies and studies lacking quantitative evidence from validated psychometric instruments assessing depression and/or anxiety or participants’ satisfaction after treatment
Study type	English-language peer-reviewed studies of randomized controlled trials, cluster randomized trials, quasi-experimental design, case-control and cohort studies, cross-sectional analyses, and other clinical investigations	Studies reporting on fewer than 5 participants, commentaries, expert opinions, editorials, review articles, conference abstracts, case reports/series, policy briefs, preprints, non–peer-reviewed studies, studies for which the full text could not be retrieved, and studies not published in English

### Outcome Measurement Tools

The primary outcomes evaluated were symptoms of depression or anxiety or stress measured using validated tools. These tools included the Patient Health Questionnaire-9 (PHQ-9), Beck Depression Inventory (BDI), 7-item Generalized Anxiety Disorder Scale (GAD-7), Hospital Anxiety and Depression Scale (HADS), Center for Epidemiologic Studies Depression Scale, Hamilton Depression Rating Scale, Montgomery-Åsberg Depression Rating Scale (MADRS), Generalized Anxiety Disorder Severity Scale, Beck Anxiety Inventory (BAI), Generalized Anxiety Disorder Questionnaire-IV, Hamilton Anxiety Rating Scale, Leibowitz Social Anxiety Scale, Overall Anxiety Severity and Impairment Scale, stress subscale of the Depression Anxiety Stress Scales (DASS-S), Davidson Trauma Scale (DTS), 4-item version of the Perceived Stress Scale (PSS-4), and 14-item version of the Perceived Stress Scale (PSS-14).

Although the included studies reported a broader range of outcomes, this review focused on the primary outcomes, while all other relevant outcomes were summarized in a table.

### Data Extraction

Agreement about potential relevance was reached by consensus between the 2 reviewers, and in case of discrepancies, this was discussed and resolved by consultation with a third reviewer. A predefined data extraction form was used to gather information relevant to the title, first author, journal, year of publication, study type, and outcomes of the included studies. Moreover, relevant data regarding the study population, the intervention and control groups, timing or tool of outcome assessment, adverse events in each group, sample size, and mean and SD values were also recorded. In case of dichotomous observations, the relative risk estimates, effect size, and 95% CIs were noted. Moreover, reference lists of relevant original and review articles were hand-searched for additional reports. Detailed justifications for the exclusion of studies at each stage of screening were systematically documented in a spreadsheet. The literature was carefully checked to avoid duplicates and overlapping studies. In case of overlapping studies, the one with the largest sample size was finally retrieved.

### Risk of Bias Assessment

The quality assessment of all RCTs was conducted using the Cochrane risk-of-bias tool version 2 (RoB 2) [56], which comprises 5 key domains evaluating potential sources of bias in study design and conduct, including the randomization process, deviations from intended interventions, missing outcome data, measurement of the outcomes, and selective reporting of results. Each domain was assessed with respect to its potential impact on the validity of the findings.

For nonrandomized studies, quality assessment was performed using the Risk of Bias Assessment in Non-Randomized Studies of Interventions (ROBINS-I) tool [57]. This tool evaluates domains related to bias arising from participant selection, confounding variables, measurement, blinding, incomplete outcome data, and selective reporting of results.

Two reviewers (AMM and RS) independently assessed the quality of each of the 23 RCTs included in the meta-analysis, after previously evaluating the additional 12 RCTs that were excluded from the meta-analysis due to incomplete data. Two other reviewers (RS and SK) independently assessed the quality of the 22 nonrandomized studies included in the remainder of the review. The results of the assessment were graphically presented using the robvis [58] visualization tool.

### Statistical Data Analysis

Inclusion criteria for the meta-analysis included the following: (1) studies including women participants only; (2) studies with homogeneous outcome measures that could be combined statistically, including depression, anxiety, and/or stress; and (3) RCTs that compared a telehealth intervention with a waitlist or standard care condition. The relevant study characteristics, including the type of intervention, relevant mental health outcome(s), assessment time points, and mean baseline and postintervention scores across the key outcomes for both the control and the intervention groups were summarized.

A series of random-effects meta-analyses using standardized mean differences were conducted to calculate the overall effect estimate (Hedges *g*) with 95% CI for the 3 main outcome measures: depression, anxiety, and stress. The Hartung-Knapp-Sidik-Jonkman modification was applied to the model to reduce the rate of false positives and improve the precision of the weighted pooled average estimated [59]. The Hedges *g* effect size estimate was used because it tends to produce less biased results for studies with smaller samples and when sample sizes differ substantially between studies, in contrast with Cohen *d* [60]. Prediction intervals (95% PI) were also calculated to estimate the range of the true treatment effects in future similar studies, considering both the mean treatment effects and between-study heterogeneity [61]. For studies in which multiple telehealth intervention groups were compared with a single control group [62], we split the control group to avoid multiplicity and double counting of participants [63,64]. Statistical heterogeneity was tested using 95% PI and the *I*^2^ statistic. The telehealth intervention was considered effective if it showed statistically significant (*P*≤.05) treatment benefits for the relevant outcome compared to the control group and considered ineffective if no significant differences were found between the 2 groups (*P*≥.05). Subgroup analyses were conducted to determine the overall effect stratified by variables including the type of comparison group and the timing of the assessments. Small-study effects for each outcome of interest were assessed visually using funnel plots and statistically using Egger test [65]. Statistical analyses and forest plot diagrams were produced using the Stata software (version 16; StataCorp).

### Certainty of Evidence Assessment

The Grading of Recommendations, Assessment, Development, and Evaluation (GRADE) [66] approach was used to assess the quality of the evidence. This approach involves evaluating study design, risk of bias, inconsistency, indirectness, imprecision, and other relevant factors. Based on these assessments, the quality of the evidence was rated on a 4-point scale as high, moderate, low, or very low.

In the present study, the GRADE quality assessment and summary of findings table [67] presents the certainty of evidence for outcomes derived from both nonrandomized studies and studies included in the meta-analysis. GRADE assessments were conducted at the outcome level after the synthesis of the available evidence.

## Results

### Study Selection

**Figure 1.**
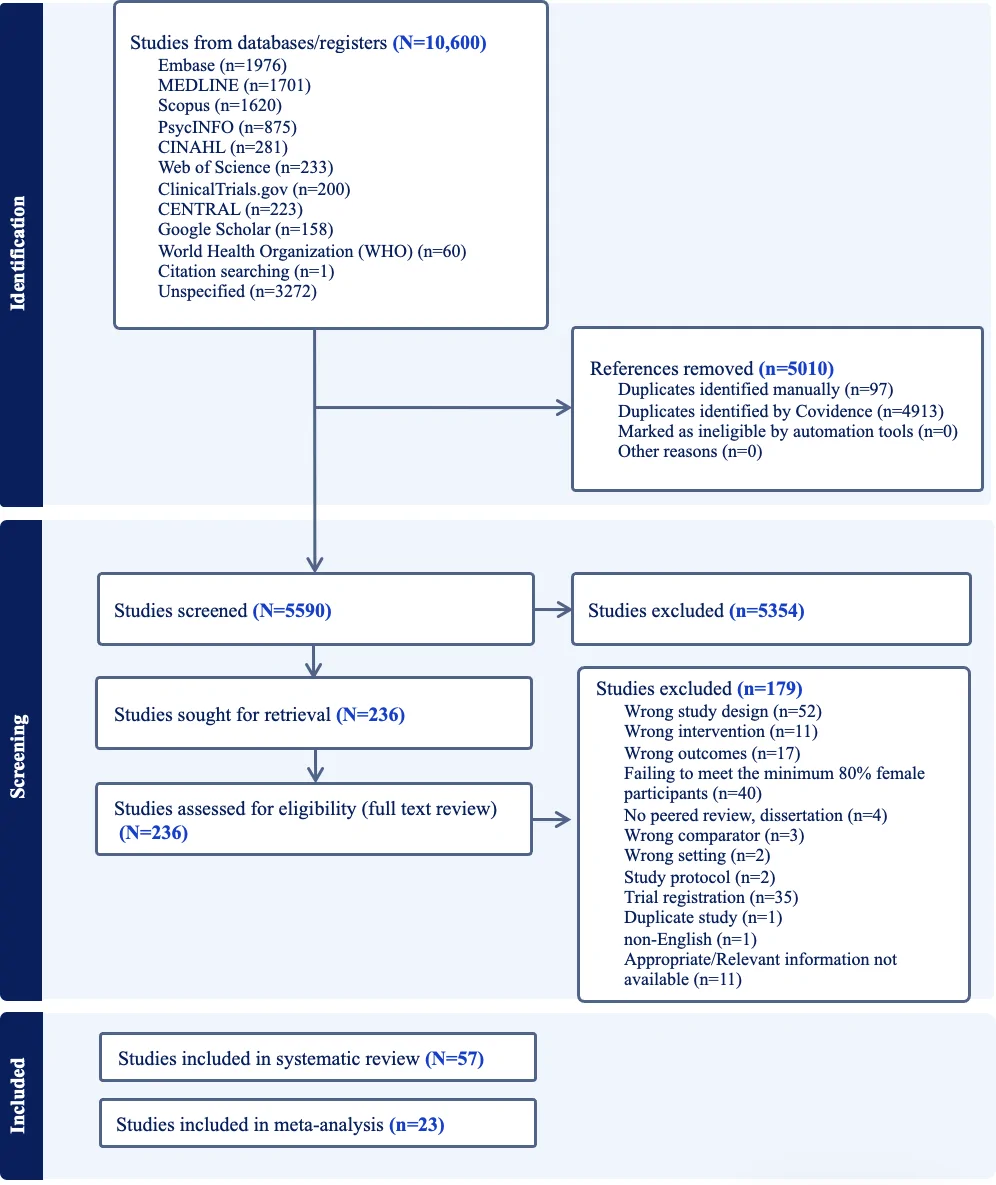
PRISMA flow diagram of included/excluded studies. CENTRAL: Cochrane Central Register of Controlled Trials.

The initial search identified 10,600 reports, and 1 additional study was included through citation searching. Following duplicate removal and screening for full-text reports, 57 studies were included in this systematic review (Table 2), and 23 studies were included in the meta-analysis [62,63,68-88]. The detailed PRISMA flowchart is presented in Figure 1.

**Table 2.** Effectiveness, Satisfaction, and Acceptability with telemental healthcare interventions for women during and after COVID-19 Pandemic, by telehealth delivery mode.

Study	Intervention delivery mode	Effectiveness	Satisfaction	Acceptability
Arias et al [89]	Web-based synchronous	Effective	—^a^	—
Bantjes et al [90]	Videoconferencing synchronous	Effective	High	—
Bäuerle et al [91]	Web-based education asynchronous	Effective	High	—
Boorboor et al [92]	Videoconferencing, synchronous	Effective	—	—
Brooks et al [93]	Videoconferencing, synchronous	Effective	—	Acceptable
Cavarretta et al [94]	Videoconferencing, synchronous	Effective	—	—
Doornbos et al [95]	Videoconferencing, synchronous	Effective	Highly satisfied	—
Farris et al [96]	Web-based video asynchronous	Effective	—	—
Gemmill et al [97]	Video, phone, synchronous	Effective	87% moderately to very helpful	Moderately to highly acceptable
González-García et al [98]	Videos on web-based training, asynchronous	Effective	High	Acceptable
Hahn et al [99]	Mobile app, asynchronous	Effective	—	—
Haro-Ramos et al [100]	Text message, asynchronous	Effective	—	—
He et al [101]	Messaging app, synchronous	Effective	—	—
Iravani et al [102]	Phone and text, synchronous	Effective	—	—
Langhammer et al [103]	Chatbot, synchronous		—	—
Lestari and Anindya [104]	Messaging app, synchronous	Effective	—	—
Liu et al [105]	Messaging app, synchronous	Effective	90.4% Extremely satisfied	Acceptable
Merza et al [106]	Videoconferencing, synchronous	Effective	Mean 24.48 (SD 4.78) indicating adequate satisfaction	Acceptable
Ornelas et al [107]	App, synchronous	Effective	High	Acceptable
Valinskas et al [108]	Mobile app, asynchronous	Effective	—	—
Van Haeken et al [109]	Mobile app and phone, synchronous	Not effective	—	—
Young et al [110]	Web-based and phone, synchronous	Effective	—	Acceptable
Aligoltabar et al [111]	Web-based, synchronous	Effective	—	No difference between groups
Boucher et al [112]	Web-based, asynchronous	Effective	—	—
Bryant et al [68]	Videoconferencing, synchronous	Effective	—	Acceptable
Chang et al [69]	Videoconferencing, synchronous	Effective	—	—
De Kock et al [70]	Mobile app, synchronous	Effective	—	—
Dominguez-Rodriguez et al [71]	Web-based, asynchronous	Effective	No difference	Acceptable
Dominguez-Rodriguez et al [72]	Web-based, asynchronous	Effective	High	—
Dumarkaite et.al [113]	Web-based, synchronous	Effective	High	—
Ehlis et al [114]	Web-based SNS, asynchronous	Effective	—	—
Fiol-DeRoque et al [73]	Mobile App, synchronous	Effective	—	Highly acceptable
Gomà et al [74]	Videoconferencing, synchronous	Effective	—	—
Guerrero-Pertiñez et al [115]	Web-based, asynchronous	Effective	—	—
Güney et al [75]	Web-based, synchronous	Effective	—	—
Jung and Ko [116]	Phone, synchronous	Effective	—	—
Karing [76]	Videoconferencing, synchronous	Effective	Satisfied	—
MacKinnon et al [117]	Mobile app, synchronous	Effective	Most respondents felt satisfied	Acceptable
Naja et al [77]	Video, synchronous	Effective	—	—
Pratt et al [78]	Mobile app, asynchronous	Effective	—	—
Puertas-Gonzalez et al [63]	Web-based, synchronous	Effective	—	—
Rahgozar et al [118]	Web-based, synchronous	Effective	—	—
Ruzickova et al [119]	Videoconferencing, synchronous	Effective	—	Highly acceptable
Sockalingam et al [79]	Phone, synchronous	Effective	—	—
Shahamabadi et al [80]	Videoconferencing, synchronous	Effective	—	—
Smith et al [81]	Mobile app, asynchronous	Effective	More than 80% high satisfaction in intervention group	—
Ugarte et al [120]	Facebook, asynchronous	Effective	—	—
Van Lieshout et al [82]	Videoconferencing, synchronous	Effective	—	—
Vlassopoulos et al [121]	Phone, synchronous	Effective	—	—
Wahlund et al [62]	Web-based, asynchronous	Effective	High satisfaction	—
Wang et al [122]	Videoconferencing, synchronous	Effective	—	—
Yook et al [83]	Videoconferencing, synchronous	Effective	—	—
Zhang et al [84]	Videoconferencing, synchronous	Effective	—	—
Zhou et al [85]	Web-based, synchronous	Effective	—	—
Zhang et al [86]	Web-based, synchronous	Effective	—	—
Wi et al [87]	Web-based, asynchronous	Effective	Intervention vs control: 32.8 ± 5.1 vs 30.1 ± 6.9	Acceptable
Duggal et al [88]	Phone, synchronous	Effective	High satisfaction	Well accepted

a Not applicable.

### Study Characteristics

A detailed description of the included studies is presented in Multimedia Appendix 2. All included studies were published between 2020 and 2025 and were conducted across 24 countries. Countries with the largest number of eligible studies included the United States (n=9); China (n=6); Germany (n=6); Spain (n=6); Canada (n=5); Iran (n=4); the United Kingdom (n=3); and Australia, France, India, South Korea, Lithuania, and Mexico (n=2 each). Countries and regions represented by 1 study each included Belgium, Bangladesh, Hong Kong, South Africa, Taiwan, Turkey, Qatar, Indonesia, Sweden, Austria, Italy, and India. Most studies were RCTs (n=35). Sample sizes ranged between 12 [93] and 2402 [121] participants, with a total of 13,457 participants across all 57 studies. Of the 57 studies, 27 studies included only female participants, whereas 30 studies included a mixed-sex population with over 80% of the participants being women. The age of participants varied considerably and was reported heterogeneously across studies. For example, studies conducted with university students generally reported younger mean ages (ie, early 20s) than studies conducted with pregnant women (late 20s to mid-30s).

A total of 57 studies were included in this review, all of which investigated mild to moderate mental health symptoms, either self-reported or assessed using valid, standardized measurement instruments. These included but were not limited to the Edinburgh Postnatal Depression Scale (EPDS), State-Trait Anxiety Inventory (STAI), Patient-Reported Outcomes Measurement Information System (PROMIS), and PHQ-9 questionnaires. Participants were recruited based on self-reported preexisting mental health conditions or based on scores from validated questionnaires for depression, stress, and anxiety. In contrast, a total of 7 studies focused on participants without specific report or presence of any preexisting mental health conditions.

### Summary of Intervention Types and Outcomes

The studies included in this systematic review used a variety of digital mental health interventions, all of which were delivered online. Among the 57 studies, 40 interventions were synchronous, and 17 were asynchronous. Of the 57 studies, 43 studies were guided, and 14 were unguided. Most interventions were conducted in real time, synchronously, using video conferencing or other web-based platforms or text messages and phone calls. A smaller number of studies used self-paced or asynchronous formats, such as web or messaging app modules. A few studies provided more formal therapeutic programs such as cognitive behavioral therapy (CBT), behavioral activation therapy, or some form of mindfulness training for a period between 2 and 14 weeks, whereas others provided loose and more informal support as and when required. Session frequency also varied widely across the interventions, ranging from a single session to weekly and even multisession formats (eg, 4 sessions structured in 4 weeks or weekly sessions over several weeks). Participants reported the duration of individual sessions to range from 30 minutes to 60 minutes. These programs were primarily designed to enhance mental health outcomes and incorporated both individual and group elements.

Outcome data related to depression and/or anxiety were reported in all 57 studies included in this systematic review. All studies used validated screening tools such as the PHQ-9, GAD-7, BDI, DASS, EPDS, STAI, BAI, PSS, Client Satisfaction Scale (CSS), Patient Participation Questionnaire (PPQ), Symptom Checklist-90-Revised (SCL-90-R), Center for Epidemiologic Studies Depression Scale—Revised (CESD-R), MADRS, PROMIS Depression Short Form 6a (PROMIS-D-6a), PROMIS Anxiety Short Form 6a (PROMIS-Anx6a), and HADS questionnaires to assess outcomes. Of the 57 studies, 42 studies assessed depression, 45 assessed anxiety, 23 assessed stress, and 36 assessed both anxiety and depression. Notably, 56 of the 57 studies reported that the telemental health interventions were effective and reported significant reductions in depression and/or anxiety symptoms, as well improvements in psychological distress, emotional regulation, and coping. Moreover, 22 studies explicitly reported user acceptability or satisfaction and engagement metrics, consistently indicating positive engagement with the interventions.

### Effectiveness Outcome

Of the 57 studies included in this review, 56 reported positive effects of THIs, with the majority demonstrating high levels of effectiveness across assessed domains.

Across the included studies, the majority demonstrated beneficial effects, particularly in reducing the symptoms of depression, anxiety, and stress. Most interventions were described as effective or associated with improvements in psychological well-being, emotional regulation, resilience, and quality of life. For instance, Dominguez-Rodriguez et al [72] reported significant reductions in clinical symptoms across all measured variables in the intervention group (*P*<.001 to *P*=.006), with larger effect sizes observed for depression, hopelessness, grief disorder, and anxiety (all effect sizes ≥0.5). Similarly, Aligoltabar et al [111] found that the interventions led to significant improvements in anxiety symptoms, pregnancy-specific stress, and emotional regulation. These effects were observed across a wide range of intervention formats, including CBT; mindfulness-based interventions; and support delivered via videoconferencing, mobile apps, and web-based platforms [75,90,91,118,122].

A smaller number of studies reported partial or context-dependent effects. For example, some interventions showed improvements only in specific outcomes or populations or effects that were not sustained over time [73,78,116]. In addition, a few studies highlighted limitations such as reduced adherence over the course of the intervention or benefits restricted to specific subgroups [99,112].

In contrast to these findings, Van Haeken et al [109] was the only study in which the intervention did not demonstrate effectiveness. The authors evaluated an online intervention aimed at enhancing resilience and promoting maternal mental health among pregnant women, which included structured exercises, group sessions, and platform-based support. However, the intervention did not demonstrate significant effectiveness in improving resilience, as the intervention group showed stable resilience levels. Within the intervention group, perceived social support remained stable during the intervention period but decreased significantly at the first follow-up. Jung and Ko [116] also found conventional face-to-face care to be significantly more effective than psychiatric telerehabilitation on both the Zung Self-Rating Anxiety Scale and the Visual Analogue Scale (VAS) for depression, but the psychiatric telerehabilitation intervention was still deemed as effective. Finally, Hahn et al [99] found no significant effects for the “7mind” app as an intervention for only depression based on EPDS scores. All other interventions, such as group-based CBT, mobile app–based mindfulness, and online group logotherapy, were deemed effective in reducing anxiety and stress, as well as mild to moderate depression.

### Acceptability and Satisfaction Outcomes

As part of this systematic review, user acceptability and engagement with telemental health interventions were also considered, Overall, findings indicated high levels of acceptability and satisfaction among participants.

Of the 57 studies included, 17 reported satisfaction outcomes, with at least 14 indicating that most participants experienced moderate to high levels of satisfaction. For example, Gemmill et al [97] found that 87% of participants rated the online CBT program as moderately to very helpful. Similarly, Liu et al [105] reported that 90.4% of mothers in the intervention group were extremely satisfied with the WeChat-based parenting training.

In terms of acceptability, 15 studies reported this outcome, of which 14 described the interventions as acceptable or highly acceptable. For instance, Bantjes et al [90] found that 91.1% of participants rated the quality of their group-based CBT intervention as good or excellent, 95.2% stated that they would recommend it to others, and 89.6% reported being better able to manage their problems following the intervention. Across studies, interventions such as group-based CBT, mobile app–based mindfulness, and online group logotherapy were consistently deemed acceptable.

Additional evidence further supports high participant engagement and perceived value. Wi et al [87] reported higher satisfaction scores among pregnant women in the intervention group (32.8 ± 5.1) compared with the control group (30.1 ± 6.9), alongside reductions in psychological distress, fatigue, and depressive symptoms. Duggal et al [88] found that mobile phone counseling was well received by Indian women living with HIV, with higher postpartum call attendance rates in the intervention group (91%) compared with the control group (77%), and 94% of participants evaluating the intervention positively. Similarly, Langhammer et al [103] observed a significant increase in perceived intervention benefits, rising from 49% at initiation to 95% upon program completion. However, not all studies reported detailed satisfaction outcomes. For example, although Aligoltabar et al [111] used the Client Satisfaction Questionnaire (CSQ-8), no specific results were presented, and the authors concluded that there was no significant difference in acceptability between the 2 internet-based emotion-focused CBT (IECBT) approaches tested.

Overall, these findings suggest strong participant engagement, high acceptability, and perceived value of telemental health interventions, particularly when human support and program completion are emphasized.

### Risk of Bias Assessment

**Figure 2.**
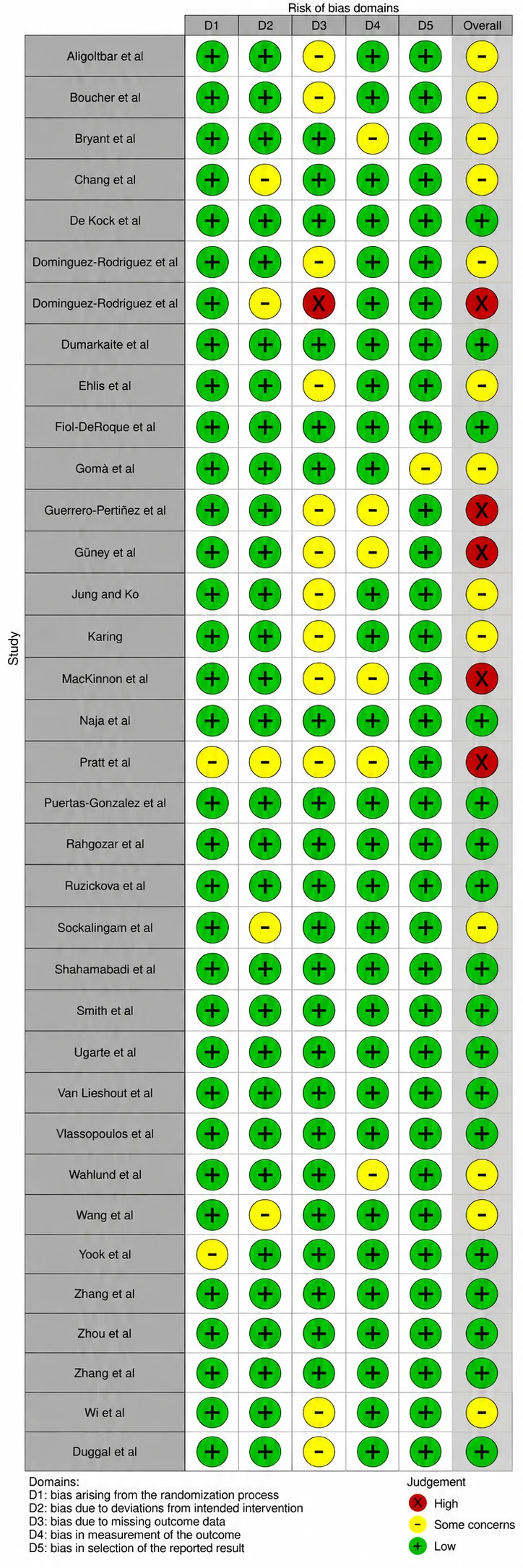
Risk of Bias (RoB 2) assessment of randomized controlled trials visualized using the robvis tool [62,63,68-88,111-122].

**Figure 3.**
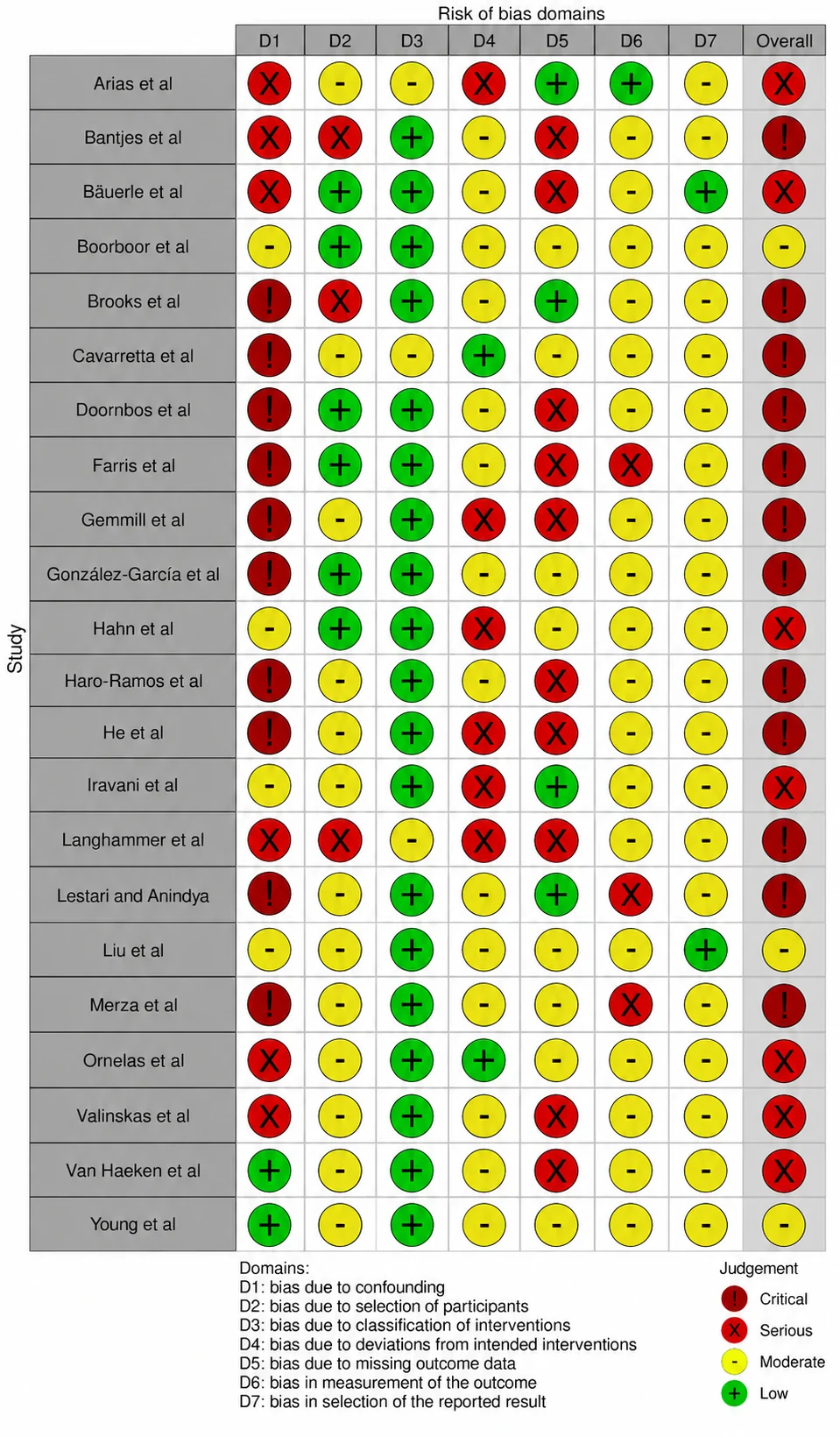
Quality assessment of the nonrandomized studies using Risk of Bias Assessment in Non-Randomized Studies of Interventions (ROBINS-I) [89-110].

The ROBINS-I [57] and RoB 2 [56] tools were used to assess the methodological quality of individual studies [123]; the assessment of the included studies is presented in Figure 2 and Figure 3, respectively. Detailed risk of bias assessments for the included studies are provided in Tables S1 and S2 in Multimedia Appendix 3. Thirty-five studies were evaluated using RoB 2 robvis (Figure 2). Of these 35 studies, 16, 14, and 5 were deemed to have low concerns, some concerns, and high concerns, respectively. Twenty-two studies were graded using ROBINS-I (Figure 3). Of these 22 studies, 12, 7, and 3 were deemed to have a critical, serious, and moderate risk of bias, respectively, and none were graded as having a low risk of bias.

The risk of bias assessment for the included RCTs using RoB 2 showed that the overall risk of bias was low to moderate and the main cause for potential bias was due to difficulty in the blinding of participants and personnel. This occurred due to the use of self-reported surveys for evaluating mental health outcomes that could not be blinded and limited clinical assessment of outcomes due to the COVID-19 pandemic.

### Meta-Analysis Findings

#### Included Studies

**Figure 4.**
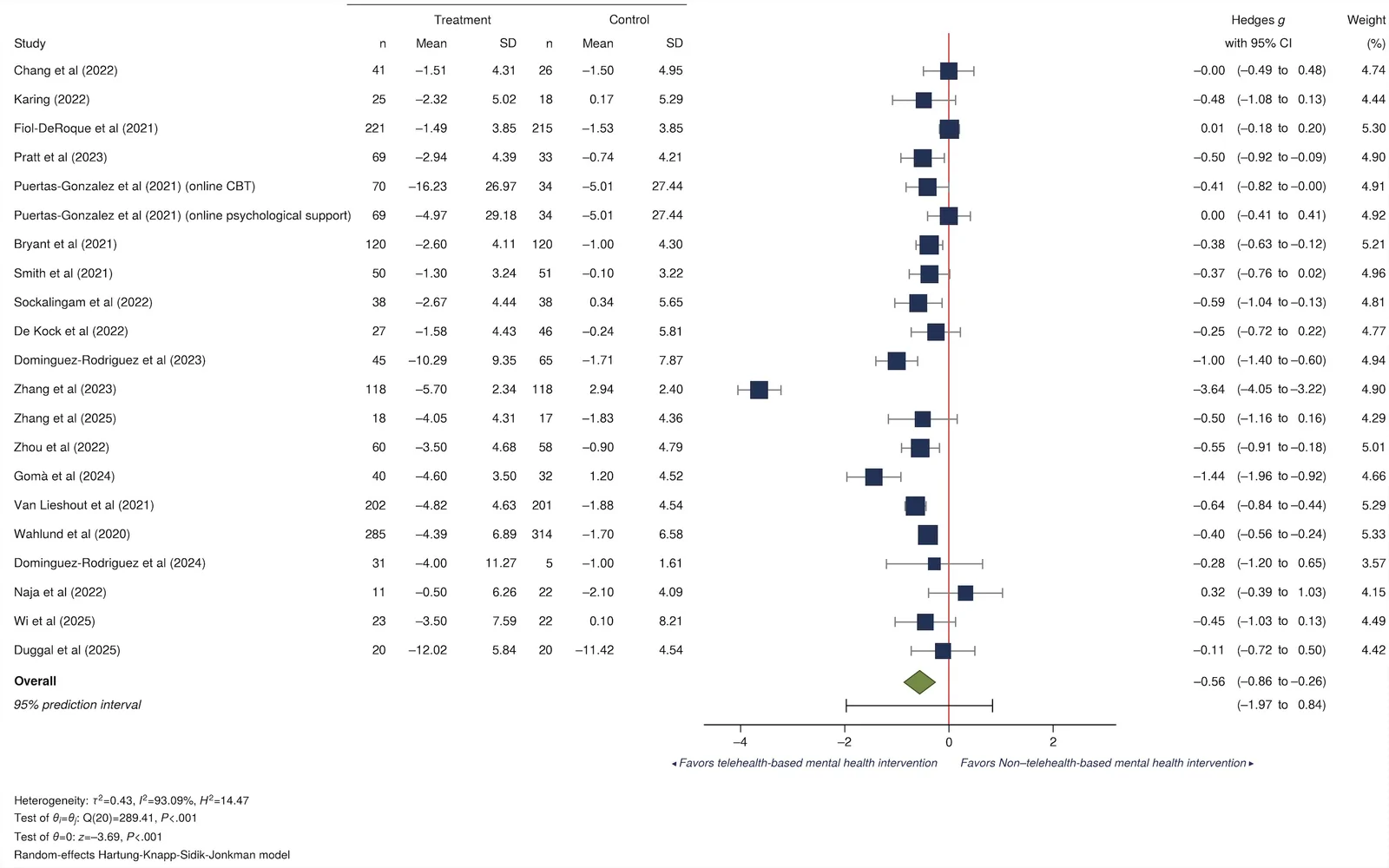
Forest plot showing the effects of online mental health interventions on depression outcomes [62,63,68-74,76-79,81,82,84-88].

The statistical analysis for the meta-analysis was performed using Review Manager Version 7.2.0; he Cochrane Collaboration and Stata. We conducted sensitivity and subgroup analyses to examine the consistency of our results. Specifically, we compared the outcomes of the overall analysis, which included studies with more than 80% women participants, to the results obtained when only studies with 100% women participants were considered. Through subgroup analysis, we also compared studies with 100% women participants to those with 80% to 99% women participants. This analysis was undertaken to assess the robustness of the findings while broadening inclusion to predominantly female populations. A total of 23 RCTs (N=3269 participants) were included in the meta-analyses, and 4 were rated as having a high risk of bias based on the Cochrane Risk of Bias tool (studies presented in Figure 4). Of these, 20 RCTs assessed depression (n=3072 participants), 18 assessed anxiety (n=2780 participants), and 9 assessed stress (n=1262 participants).

#### Effectiveness for Depression

The overall treatment effect for depression was significant (Hedges *g*=−0.56, 95% CI −0.86 to −0.26; *P*<.001), although considerable between-study heterogeneity was observed (95% PI −1.97 to 0.84 and *I*^2^=93.1%, *P*<.001), as shown in Figure 4. A nonsignificant Egger test (intercept −0.70, SE 1.77; *t*=0.40, *P*=.69) indicated no evidence for small-study effects, but the corresponding funnel plot revealed that the effect size reported by Zhang et al [86] constituted an outlier compared with the other studies (see Figure S1 in Multimedia Appendix 4). As a result, a sensitivity analysis was performed in which the study by Zhang et al [86] was excluded; while the overall treatment benefit of telehealth for depression decreased slightly (Hedges *g*=−0.41, 95% CI −0.56 to −0.26; *P*<.001), it remained statistically significant, supporting the robustness of the primary analysis (see Figure S2 in Multimedia Appendix 4).

In subgroup analyses, significant effects of online mental health interventions were found when compared with waitlist conditions, in which participants received no treatment (Hedges *g*=−0.60, 95% CI −0.86 to −0.33; *P*<.001), and in comparison with standard care interventions (Hedges *g*=−0.57, 95% CI −1.11 to −0.02; *P*<.001) (Figure S3 in Multimedia Appendix 4). Considerable heterogeneity was observed in both subgroups (95% PI −1.44 to 0.24 and *I*^2^=77.7%, *P*<.001, for studies with a waitlist comparator and 95% PI −2.73 to 1.59 and *I*^2^=95.8%, *P*<.001, for studies with a standard care comparator).

On average, studies with an all-female sample showed slightly greater improvements in anxiety outcomes (Hedges *g*=−0.65, 95% CI −1.20 to −0.10; *P*<0.01) than studies with 80% to 99% female participants (Hedges *g*=−0.44, 95% CI −0.66 to −0.22; *P*<0.01), but both subgroups’ benefits were statistically significant (see Figure S4 in Multimedia Appendix 4). Considerable heterogeneity was observed in both subgroups (95% PI −1.12 to 0.24 and *I*^2^=71.3%, *P*=.001, in the subgroup of studies with 80%‐99% female participants and 95% PI −2.82 to 1.52 and *I*^2^=95.4%, *P*<.001, in the subgroup of studies with all-female participants).

Subgroup analyses were also performed to test the influence of assessment time points on the treatment benefit. The overall effect of online mental health interventions was significant when depression was assessed ≤1 month post intervention (Hedges *g*=−0.26, 95% CI −0.42 to −0.10; *P*<.001) and when assessed at 1 to 3 months post intervention (Hedges *g*=−0.83, 95% CI −1.46 to −0.20; *P*<.001) (Figure S5 in Multimedia Appendix 4). Considerable heterogeneity was observed in the 1 to 3 months subgroup (95% PI −3.18 to 1.52 and *I*^2^=96.2%, *P*<.001), and moderate heterogeneity was seen in the ≤1 month subgroup (95% PI −0.67 to 0.15 and *I*^2^=44.7%, *P*=.05).

Subgroup analyses based on other participant-related or study design–related factors were not possible, given the high heterogeneity in study characteristics.

#### Effectiveness for Anxiety

**Figure 5.**
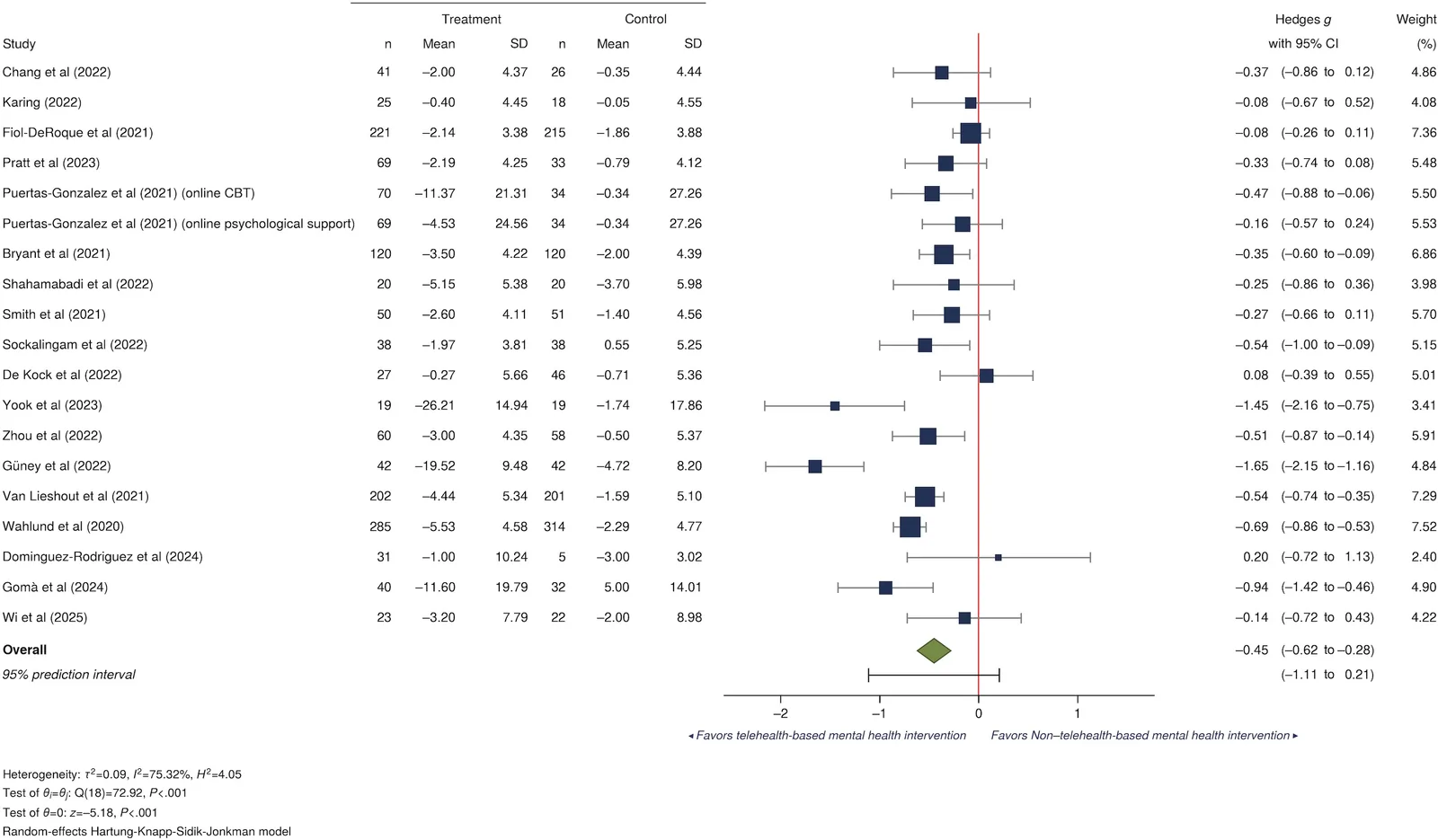
Forest plot showing the effects of online mental health interventions on anxiety outcomes [62,63,68-71,73-76,78-83,85,87].

The overall treatment effect for anxiety was significant (Hedges *g*=−0.45, 95% CI −0.62 to −0.28; *P*<.001*)*, although a considerable level of between-study heterogeneity was observed (95% PI −1.11 to 0.21 and *I*^2^=75.3%, *P*<.001) as shown in Figure 5. A nonsignificant Egger test (intercept −0.11, SE 1.05; *t*=−0.10, *P*=.93) indicated no evidence for small-study effects (see Figure S6 in Multimedia Appendix 4 for the corresponding funnel plot).

In subgroup analyses, similar statistically significant effects of online mental health interventions were found both in studies using a waitlist control group, in which participants received no treatment (Hedges *g*=−0.47, 95% CI −0.68 to −0.25; *P*<.001), and when compared with standard care interventions (Hedges *g*=−0.52, 95% CI −0.80 to −0.25; *P*<.001) (Figure S7 in Multimedia Appendix 4), while both subgroups displayed significant heterogeneity (95% PI −1.08 to 0.14 and *I*^2^=62.5%, *P*=.014, for waitlist, and 95% PI −1.46 and 0.42 and *I*^2^=81.2%, *P*<.001, for standard care). Comparison with other types of control conditions (eg, face-to-face interventions) was not feasible due to the small number of studies with these characteristics.

Studies with an all-female sample showed slightly greater improvements in anxiety outcomes (Hedges *g*=−0.58, 95% CI −0.82 to −0.22; *P*<0.01) than studies with 80% to 99% female participants on average (Hedges *g*=−0.30, 95% CI −0.56 to −0.04; *P*<0.01), but both subgroups’ benefits were statistically significant (see Figure S8 in Multimedia Appendix 4). Considerable heterogeneity was observed in both subgroups (95% PI −1.40 to 0.24 and *I*^2^=77.3%, *P*<.001, in the subgroup of studies with 80%‐99% female participants and 95% PI −1.11 to 0.51 and *I*^2^=74.4%, *P*<.001, in the subgroup of studies with all-female participants).

Additionally, similar statistically significant benefits were seen in both studies assessing anxiety 1 to 3 months post intervention (Hedges *g*=−0.43, 95% CI −0.55 to −0.31; *P*<.01) and studies assessing stress ≤1 month post intervention (Hedges *g*=−0.09, 95% CI −0.48 to −0.14; *P*<.01) (see Figure S9 in Multimedia Appendix 4). There was no heterogeneity in the 1‐ to 3-month assessment group (95% PI −0.57 to −0.29 and *I*^2^=0%, *P*=.75), while considerable heterogeneity was present in the ≤1 month group (95% PI −1.65 to 0.69 and *I*^2^=87.4%, *P*<.001), which included both studies showing reductions [70,83] and increases in anxiety levels post intervention [71].

Subgroup analyses based on other participant-related or study design–related factors were not possible, given the high heterogeneity in study characteristics.

#### Effectiveness for Stress

**Figure 6.**
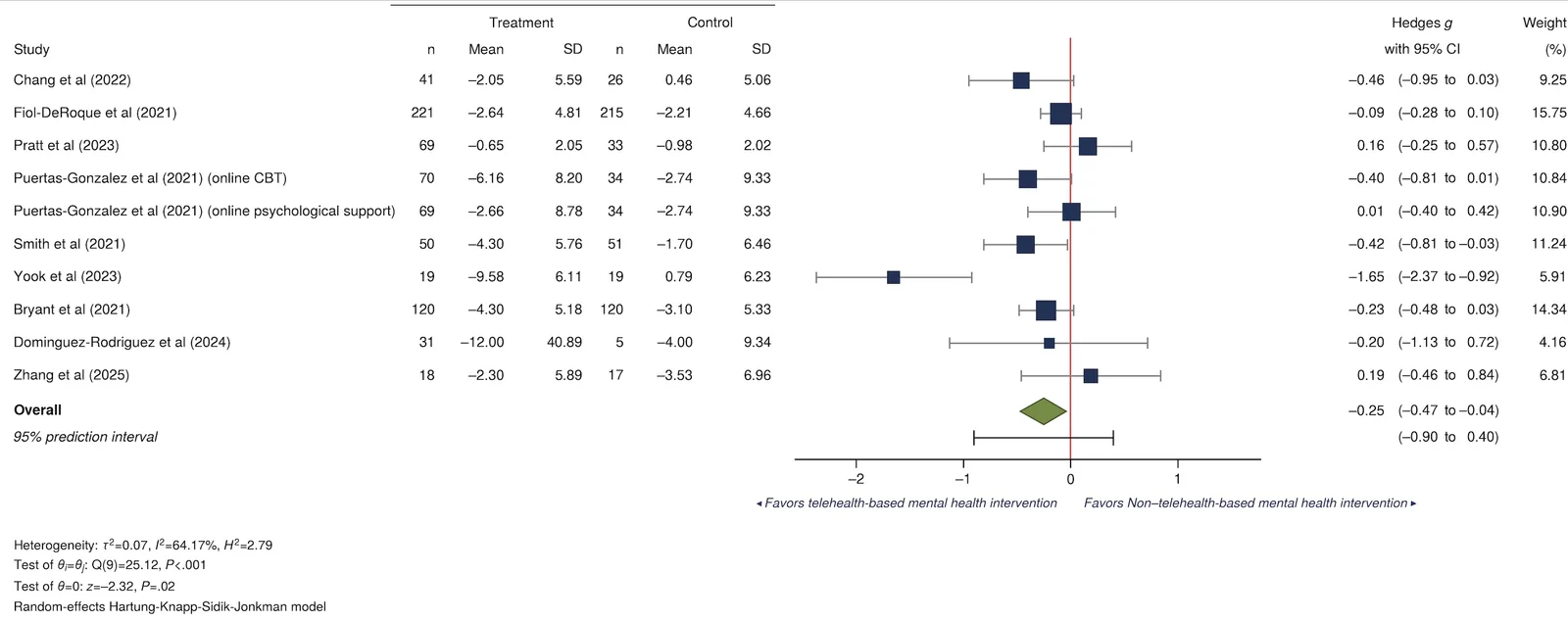
Forest plot showing the effects of online mental health interventions on stress outcomes [63,68,69,71,73,78,81,83,86].

Finally, THIs showed a significant treatment effect on stress among women (Hedges *g*=−0.25, 95% CI −0.47 to −0.04; *P*=.02), although considerable between-study heterogeneity was observed (95% PI −0.98 to 0.40 and *I*^2^=64.2%), as presented in Figure 6. A nonsignificant Egger test (intercept −1.50, SE 1.20; *t*=−1.26, *P*=.21) indicated no evidence for small-study effects (see Figure S10 in Multimedia Appendix 4 for the corresponding funnel plot). However, it should be noted that, due to the relatively small number of studies, the test could be underpowered, and thus results are considered exploratory.

In subgroup analyses, studies with an all-female sample showed greater average improvements in stress outcomes (Hedges *g*=−0.37, 95% CI −0.67 to −0.08; *P*=.01) than studies with 80% to 99% female participants, which showed no significant benefits (Hedges *g*=−0.05, 95% CI −0.22 to 0.12; *P*=.53). However, the relatively small number of studies with 80% to 99% female participants limits the robustness of this analysis; therefore, these findings should be considered exploratory (see Figure S11 in Multimedia Appendix 4). No heterogeneity was observed in studies with 80% to 99% female participants (95% PI −1.15 to 1.05 and *I*^2^=0%, *P*=.53), while studies with all-female participants showed considerable heterogeneity (95% PI −1.27 to 0.53 and *I*^2^=68.0%, *P*=.01).

Additionally, studies in which stress outcomes were assessed 1 to 3 months post intervention showed greater stress reduction on average (Hedges *g*=−0.46, 95% CI −0.84 to −0.07; *P*=.003) compared to studies assessing stress ≤1 month post intervention, which showed no significant benefits (Hedges *g*=−0.09, 95% CI −0.29 to 0.10; *P*=.30) (see Figure S12 in Multimedia Appendix 4). This pattern is generally common in studies of psychological interventions, as therapeutic effects may take time to develop. Heterogeneity was lower in the ≤1-month assessment group (95% PI −0.53 to 0.35 and *I*^2^=18.7%, *P*=.30 vs 95% PI −1.79 to 0.87 and *I*^2^=75.4%, *P*=.003 for studies assessing stress at 1‐3 months).

Subgroup analyses based on other participant-related or study design–related factors were not possible, given the limited number of studies.

### Quality of Evidence

The GRADE quality assessment was used to assess the certainty of evidence for the outcomes reported in the RCTs and the nonrandomized studies (non-RCTs) included in the meta-analysis (Table 3) [66]. All studies were independently assessed using the GRADE approach before their results were combined in the meta-analysis. Of the studies included in the meta-analysis, 4 reported a very low-certainty outcome, 9 reported a low-certainty outcome, 12 reported a moderate-certainty outcome, and 8 reported a high-certainty outcome. Of the non-RCTs, 5 studies reported a very low-certainty outcome, 6 reported a low-certainty outcome, 5 studies reported a moderate-certainty outcome, and 4 studies reported a high-certainty outcome. Outcomes were limited to depression, anxiety, and stress. According to the GRADE assessment, the outcomes in the meta-analysis had moderate certainty, and the outcomes in the nonrandomized studies had low certainty. The downgrading factors included inconsistency—because heterogeneity was substantial—and very serious risk of bias for the nonrandomized studies.

**Table 3.** Summary of findings of the Grading of Recommendations, Assessment, Development and Evaluation (GRADE) assessment^a^.

Outcome	Anticipated absolute effects^b^ (95% CI)		Relative effect (95% CI)	Number of participants (studies)	Certainty of the evidence (GRADE)	Comments
	Risk with control conditions	Risk with remote telehealth intervention for mental health				
Depression: RCT^c^ meta-analysis	—	SMD^d^ 0.56 SD lower (0.86 lower to 0.26 lower)	—	3072 (20 RCTs)	⨁⨁⨁◯ Moderate^e^	Telehealth interventions probably reduce depression symptoms compared with control conditions.
Anxiety: RCT meta-analysis	—	SMD 0.45 SD lower (0.62 lower to 0.28 lower)	—	2780 (18 RCTs)	⨁⨁⨁◯ Moderate^f^	Telehealth interventions probably reduce anxiety symptoms compared with control conditions.
Stress: RCT meta-analysis	—	SMD 0.25 SD lower (0.47 lower to 0.04 lower)	—	1262 (9 RCTs)	⨁⨁⨁◯ Moderate^g^	Telehealth interventions probably reduce stress symptoms compared with control conditions.
Depression: NRSI^h^	Most nonrandomized studies reported reductions in depressive symptoms following the intervention.		—	1732 (14 nonrandomized studies)	⨁⨁◯◯ Low^i^	Telehealth interventions may reduce depressive symptoms, but the evidence is uncertain due to risk of bias limitations in the nonrandomized studies.
Anxiety: NRSI	Most nonrandomized studies reported reductions in anxiety symptoms following the intervention.		—	2358 (19 nonrandomized studies)	⨁⨁◯◯ Low^j^	Telehealth interventions may reduce anxiety symptoms, but the evidence is uncertain due to risk of bias and heterogeneity limitations in the nonrandomized studies.
Stress: NRSI	Some nonrandomized studies reported reductions in stress symptoms following the intervention.		—	1010 (7 non randomized studies)	⨁⨁◯◯ Low^f^	Telehealth interventions may reduce stress symptoms, but the evidence is uncertain due to risk of bias and imprecision limitations in the nonrandomized studies.

a GRADE Working Group grades of evidence: (1) high certainty: we are very confident that the true effect lies close to that of the estimate of the effect; (2) moderate certainty: we are moderately confident in the effect estimate; the true effect is likely to be close to the estimate of the effect, but there is a possibility that it is substantially different; (3) low certainty: our confidence in the effect estimate is limited; the true effect may be substantially different from the estimate of the effect; (4) very low certainty: we have very little confidence in the effect estimate; the true effect is likely to be substantially different from the estimate of effect.

b The risk in the intervention group (and its 95% CI) is based on the assumed risk in the comparison group and the relative effect of the intervention (and its 95% CI).

c RCT: randomized controlled trial.

d SMD: standardized mean difference.

e Downgraded 1 level for inconsistency because between-study heterogeneity was substantial and the 95% prediction interval crossed the null effect (95% PI −1.97 to 0.84; *I*2=93.1%).

f Downgraded 1 level for inconsistency because between-study heterogeneity was substantial and the 95% prediction interval crossed the null effect (95% PI −1.11 to 0.21; *I*2=75.3%).

g Downgraded 1 level for inconsistency because between-study heterogeneity was substantial and the 95% prediction interval crossed the null effect (95% PI −0.98 to 0.40; *I*2=64.2%).

h NRSI: nonrandomized studies of interventions.

i Downgraded for risk of bias because most studies were nonrandomized and had methodological limitations.

j Downgraded for risk of bias and heterogeneity because the evidence came from nonrandomized studies with methodological limitations.

## Discussion

### Principal Findings

This systematic review and meta-analysis examined the effectiveness, acceptability, and user satisfaction of telehealth-based mental health interventions among women. The review included 57 studies (N=13,457) conducted across 24 different countries. Although only 23 RCTs (n=3269) met the inclusion criteria for the meta-analysis, the pooled analyses provided clinically informative estimates of intervention effectiveness and important insights into the contexts in which THIs may be most beneficial. Overall, THIs were associated with improvements in depression, anxiety, and stress outcomes. However, the strength and consistency of the evidence varied across outcomes. The findings provide stronger support for improving depression and anxiety outcomes, whereas the evidence for stress remains more limited and should be interpreted with greater caution.

Subgroup analyses provided additional insights into the conditions under which THIs may be beneficial. Positive effects were observed relative to both waitlist and standard care comparators and were generally consistent in both female-only and predominantly female samples. However, there were insufficient studies to conduct comparable subgroup analyses for stress. These findings support the potential applicability of THIs across a range of women-centered populations and interventions.

However, the overall findings should be interpreted considering the heterogeneity and certainty-of-evidence assessments. Considerable variability was observed across studies, suggesting that intervention effects may differ according to population characteristics, implementation settings, intervention characteristics, and contextual factors. Although the evidence generally supports the effectiveness of THIs for depression and anxiety among women, methodological limitations and between-study heterogeneity reduce confidence that the observed average effects will be reproduced uniformly across all settings. Findings relating to stress are particularly preliminary because they were derived from a smaller evidence base and thus were associated with greater uncertainty.

Beyond effectiveness, the systematic review consistently demonstrated high levels of participant satisfaction, acceptability, and engagement with telemental health interventions. Across studies reporting outcomes, participants generally reported positive user experiences and perceived THIs as useful and acceptable, suggesting that these interventions are feasible and generally well accepted across diverse clinical and community settings. These findings have important clinical implications. Telehealth may help increase access to psychological support for women facing barriers to in-person care, including geographical constraints, caregiving responsibilities, disability, limited service availability, and stigma [124-126]. Collectively, these findings suggest that telehealth represents a promising approach to improving access to mental health care for women while also highlighting important areas where the evidence base requires further strengthening.

### Comparison With Prior Work

The present review adds to a growing body of evidence supporting digitally delivered psychological interventions for common mental health conditions. Previous systematic reviews and meta-analyses have consistently reported beneficial effects of telehealth, telepsychiatry, internet-delivered CBT, and other digital mental health interventions for depression and anxiety across diverse populations and health care settings [126-133]. However, most of this evidence has been derived from mixed-sex samples, with limited attention given to whether these findings apply equally to women. By focusing on studies with female-only and predominantly female populations, the present review extends the existing literature and addresses an important evidence gap regarding the effectiveness and acceptability of THIs for women’s mental health.

The findings are broadly consistent with previous evidence syntheses demonstrating that THIs can achieve improvements in depression and anxiety and, in some cases, outcomes comparable to those observed with traditional face-to-face approaches [126,130-133]. Our findings reinforce this growing evidence base and suggest that the benefits associated with THIs remain evident when analyses are restricted to women. This observation is particularly important given that women experience a disproportionate burden of depression, anxiety, and stress-related disorders globally [12,134] yet have rarely been examined as a distinct population in previous meta-analytic research.

The present review also extends previous work by synthesizing evidence across a broad range of women-centered populations, including perinatal women, women living with chronic health conditions, health care workers, caregivers, university students, and women experiencing occupational stress. Previous reviews have often focused on specific diagnostic groups or intervention types [41,42,135], whereas the present review provides a broader perspective on how THIs may function across different stages of the course of life and across varying social and health contexts. The consistency of positive findings across these diverse populations suggests that telehealth may offer a flexible approach capable of addressing a wide range of mental health needs among women.

The subgroup analyses further suggest that benefits of THIs extend beyond comparison with inactive control conditions. Improvements were observed relative to both waitlist control and standard care as comparators for depression and anxiety, indicating that these observed effects are unlikely to be explained solely by the passage of time or the absence of support. At the same time, standard care was not consistently defined across studies and ranged from routine primary care and pharmacological management to low-intensity psychological support [126]. Consequently, the present review does not demonstrate superiority over face-to-face psychotherapy but rather indicates that THIs can provide clinically meaningful benefits within routine models of care.

An additional contribution of this review lies in its synthesis of acceptability outcomes. Previous reviews have often prioritized symptom reduction while giving less attention to user experience and engagement [41-43]. Consistent with broader telehealth literature, participants in studies that reported acceptability or satisfaction outcomes generally reported positive experiences, frequently citing convenience, flexibility, privacy, and reduced stigma as important advantages [126,136,137]. These findings support the growing recognition that successful implementation depends not only on effectiveness but also on whether interventions are acceptable and feasible within the realities of patients’ daily lives.

Altogether, the findings suggest that THIs for women not only are consistent with broader evidence supporting digital mental health care but may also address important gender-related barriers to accessing conventional services. As health systems continue to integrate digital approaches into routine care, understanding how telehealth performs in populations historically underrepresented in evidence syntheses becomes increasingly important.

### Heterogeneity and Methodological Consistency With Prior Reviews

Substantial heterogeneity was observed across the included studies, as previously reported in meta-analyses of digital mental health interventions, telehealth services, and internet-delivered psychological therapies [61,129-131]. Although heterogeneity is often viewed as a limitation, its presence needs to be contextualized in a relatively new and still developing field characterized by diversity in intervention content, delivery modalities, comparator conditions, and participant populations [138,139]. The studies in this review included women across different life stages and circumstances, including perinatal populations, women living with chronic illnesses, health care professionals, caregivers, university students, and women experiencing work-related stress [140,141]. Interventions varied in therapeutic approach, intensity, duration, degree of therapist involvement, technological platform, and delivery mode. Comparator conditions also differed, ranging from waitlist control and treatment as usual to routine primary care and low-intensity psychological support. Such diversity likely contributes to statistical heterogeneity and complicates direct comparisons across studies.

Notably, the heterogeneity observed in this review shows the same patterns reported in previous evidence syntheses of digital mental health interventions [129,130]. The earlier reviews reported a similar finding that overall benefits are often accompanied by substantial between-study variability, which reflects the complex and context-dependent nature of psychological interventions. Consequently, pooled estimates should be interpreted as average effects across diverse settings instead of as effects that can be assumed to occur uniformly across all populations. The PIs observed across outcomes provide additional context for the findings. These PIs reflect the range in which the effect of future studies is expected to fall, whereas CIs describe the precision of the pooled estimate [61]. The wide PIs observed in this review suggest that intervention effectiveness may vary considerably. This finding reinforces the importance of moving beyond questions of whether telehealth works toward a more nuanced understanding of what works, for whom, and under what circumstances.

The measured uncertainty was also contributed by methodological variability. Outcome assessment methods differed across studies, most of the studies relied on self-reported outcome measures rather than clinician-administered diagnostic assessments. Things got further complicated by variations in follow-up duration, participant recruitment, intervention adherence, and reporting quality further complicated synthesis. These challenges are common within digital mental health research and have been identified in previous reviews as barriers to developing more precise estimates of effectiveness [129,130].

Future research should focus more on greater standardization in intervention reporting, outcome assessment, and implementation reporting. More consistent reporting standards would allow us to facilitate more robust moderator analyses and improve understanding of the mechanisms through which THIs achieve benefit. Such efforts are especially important for women-focused research, where evidence remains comparatively fragmented and where understanding differential responses across life stages and social contexts may have important implications for intervention design and implementation.

### Women’s Mental Health: A Neglected Evidence Base

Despite women experiencing a disproportionate burden of depression, anxiety, and stress-related disorders globally [12,134], sex- and gender-specific evidence remains limited within the digital mental health literature [142-145]. Most large-scale reviews and meta-analyses of telehealth and digitally delivered psychological interventions have focused on mixed-sex populations, with relatively little attention given to whether intervention effects differ according to sex or gender [129,130,146]. Consequently, researchers have often been required to extrapolate findings from mixed-sex samples when considering the potential role of THIs for women.

This systematic review and meta-analysis helps address this gap by synthesizing evidence drawn from female-only and predominantly female populations. In doing so, it provides a more direct assessment of telehealth effectiveness and acceptability among women and highlights the importance of considering sex- and gender-related factors when evaluating digital mental health interventions. Women are frequently included in mental health research, but they are less commonly the focus of analyses specifically designed to understand how different factors influence intervention engagement and outcomes.

One notable observation was that tailored interventions designed for specific female populations, particularly perinatal women and caregivers, reported favorable effectiveness and acceptability outcomes. Suggesting that tailored interventions may enhance engagement and outcomes even though the review was not designed to formally compare tailored and nontailored interventions. This interpretation is consistent with broader literature indicating that population-specific adaptation can improve acceptability, uptake, and implementation success, particularly during major life transitions such as pregnancy, childbirth, and early motherhood [147-149].

The findings also highlight the importance of understanding mental health within the broader social context of women’s lives. Women frequently navigate multiple caregiving, occupational, and domestic responsibilities that may create barriers to accessing traditional face-to-face services [125,150,151]. THIs may help address some of these barriers, which may be relevant for women living in rural settings, women with mobility limitations, those caring for children or family members, and women balancing employment with caregiving responsibilities [152-161].

At the same time, telehealth is not equally accessible for everyone. Digital access, technological literacy, socioeconomic circumstances, and cultural factors all influence the extent to which women can benefit from digital mental health services [124,138,162]. These barriers may reduce engagement and risk widening existing health inequalities for women. Future intervention development should therefore consider not only effectiveness but also accessibility, inclusiveness, and implementation equity.

The implications of these observations extend beyond intervention effectiveness alone. Instead of assuming that a single intervention model will meet the needs of all users, developers and policymakers may need to consider how contextual barriers influence engagement with mental health services. Future research should evaluate these factors and incorporate sex and gender considerations into study design from the outset rather than treating them solely as postintervention subgroup variables [161,163]. Continued investment in women-focused digital mental health research will be critical to developing more targeted, equitable, and effective interventions.

### Strengths and Limitations

This review has several strengths. First, to the best of our knowledge, this is the first systematic review and meta-analysis conducted since the COVID-19 pandemic to specifically examine the effectiveness and acceptability of telehealth-based mental health interventions among women. By focusing on female-only and predominantly female populations, the review addresses an important gap in the literature and provides evidence relevant to a population that experienced disproportionate psychological and social distress during the pandemic period [164-166]. Second, this review examined both intervention effectiveness and user satisfaction outcomes, providing a more comprehensive understanding of the clinical value and real-world applicability of THIs. The use of meta-analysis and subgroup analyses provided additional insights into the conditions under which THIs may be most beneficial. Methodologically, the review was conducted rigorously in accordance with PRISMA guidelines and used established tools to assess risk of bias and certainty of evidence.

Several limitations should also be considered. First, substantial heterogeneity was observed across studies. Included populations varied considerably; pregnant women, women with cancer, women with postpartum depression, female students, health care workers, and community-based samples were all included in the analysis. THIs also varied substantially in delivery format, content, duration, intensity, and guidance. This diversity contributed to statistical heterogeneity and limited the feasibility of conducting more detailed subgroup analyses. Additionally, most studies relied on self-reported outcome measures rather than clinician-administered assessments, which may have introduced measurement bias. Variability in outcome measures, follow-up periods, and reporting quality further contributed to uncertainty in the pooled estimates.

The COVID-19 context should also be considered when interpreting these findings. Many of the included studies were conducted during or shortly after a period of rapid telehealth expansion, when remote care often shifted from being an alternative model of service delivery to a primary means of accessing psychological support. Consequently, some observed benefits may partly reflect the continuity of care provided during a period of widespread disruption to traditional health care services rather than the effectiveness of intervention content alone. The pandemic context may also have had other relevance for women, including increased caregiving responsibilities, employment disruption, and social isolation, and heightened psychological distress could have influenced both mental health needs and engagement with telehealth services. At the same time, the rapid implementation of telehealth exposed challenges related to digital literacy, technology access, privacy concerns, and implementation equity. These issues may have affected women experiencing socioeconomic disadvantages as well as limited access to technology or residence in rural and underserved settings.

Finally, despite a comprehensive search strategy, relatively few high-quality RCTs focused specifically on women. The restriction to peer-reviewed studies published in English may also have resulted in the exclusion of relevant evidence from other languages. Moderate to high heterogeneity in average effects (CIs), wide distribution of effects across different settings (PIs), and the identified risk of bias resulted in a moderate certainty of evidence in GRADE assessments. Accordingly, pooled estimates should be interpreted as average effects that may not be uniform across real-world contexts.

### Implications for Policy and Practice

The findings of this systematic review and meta-analysis underscore the growing relevance of THIs in addressing depression, anxiety, and stress among women. Given the observed clinical benefits, high acceptability, and strong user satisfaction reported across the included studies, THIs should be considered a viable component of mental health service delivery, particularly for women with mild to moderate symptoms.

Existing evidence has primarily evaluated telehealth in outpatient and community settings [167], with comparatively less attention given to severe or acute conditions. Therefore, the suitability and effectiveness of THIs in these contexts require further investigation.

These findings support integrating digital platforms into mental health care, particularly for underserved and remote populations. Telehealth may enhance access, flexibility, and responsiveness while addressing structural, social, and logistical barriers to care. Although telehealth should not replace face-to-face care universally, it can serve as an effective complement or alternative in appropriate contexts. More broadly, the COVID-19 pandemic accelerated telemedicine adoption worldwide and highlighted its value in maintaining continuity of care during disruption [168]. Although in-person services have largely resumed, telemedicine remains important in contemporary health care delivery. Its advantages may be particularly relevant for individuals in remote areas, those with mobility limitations, and populations facing barriers to conventional services.

Finally, this review contributes by synthesizing evidence on the effectiveness and acceptability of telemental health interventions in female-only and female-predominant populations. By integrating quantitative and narrative evidence, this review informs future clinical practice, service development, and policy decision-making. Nevertheless, important questions remain regarding equity in telemedicine adoption. While telehealth is increasingly embedded in technologically advanced health systems, infrastructure, digital literacy, and access barriers may constrain its sustainability elsewhere [124]. Future policy and implementation efforts should ensure that telehealth expansion reduces, rather than reinforces, existing health inequalities.

### Conclusions

Evidence from this review suggests that telehealth-based mental health services are associated with reductions in depression and anxiety among women, with a smaller and less certain effect for stress. Moderate to high heterogeneity in average effects (CIs), wide distribution of effects across different settings (PIs), and the identified risk of bias resulted in a moderate certainty of evidence in GRADE assessments. The observed diversity in study designs, intervention delivery, and longitudinal reporting likely contributed to heterogeneity, suggesting the need for greater standardization in assessing and reporting online mental health interventions. There is also a need for regionally tailored research that captures the socioeconomic, cultural, and technological specificities of each context. This would guide future research, support more effective interventions, and clarify inequalities in mental health care across populations. Overall, the findings from the present study suggest that telehealth is a valuable tool to improve access when traditional care settings are unavailable or difficult to access, while further high-quality women-focused research is needed to clarify the conditions under which benefits are most reliably achieved.

## Supplementary material

10.2196/85494 Multimedia Appendix 1 Search strategy.

10.2196/85494 Multimedia Appendix 2 Characteristics of studies included in the systematic review and meta-analysis.

10.2196/85494 Multimedia Appendix 3 Tables depicting results of risk of bias and Grading of Recommendations, Assessment, Development and Evaluation (GRADE) assessments.

10.2196/85494 Multimedia Appendix 4 Figures depicting the results of publication bias analysis, sensitivity analyses, and subgroup analyses.

10.2196/85494 Checklist 1 PRISMA 2020 checklist and PRISMA 2020 checklist for abstracts.

## Abbreviations

BAI: Beck Anxiety Inventory
BDI: Beck Depression Inventory
CBT: cognitive behavioral therapy
CESD-R: Center for Epidemiologic Studies Depression Scale—Revised
CINAHL: Cumulative Index to Nursing and Allied Health Literature
CSQ-8: Client Satisfaction Questionnaire
CSS: Client Satisfaction Scale
DASS: Depression Anxiety Stress Scales
DASS-S: stress subscale of the Depression Anxiety Stress Scales
DTS: Davidson Trauma Scale
EPDS: Edinburgh Postnatal Depression Scale
GAD-7: 7-item Generalized Anxiety Disorder Scale
GRADE: Grading of Recommendations, Assessment, Development and Evaluation
HADS: Hospital Anxiety and Depression Scale
IECBT: internet-based emotion-focused cognitive behavioral therapy
MADRS: Montgomery-Åsberg Depression Rating Scale
NICE: National Institute for Health and Care Excellence
PHQ: Patient Health Questionnaire
PICOS: population, intervention, comparison, outcome, and study design
PPQ: Patient Participation Questionnaire
PRISMA: Preferred Reporting Items for Systematic Reviews and Meta-Analyses
PRISMA-S: Preferred Reporting Items for Systematic Reviews and Meta-Analyses Literature Search extension
PROMIS: Patient-Reported Outcomes Measurement Information System
PROMIS-Anx6a: Patient-Reported Outcomes Measurement Information System Anxiety Short Form 6a
PROMIS-D-6a: Patient-Reported Outcomes Measurement Information System Depression Short Form 6a
PSS: Perceived Stress Scale
PSS-14: 14-item version of the Perceived Stress Scale
PSS-4: 4-item version of the Perceived Stress Scale
RCT: randomized controlled trial
ROBINS-I: Risk of Bias Assessment in Non-Randomized Studies of Interventions
SCL-90-R: Symptom Checklist-90-Revised
VAS: Visual Analogue Scale
WHO: World Health Organization
