# Supplementary material for: Effectiveness of Telehealth Care Interventions for Women’s Mental Health During and After the COVID-19 Pandemic: Systematic Review and Meta-Analysis
Source: J Med Internet Res. 2026 Sep 21;28:e85494. doi: 10.2196/85494 (PMC13592667; doi:10.2196/85494)
Supplement: Supplementary file 1 [file jmir-v28-e85494-s001.docx]

**Multimedia Appendix 1. Search strategy**

|  | **Medline (Ovid MEDLINE® Epub Ahead of Print, In-Process & Other Non-Indexed Citations, Ovid MEDLINE® Daily and Ovid MEDLINE®) 1946 to present** |
| --- | --- |
| 1 | SARS-CoV-2/ or COVID-19/ |
| 2 | (corona* adj1 (virus* or viral*)).ti,ab,kw,kf. |
| 3 | (CoV not (Coefficien* or "co-efficien*" or covalent* or Covington* or covariant* or covarianc* or "cut-off value*" or "cutoff value*" or "cut-off volume*" or "cutoff volume*" or "combined optimi?ation value*" or "central vessel trunk*" or CoVR or CoVS)).ti,ab,kw,kf. |
| 4 | (coronavirus* or 2019nCoV* or 19nCoV* or "2019 novel*" or Ncov* or "n-cov" or "SARSCoV-2*" or "SARSCoV-2*" or SARSCoV2* or "SARS-CoV2*" or "severe acute respiratory syndrome*" or COVID*2).ti,ab,kw,kf. |
| 5 | 1 or 2 or 3 or 4 |
| 6 | Anxiety/ or exp Anxiety Disorders/ |
| 7 | Depression/ |
| 8 | mental disorders/ or mood disorders/ or exp depressive disorder/ |
| 9 | Mental Health/ |
| 10 | mental fatigue/ or exp stress, psychological/ |
| 11 | (depress* or anxiety or anxious or phobia? or phobi? or obsessive compulsive disorder? or panic or mood? or ((psychological* or mental* or emotional*) adj3 (stress* or distress* or disorder?))).ti,ab,kf. |
| 12 | (mental* adj2 (ill* or health or disease)).ti,ab,kf. |
| 13 | 6 or 7 or 8 or 9 or 10 or 11 or 12 |
| 14 | 5 and 13 |
| 15 | exp COVID-19/px [Psychology] |
| 16 | 14 or 15 |
| 17 | behavior therapy/ or exp cognitive behavioral therapy/ |
| 18 | counseling/ or distance counseling/ |
| 19 | psychotherapy/ or psychosocial intervention/ or psychotherapy, brief/ |
| 20 | Anxiety/pc, th or exp Anxiety Disorders/pc, th or Depression/pc, th or mental disorders/pc, th or mood disorders/pc, th or exp depressive disorder/pc, th or mental fatigue/pc, th or exp stress, psychological/pc, th |
| 21 | (psychotherap* or ((cognitive or behavio* or psycho) adj therap*) or ((psycho* or social* or peer) adj support) or counsel?ing or mindfulness or meditation or wellness).ti,ab,kf. |
| 22 | 17 or 18 or 19 or 20 or 21 |
| 23 | Telemedicine/ |
| 24 | Mobile Applications/ |
| 25 | exp Internet/ |
| 26 | exp Cell Phone/ |
| 27 | exp Computers, Handheld/ |
| 28 | Medical Informatics Applications/ |
| 29 | Therapy, Computer-Assisted/ |
| 30 | (telemed* or tele-med* or telehealth* or tele-health or telemental* or tele-mental*).ti,ab. |
| 31 | (app or apps).ti,ab. |
| 32 | (online or web or internet or digital*).ti. |
| 33 | ((online or web or internet or digital*) adj3 (based or application* or intervention* or program* or therap*)).ab. |
| 34 | (phone* or telephone* or smartphone* or cellphone* or smartwatch*).ti. |
| 35 | ((phone* or telephone* or smartphone* or cellphone* or smartwatch*) adj3 (based or application* or intervention* or program* or therap*)).ab. |
| 36 | (mobile health or mhealth or m-health or ehealth or e-health or emental or e-mental).ti. |
| 37 | ((mobile health or mhealth or m-health or ehealth or e-health or emental or e-mental) adj3 (based or application* or intervention* or program* or therap*)).ab. |
| 38 | (mobile* adj3 (based or application* or intervention* or device* or technolog*)).ti,ab. |
| 39 | 23 or 24 or 25 or 26 or 27 or 28 or 29 or 30 or 31 or 32 or 33 or 34 or 35 or 36 or 37 or 38 |
| 40 | 22 and 39 |
| 41 | (telemental or tele-mental or telepsych* or tele-psych*).ti,ab,kf. |
| 42 | 40 or 41 |
| 43 | randomized controlled trial.pt. |
| 44 | controlled clinical trial.pt. |
| 45 | multicenter study.pt. |
| 46 | pragmatic clinical trial.pt. |
| 47 | (randomis* or randomiz* or randomly).ti,ab. |
| 48 | groups.ab. |
| 49 | (trial or multicenter or multi center or multicentre or multi centre).ti. |
| 50 | (intervention? or effect? or impact? or controlled or control group? or (before adj5 after) or (pre adj5 post) or ((pretest or pre test) and (posttest or post test)) or quasiexperiment* or quasi experiment* or pseudo experiment* or pseudoexperiment* or evaluat* or time series or time point? or repeated measur*).ti,ab. |
| 51 | non-randomized controlled trials as topic/ |
| 52 | interrupted time series analysis/ |
| 53 | controlled before-after studies/ |
| 54 | or/43-53 |
| 55 | exp animals/ not humans/ |
| 56 | 54 not 55 |
| 57 | 16 and 42 and 56 |

|  | **Embase 1974 to present** |
| --- | --- |
| 1 | exp severe acute respiratory syndrome coronavirus 2/ or exp coronavirus disease 2019/ or experimental coronavirus disease 2019/ |
| 2 | (corona* adj1 (virus* or viral*)).ti,ab,kw,kf. |
| 3 | (CoV not (Coefficien* or "co-efficien*" or covalent* or Covington* or covariant* or covarianc* or "cut-off value*" or "cutoff value*" or "cut-off volume*" or "cutoff volume*" or "combined optimi?ation value*" or "central vessel trunk*" or CoVR or CoVS)).ti,ab,kw,kf. |
| 4 | (coronavirus* or 2019nCoV* or 19nCoV* or "2019 novel*" or Ncov* or "n-cov" or "SARSCoV-2*" or "SARSCoV-2*" or SARSCoV2* or "SARS-CoV2*" or "severe acute respiratory syndrome*" or COVID*2).ti,ab,kw,kf. |
| 5 | 1 or 2 or 3 or 4 |
| 6 | Anxiety/ or exp Anxiety Disorders/ |
| 7 | mood disorder/ or exp depression/ |
| 8 | psychological well-being/ |
| 9 | Mental Health/ or Mental Disease/ |
| 10 | mental fatigue/ |
| 11 | (depress* or anxiety or anxious or phobia? or phobi? or obsessive compulsive disorder? or panic or mood? or ((psychological* or mental* or emotional*) adj3 (stress* or distress* or disorder?))).ti,ab,kf. |
| 12 | (mental* adj2 (ill* or health or disease)).ti,ab,kf. |
| 13 | 6 or 7 or 8 or 9 or 10 or 11 or 12 |
| 14 | exp cognitive behavioral therapy/ or behavior therapy/ or cognitive therapy/ |
| 15 | exp counseling/ |
| 16 | psychotherapy/ or exp mindfulness/ or short term psychotherapy/ |
| 17 | Anxiety/pc, th or exp Anxiety Disorders/pc, th or exp Depression/pc, th or mental disease/pc, th or mental health/pc, th or mental fatigue/pc, th |
| 18 | (psychotherap* or ((cognitive or behavio* or psycho) adj therap*) or ((psycho* or social* or peer) adj support) or counsel?ing or mindfulness or meditation or wellness).ti,ab,kf. |
| 19 | 14 or 15 or 16 or 17 or 18 |
| 20 | Mobile Application/ |
| 21 | exp Internet/ |
| 22 | exp Mobile Phone/ |
| 23 | text messaging/ |
| 24 | personal digital assistant/ |
| 25 | computer assisted therapy/ |
| 26 | (telemed* or tele-med* or telehealth* or tele-health).ti,ab. |
| 27 | (app or apps).ti,ab. |
| 28 | (online or web or internet or digital*).ti. |
| 29 | ((online or web or internet or digital*) adj3 (based or application* or intervention* or program* or therap*)).ab. |
| 30 | (phone* or telephone* or smartphone* or cellphone* or smartwatch*).ti. |
| 31 | ((phone* or telephone* or smartphone* or cellphone* or smartwatch*) adj3 (based or application* or intervention* or program* or therap*)).ab. |
| 32 | (mobile health or mhealth or m-health or ehealth or e-health or emental or e-mental).ti. |
| 33 | ((mobile health or mhealth or m-health or ehealth or e-health or emental or e-mental) adj3 (based or application* or intervention* or program* or therap*)).ab. |
| 34 | (mobile* adj3 (based or application* or intervention* or device* or technolog*)).ti,ab. |
| 35 | 20 or 21 or 22 or 23 or 24 or 25 or 26 or 27 or 28 or 29 or 30 or 31 or 32 or 33 or 34 |
| 36 | 19 and 35 |
| 37 | (telemental or tele-mental or telepsych* or tele-psych*).ti,ab,kf. |
| 38 | telepsychotherapy/ |
| 39 | 36 or 37 or 38 |
| 40 | exp randomized controlled trial/ |
| 41 | Controlled clinical trial/ |
| 42 | random$.ti,ab. |
| 43 | randomization/ |
| 44 | intermethod comparison/ |
| 45 | placebo.ti,ab. |
| 46 | (compare or compared or comparison).ti. |
| 47 | ((evaluated or evaluate or evaluating or assessed or assess) and (compare or compared or comparing or comparison)).ab. |
| 48 | (open adj label).ti,ab. |
| 49 | ((double or single or doubly or singly) adj (blind or blinded or blindly)).ti,ab. |
| 50 | double blind procedure/ |
| 51 | parallel group$1.ti,ab. |
| 52 | (crossover or cross over).ti,ab. |
| 53 | ((assign$ or match or matched or allocation) adj5 (alternate or group$1 or intervention$1 or patient$1 or subject$1 or participant$1)).ti,ab. |
| 54 | (assigned or allocated).ti,ab. |
| 55 | (controlled adj7 (study or design or trial)).ti,ab. |
| 56 | (volunteer or volunteers).ti,ab. |
| 57 | human experiment/ |
| 58 | (trial or multicenter or multi center or multicentre or multi centre).ti. |
| 59 | time series analysis/ |
| 60 | intervention study/ |
| 61 | (intervention? or effect? or impact? or controlled or control group? or (before adj5 after) or (pre adj5 post) or ((pretest or pre test) and (posttest or post test)) or quasiexperiment* or quasi experiment* or pseudo experiment* or pseudoexperiment* or evaluat* or time series or time point? or repeated measur*).ti,ab. |
| 62 | or/40-61 |
| 63 | (random$ adj sampl$ adj7 ("cross section$" or questionnaire$1 or survey$ or database$1)).ti,ab. not (comparative study/ or controlled study/ or randomi?ed controlled.ti,ab. or randomly assigned.ti,ab.) |
| 64 | Cross-sectional study/ not (exp randomized controlled trial/ or controlled clinical study/ or controlled study/ or randomi?ed controlled.ti,ab. or control group$1.ti,ab.) |
| 65 | (((case adj control$) and random$) not randomi?ed controlled).ti,ab. |
| 66 | Systematic review.ti,ab. not (trial or study).ti. |
| 67 | random field$.ti,ab. |
| 68 | (random cluster adj3 sampl$).ti,ab. |
| 69 | (review.ab. and review.pt.) not trial.ti. |
| 70 | we searched.ab. and (review.ti. or review.pt.) |
| 71 | update review.ab. |
| 72 | (databases adj4 searched).ab. |
| 73 | (rat or rats or mouse or mice or swine or porcine or murine or sheep or lambs or pigs or piglets or rabbit or rabbits or cat or cats or dog or dogs or cattle or bovine or monkey or monkeys or trout or marmoset$1).ti. and animal experiment/ |
| 74 | Animal experiment/ not (human experiment/ or human/) |
| 75 | or/63-74 |
| 76 | 62 not 75 |
| 77 | 5 and 13 and 39 and 76 |

|  | **PsycINFO 1806 to present** |
| --- | --- |
| 1 | exp covid-19/ |
| 2 | (corona* adj1 (virus* or viral*)).ti,ab,id. |
| 3 | (CoV not (Coefficien* or "co-efficien*" or covalent* or Covington* or covariant* or covarianc* or "cut-off value*" or "cutoff value*" or "cut-off volume*" or "cutoff volume*" or "combined optimi?ation value*" or "central vessel trunk*" or CoVR or CoVS)).ti,ab,id. |
| 4 | (coronavirus* or 2019nCoV* or 19nCoV* or "2019 novel*" or Ncov* or "n-cov" or "SARSCoV-2*" or "SARSCoV-2*" or SARSCoV2* or "SARS-CoV2*" or "severe acute respiratory syndrome*" or COVID*2).ti,ab,id. |
| 5 | 1 or 2 or 3 or 4 |
| 6 | anxiety/ or health anxiety/ or exp anxiety disorders/ |
| 7 | affective disorders/ or exp major depression/ |
| 8 | mental disorders/ |
| 9 | Mental Health/ |
| 10 | psychological stress/ |
| 11 | (depress* or anxiety or anxious or phobia? or phobi? or obsessive compulsive disorder? or panic or mood? or ((psychological* or mental* or emotional*) adj3 (stress* or distress* or disorder?))).ti,ab,id. |
| 12 | (mental* adj2 (ill* or health or disease)).ti,ab,id. |
| 13 | 6 or 7 or 8 or 9 or 10 or 11 or 12 |
| 14 | exp cognitive behavior therapy/ or behavior therapy/ |
| 15 | counseling/ or microcounseling/ |
| 16 | psychotherapy/ or exp brief psychotherapy/ or psychotherapeutic counseling/ |
| 17 | exp Mindfulness-Based Cognitive Therapy/ or Mindfulness/ |
| 18 | exp psychosocial interventions/ |
| 19 | (psychotherap* or ((cognitive or behavio* or psycho) adj therap*) or ((psycho* or social* or peer) adj support) or counsel?ing or mindfulness or meditation or wellness).ti,ab,id. |
| 20 | 14 or 15 or 16 or 17 or 18 or 19 |
| 21 | telemedicine/ or online therapy/ or teleconsultation/ |
| 22 | Mobile Applications/ |
| 23 | Internet/ |
| 24 | mobile devices/ or exp mobile phones/ or tablet computers/ |
| 25 | digital interventions/ |
| 26 | mobile health/ |
| 27 | computer assisted therapy/ |
| 28 | (telemed* or tele-med* or telehealth* or tele-health or telemental* or tele-mental*).ti,ab. |
| 29 | (app or apps).ti,ab. |
| 30 | (online or web or internet or digital*).ti. |
| 31 | ((online or web or internet or digital*) adj3 (based or application* or intervention* or program* or therap*)).ab. |
| 32 | (phone* or telephone* or smartphone* or cellphone* or smartwatch*).ti. |
| 33 | ((phone* or telephone* or smartphone* or cellphone* or smartwatch*) adj3 (based or application* or intervention* or program* or therap*)).ab. |
| 34 | (mobile health or mhealth or m-health or ehealth or e-health or emental or e-mental).ti. |
| 35 | ((mobile health or mhealth or m-health or ehealth or e-health or emental or e-mental) adj3 (based or application* or intervention* or program* or therap*)).ab. |
| 36 | (mobile* adj3 (based or application* or intervention* or device* or technolog*)).ti,ab. |
| 37 | 21 or 22 or 23 or 24 or 25 or 26 or 27 or 28 or 29 or 30 or 31 or 32 or 33 or 34 or 35 or 36 |
| 38 | 20 and 37 |
| 39 | telepsychiatry/ or telepsychology/ |
| 40 | (telemental or tele-mental or telepsych* or tele-psych*).ti,ab,id. |
| 41 | 38 or 39 or 40 |
| 42 | random*.ti,ab,hw,id. |
| 43 | trial*.ti,ab,hw,id. |
| 44 | controlled stud*.ti,ab,hw,id. |
| 45 | placebo*.ti,ab,hw,id. |
| 46 | ((singl* or doubl* or trebl* or tripl*) and (blind* or mask*)).ti,ab,hw,id. |
| 47 | (cross over or crossover or factorial* or latin square).ti,ab,hw,id. |
| 48 | (assign* or allocat* or volunteer*).ti,ab,hw,id. |
| 49 | treatment effectiveness evaluation/ |
| 50 | mental health program evaluation/ |
| 51 | exp experimental design/ |
| 52 | (clinical trial or treatment outcome).md. |
| 53 | repeated measures/ |
| 54 | (intervention? or effect? or impact? or controlled or control group? or (before adj5 after) or (pre adj5 post) or ((pretest or pre test) and (posttest or post test)) or quasiexperiment* or quasi experiment* or pseudo experiment* or pseudoexperiment* or evaluat* or time series or time point? or repeated measur*).ti,ab. |
| 55 | (multicenter or multi center or multicentre or multi centre).ti. |
| 56 | 42 or 43 or 44 or 45 or 46 or 47 or 48 or 49 or 50 or 51 or 52 or 53 or 54 or 55 |
| 57 | (animal not human).po. |
| 58 | 56 not 57 |
| 59 | 5 and 13 and 41 and 58 |

| S# | **CINAHL** |
| --- | --- |
| S20 | S18 AND S15 |
| S19 | S18 AND S15 |
| S18 | S16 NOT S17 |
| S17 | (MH animals+ OR MH (animal studies) OR TI (animal model*)) NOT MH (human) |
| S16 | (MH randomized controlled trials OR MH double-blind studies OR MH single-blind studies OR MH random assignment OR MH pretest-posttest design OR MH cluster sample OR TI (randomised OR randomized) OR AB (random*) OR TI (trial) OR (MH (sample size) AND AB (assigned OR allocated OR control)) OR MH (placebos) OR PT (randomized controlled trial) OR AB (control W5 group) OR MH (crossover design) OR MH (comparative studies) OR AB (cluster W3 RCT)) OR ((MH "Controlled Before-After Studies") OR (MH "Interrupted Time Series Analysis") OR (MH "Nonrandomized Trials") OR (MH "Pretest-Posttest Design+") OR (MH "Quasi-Experimental Studies+") OR (MH "Repeated Measures")) OR (( ((TI multicenter) OR (TI "multi center") OR (TI multicentre) OR (TI "multi centre")) ) OR ( ((TI intervention# OR AB intervention#) OR (TI effect# OR AB effect#) OR (TI impact# OR AB impact#) OR (TI controlled OR AB controlled) OR (TI "control group#" OR AB "control group#") OR ((TI before OR AB before) N5 (TI after OR AB after)) OR ((TI pre OR AB pre) N5 (TI post OR AB post)) OR (((TI pretest OR AB pretest) OR (TI "pre test" OR AB "pre test")) AND ((TI posttest OR AB posttest) OR (TI "post test" OR AB "post test"))) OR (TI quasiexperiment* OR AB quasiexperiment*) OR (TI "quasi experiment*" OR AB "quasi experiment*") OR (TI "pseudo experiment*" OR AB "pseudo experiment*") OR (TI pseudoexperiment* OR AB pseudoexperiment*) OR (TI evaluat* OR AB evaluat*) OR (TI "time series" OR AB "time series") OR (TI "time point#" OR AB "time point#") OR (TI "repeated measur*" OR AB "repeated measur*")) )) |
| S15 | S14 OR S12 |
| S14 | S13 AND S8 |
| S13 | (MH "Telepsychiatry") OR (TI telemental* OR AB telemental* OR TI tele-mental* OR AB tele-mental* OR TI telepsych* OR AB telepsyc* OR TI tele-psych* OR AB tele-psych*) |
| S12 | S11 AND S10 AND S9 AND S8 |
| S11 | ((MH "Mobile Applications") OR (MH "Internet+") OR (MH "Instant Messaging") OR (MH "Telehealth") OR (MH "Telemedicine") OR (MH "Cellular Phone+") OR (MH "Text Messaging+") OR (MH "Computers, Hand-Held+") OR (MH "Therapy, Computer Assisted")) OR (((TI app OR AB app) OR (TI apps OR AB apps)) OR ((TI online) OR (TI web) OR (TI internet) OR (TI digital*)) OR (((AB online) OR (AB web) OR (AB internet) OR (AB digital*)) N3 ((AB based) OR (AB application*) OR (AB intervention*) OR (AB program*) OR (AB therap*))) OR ((TI phone*) OR (TI telephone*) OR (TI smartphone*) OR (TI cellphone*) OR (TI smartwatch*)) OR (((AB phone*) OR (AB telephone*) OR (AB smartphone*) OR (AB cellphone*) OR (AB smartwatch*)) N3 ((AB based) OR (AB application*) OR (AB intervention*) OR (AB program*) OR (AB therap*))) OR ((TI "mobile health") OR (TI mhealth) OR (TI m-health) OR (TI ehealth) OR (TI e-health) OR (TI emental) OR (TI e-mental)) OR (((AB "mobile health") OR (AB mhealth) OR (AB m-health) OR (AB ehealth) OR (AB e-health) OR (AB emental) OR (AB e-mental)) N3 ((AB based) OR (AB application*) OR (AB intervention*) OR (AB program*) OR (AB therap*))) or ((TI mobile* OR AB mobile*) N3 ((TI based OR AB based) OR (TI application* OR AB application*) OR (TI intervention* OR AB intervention*) OR (TI device* OR AB device*) OR (TI technolog* OR AB technolog*)))) |
| S10 | ((MH "Motivational Interviewing") OR (MH "Peer Counseling") OR (MH "Psychotherapy") OR (MH "Psychotherapy, Brief") OR (MH "Support, Psychosocial") OR (MH "Behavior Therapy") OR (MH "Cognitive Therapy") OR (MH "Mindfulness")) OR ((MH "Mental Disorders/PC/TH") OR (MH "Affective Disorders/PC/TH") OR (MH "Depression/PC/TH") OR (MH "Anxiety Disorders+/PC/TH") OR (MH "Mental Fatigue/PC/TH") OR (MH "Psychological Distress/PC") OR (MH "Stress, Psychological/PC/TH")) OR (((TI psychotherap* OR AB psychotherap* OR SU psychotherap*) OR (((TI cognitive OR AB cognitive OR SU cognitive) OR (TI behavio* OR AB behavio* OR SU behavio*) OR (TI psycho OR AB psycho OR SU psycho)) W1 (TI therap* OR AB therap* OR SU therap*)) OR (((TI psycho* OR AB psycho* OR SU psycho*) OR (TI social* OR AB social* OR SU social*) OR (TI peer OR AB peer OR SU peer)) W1 (TI support OR AB support OR SU support)) OR (TI counsel#ing OR AB counsel#ing OR SU counsel#ing) OR (TI mindfulness OR AB mindfulness OR SU mindfulness) OR (TI meditation OR AB meditation OR SU meditation) OR (TI wellness OR AB wellness OR SU wellness))) |
| S9 | ((MH "Depression") OR (MH "Depression, Postpartum") OR (MH "Anxiety Disorders+") OR (MH "Anxiety") OR (MH "Psychological Distress") OR (MH "Mental Fatigue") OR (MH "Stress, Psychological") OR (MH "Mental Health")) OR ((((TI depress* OR AB depress* OR SU depress*) OR (TI anxiety OR AB anxiety OR SU anxiety) OR (TI anxious OR AB anxious OR SU anxious) OR (TI phobia# OR AB phobia# OR SU phobia#) OR (TI phobi# OR AB phobi# OR SU phobi#) OR (TI "obsessive compulsive disorder#" OR AB "obsessive compulsive disorder#" OR SU "obsessive compulsive disorder#") OR (TI panic OR AB panic OR SU panic) OR (TI mood# OR AB mood# OR SU mood#) OR (((TI psychological* OR AB psychological* OR SU psychological*) OR (TI mental* OR AB mental* OR SU mental*) OR (TI emotional* OR AB emotional* OR SU emotional*)) N3 ((TI stress* OR AB stress* OR SU stress*) OR (TI distress* OR AB distress* OR SU distress*) OR (TI disorder# OR AB disorder# OR SU disorder#))))) OR (((TI mental* OR AB mental* OR SU mental*) N2 ((TI ill* OR AB ill* OR SU ill*) OR (TI health OR AB health OR SU health) OR (TI disease OR AB disease OR SU disease))))) |
| S8 | ((MH "COVID-19+") OR (MH "Severe Acute Respiratory Syndrome")) OR (((TI corona* OR AB corona*) N1 ((TI virus* OR AB virus*) OR (TI viral* OR AB viral*))) OR ((TI CoV OR AB CoV) NOT ((TI Coefficien* OR AB Coefficien*) OR (TI co-efficien* OR AB co-efficien*) OR (TI covalent* OR AB covalent*) OR (TI Covington* OR AB Covington*) OR (TI covariant* OR AB covariant*) OR (TI covarianc* OR AB covarianc*) OR (TI "cut-off value*" OR AB "cut-off value*") OR (TI "cutoff value*" OR AB "cutoff value*") OR (TI "cut-off volume*" OR AB "cut-off volume*") OR (TI "cutoff volume*" OR AB "cutoff volume*") OR (TI "combined optimi#ation value*" OR AB "combined optimi#ation value*") OR (TI "central vessel trunk*" OR AB "central vessel trunk*") OR (TI CoVR OR AB CoVR) OR (TI CoVS OR AB CoVS))) OR ((TI coronavirus* OR AB coronavirus*) OR (TI 2019nCoV* OR AB 2019nCoV*) OR (TI 19nCoV* OR AB 19nCoV*) OR (TI "2019 novel*" OR AB "2019 novel*") OR (TI Ncov* OR AB Ncov*) OR (TI n-cov OR AB n-cov) OR (TI SARSCoV-2* OR AB SARSCoV-2*) OR (TI SARSCoV-2* OR AB SARSCoV-2*) OR (TI SARSCoV2* OR AB SARSCoV2*) OR (TI SARS-CoV2* OR AB SARS-CoV2*) OR (TI "severe acute respiratory syndrome*" OR AB "severe acute respiratory syndrome*") OR (TI COVID*2 OR AB COVID*2))) |

**Cochrane Library**

| ID | | Search |
| --- | --- | --- |
| #1 | [mh COVID-19] | |
| #2 | (corona*:ti,ab NEAR/1 (virus*:ti,ab OR viral*:ti,ab,kw)) OR (CoV:ti,ab NOT (Coefficien*:ti,ab OR co-efficien*:ti,ab OR covalent*:ti,ab OR Covington*:ti,ab OR covariant*:ti,ab OR covarianc*:ti,ab OR ("cut-off" NEXT value*):ti,ab OR ("cutoff" NEXT value*):ti,ab OR ("cut-off" NEXT volume*):ti,ab OR ("cutoff" NEXT volume*):ti,ab OR ("combined" NEXT optimi?ation NEXT value*):ti,ab OR ("central vessel" NEXT trunk*):ti,ab OR CoVR:ti,ab OR CoVS:ti,ab,kw)) OR (coronavirus*:ti,ab OR 2019nCoV*:ti,ab OR 19nCoV*:ti,ab OR ("2019" NEXT novel*):ti,ab OR Ncov*:ti,ab OR n-cov:ti,ab OR SARSCoV-2*:ti,ab OR SARSCoV-2*:ti,ab OR SARSCoV2*:ti,ab OR SARS-CoV2*:ti,ab OR ("severe acute respiratory" NEXT syndrome*):ti,ab OR COVID*2:ti,ab,kw) | |
| #3 | #1 OR #2 | |
| #4 | [mh ^Anxiety] OR [mh "Anxiety Disorders"] OR [mh ^Depression] OR [mh ^"mood disorders"] OR [mh "depressive disorder"] OR [mh ^"Mental Health"] OR [mh "stress, psychological"] OR [mh "mental fatigue"] | |
| #5 | (depress*:ti,ab,kw OR anxiety:ti,ab,kw OR anxious:ti,ab,kw OR phobia?:ti,ab,kw OR phobi?:ti,ab,kw OR ("obsessive compulsive" NEXT disorder?):ti,ab,kw OR panic:ti,ab,kw OR mood?:ti,ab,kw OR ((psychological*:ti,ab,kw OR mental*:ti,ab,kw OR emotional*:ti,ab,kw) NEAR/3 (stress*:ti,ab,kw OR distress*:ti,ab,kw OR disorder?:ti,ab,kw))) OR (mental*:ti,ab,kw NEAR/2 (ill*:ti,ab,kw OR health:ti,ab,kw OR disease:ti,ab,kw)) | |
| #6 | #4 OR #5 | |
| #7 | [mh ^"behavior therapy"] OR [mh "cognitive behavioral therapy"] OR [mh ^counseling] OR [mh ^"distance counseling"] OR [mh ^"psychosocial intervention"] OR [mh ^"psychotherapy, brief"] OR [mh "Anxiety Disorders"/pc,th] OR [mh ^Depression/pc,th] OR [mh ^"mental disorders"/pc,th] OR [mh ^"mood disorders"/pc,th] OR [mh "depressive disorder"/pc,th] OR [mh ^"mental fatigue"/pc,th] OR [mh "stress, psychological"/pc,th] | |
| #8 | (psychotherap*:ti,ab,kw OR ((cognitive:ti,ab,kw OR behavio*:ti,ab,kw OR psycho:ti,ab,kw) NEXT therap*:ti,ab,kw) OR ((psycho*:ti,ab,kw OR social*:ti,ab,kw OR peer:ti,ab,kw) NEXT support:ti,ab,kw) OR counsel?ing:ti,ab,kw OR mindfulness:ti,ab,kw OR meditation:ti,ab,kw OR wellness:ti,ab,kw) | |
| #9 | #7 or #8 | |
| #10 | [mh ^Telemedicine] OR [mh Internet] OR [mh "Cell Phone"] OR [mh "Computers, Handheld"] OR [mh "mobile applications"] | |
| #11 | (telemed*:ti,ab OR tele-med*:ti,ab OR telehealth*:ti,ab OR tele-health:ti,ab OR telemental*:ti,ab OR tele-mental*:ti,ab) OR (app:ti,ab OR apps:ti,ab) OR (online:ti OR web:ti OR internet:ti OR digital*:ti) OR ((online:ab OR web:ab OR internet:ab OR digital*:ab) NEAR/3 (based:ab OR application*:ab OR intervention*:ab OR program*:ab OR therap*:ab)) OR (phone*:ti OR telephone*:ti OR smartphone*:ti OR cellphone*:ti OR smartwatch*:ti) OR ((phone*:ab OR telephone*:ab OR smartphone*:ab OR cellphone*:ab OR smartwatch*:ab) NEAR/3 (based:ab OR application*:ab OR intervention*:ab OR program*:ab OR therap*:ab)) OR ("mobile health":ti OR mhealth:ti OR m-health:ti OR ehealth:ti OR e-health:ti OR emental:ti OR e-mental:ti) OR (("mobile health":ab OR mhealth:ab OR m-health:ab OR ehealth:ab OR e-health:ab OR emental:ab OR e-mental:ab) NEAR/3 (based:ab OR application*:ab OR intervention*:ab OR program*:ab OR therap*:ab)) OR (mobile*:ti,ab NEAR/3 (based:ti,ab OR application*:ti,ab OR intervention*:ti,ab OR device*:ti,ab OR technolog*:ti,ab)) | |
| #12 | #10 OR #11 | |
| #13 | telemental*:ti,ab,kw OR tele-mental* OR telepsych:ti,ab,kw OR tele-psych*:ti,ab,kw | |
| #14 | #3 AND #6 AND #9 AND #12 | |
| #15 | #3 AND #6 AND #13 | |
| #16 | #14 OR #15 in Cochrane Reviews | |
| #17 | #14 OR #15 in Trials | |

**Web of Science Core Collection –** Science Citations Index, Social Science Citation Index and Conference Proceedings Citation Index - Science

| # | Search Query |
| --- | --- |
| 1 | (TI=(corona* NEAR/1 (virus* OR viral* )) OR AB=(corona* NEAR/1 (virus* OR viral* ))) OR (TI=(CoV NOT (Coefficien* OR co-efficien* OR covalent* OR Covington* OR covariant* OR covarianc* OR "cut-off value*" OR "cutoff value*" OR "cut-off volume*" OR "cutoff volume*" OR "combined optimi$ation value*" OR "central vessel trunk*" OR CoVR OR CoVS )) OR AB=(CoV NOT (Coefficien* OR co-efficien* OR covalent* OR Covington* OR covariant* OR covarianc* OR "cut-off value*" OR "cutoff value*" OR "cut-off volume*" OR "cutoff volume*" OR "combined optimi$ation value*" OR "central vessel trunk*" OR CoVR OR CoVS ))) OR (TI=(coronavirus* OR 2019nCoV* OR 19nCoV* OR "2019 novel*" OR Ncov* OR n-cov OR SARSCoV-2* OR SARSCoV-2* OR SARSCoV2* OR SARS-CoV2* OR "severe acute respiratory syndrome*" OR COVID*2 ) OR AB=(coronavirus* OR 2019nCoV* OR 19nCoV* OR "2019 novel*" OR Ncov* OR n-cov OR SARSCoV-2* OR SARSCoV-2* OR SARSCoV2* OR SARS-CoV2* OR "severe acute respiratory syndrome*" OR COVID*2 )) Editions: WOS.SCI,WOS.SSCI,WOS.ISTP |
| 2 | TS=(depress* OR anxiety OR anxious OR phobia$ OR phobi$ OR "obsessive compulsive disorder$" OR panic OR mood$ OR ((psychological* OR mental* OR emotional* ) NEAR/3 (stress* OR distress* OR disorder$ ))) OR TS=(mental* NEAR/2 (ill* OR health OR disease )) Editions: WOS.SCI,WOS.SSCI,WOS.ISTP |
| 3 | (TS=(psychotherap* OR ((cognitive OR behavio* OR psycho ) NEAR/0 therap* ) OR ((psycho* OR social* OR peer ) NEAR/0 support ) OR counsel$ing OR mindfulness OR meditation OR wellness )) AND (TI=(online OR web OR internet OR digital* ) OR AB=((online OR web OR internet OR digital* ) NEAR/3 (based OR application* OR intervention* OR program* OR therap* )) OR TI=(phone* OR telephone* OR smartphone* OR cellphone* OR smartwatch* ) OR AB=((phone* OR telephone* OR smartphone* OR cellphone* OR smartwatch* ) NEAR/3 (based OR application* OR intervention* OR program* OR therap* )) OR TI=("mobile health" OR mhealth OR m-health OR ehealth OR e-health OR emental OR e-mental ) OR AB=(("mobile health" OR mhealth OR m-health OR ehealth OR e-health OR emental OR e-mental ) NEAR/3 (based OR application* OR intervention* OR program* OR therap* )) OR (TI=(mobile* NEAR/3 (based OR application* OR intervention* OR device* OR technolog* )) OR AB=(mobile* NEAR/3 (based OR application* OR intervention* OR device* OR technolog* )))) OR TS=(telemental* OR telemental* OR telepsych* OR tele-psych*) Editions: WOS.SCI,WOS.SSCI,WOS.ISTP |
| 4 | TS=(random* or blind* or allocat* or assign* or trial* or placebo* or crossover* or cross-over* or multicenter OR "multi center" OR multicentre OR "multi centre" OR intervention$ OR effect$ OR impact$ OR controlled OR "control group$" OR (before NEAR/5 after ) OR (pre NEAR/5 post ) OR ((pretest OR "pre test" ) AND (posttest OR "post test" )) OR quasiexperiment* OR "quasi experiment*" OR "pseudo experiment*" OR pseudoexperiment* OR evaluat* OR "time series" OR "time point$" OR "repeated measur*" ) NOT TS=(animals or animal or mice or mus or mouse or murine or woodmouse or rats or rat or murinae or muridae or cottonrat or cottonrats or hamster or hamsters or cricetinae or rodentia or rodent or rodents or pigs or pig or swine or swines or piglets or piglet or boar or boars or sus scrofa or ferrets or ferret or polecat or polecats or mustela putorius or guinea pigs or guinea pig or cavia or callithrix or marmoset or marmosets or cebuella or hapale or octodon or chinchilla or chinchillas or gerbillinae or gerbil or gerbils or jird or jirds or merione or meriones or rabbits or rabbit or hares or hare or diptera or flies or fly or dipteral or drosphila or drosophilidae or cats or cat or carus or felis or nematoda or nematode or nematoda or nematode or nematodes or sipunculida or dogs or dog or canine or canines or canis or sheep or sheeps or mouflon or mouflons or ovis or goats or goat or capra or capras or rupicapra or chamois or haplorhini or monkey or monkeys or anthropoidea or anthropoids or saguinus or tamarin or tamarins or leontopithecus or hominidae or ape or apes or pan or paniscus or pan paniscus or bonobo or bonobos or troglodytes or pan troglodytes or gibbon or gibbons or siamang or siamangs or nomascus or symphalangus or chimpanzee or chimpanzees or prosimians or bush baby or prosimian or bush babies or galagos or galago or pongidae or gorilla or gorillas or pongo or pygmaeus or pongo pygmaeus or orangutans or pygmaeus or lemur or lemurs or lemuridae or horse or horses or pongo or equus or cow or calf or bull or chicken or chickens or gallus or quail or bird or birds or quails or poultry or poultries or fowl or fowls or reptile or reptilia or reptiles or snakes or snake or lizard or lizards or alligator or alligators or crocodile or crocodiles or turtle or turtles or amphibian or amphibians or amphibia or frog or frogs or bombina or salientia or toad or toads or epidalea calamita or salamander or salamanders or eel or eels or fish or fishes or pisces or catfish or catfishes or siluriformes or arius or heteropneustes or sheatfish or perch or perches or percidae or perca or trout or trouts or char or chars or salvelinus or fathead minnow or minnow or cyprinidae or carps or carp or zebrafish or zebrafishes or goldfish or goldfishes or guppy or guppies or chub or chubs or tinca or barbels or barbus or pimephales or promelas or poecilia reticulata or mullet or mullets or seahorse or seahorses or mugil curema or atlantic cod or shark or sharks or catshark or anguilla or salmonid or salmonids or whitefish or whitefishes or salmon or salmons or sole or solea or sea lamprey or lamprey or lampreys or pumpkinseed or sunfish or sunfishes or tilapia or tilapias or turbot or turbots or flatfish or flatfishes or sciuridae or squirrel or squirrels or chipmunk or chipmunks or suslik or susliks or vole or voles or lemming or lemmings or muskrat or muskrats or lemmus or otter or otters or marten or martens or martes or weasel or badger or badgers or ermine or mink or minks or sable or sables or gulo or gulos or wolverine or wolverines or minks or mustela or llama or llamas or alpaca or alpacas or camelid or camelids or guanaco or guanacos or chiroptera or chiropteras or bat or bats or fox or foxes or iguana or iguanas or xenopus laevis or parakeet or parakeets or parrot or parrots or donkey or donkeys or mule or mules or zebra or zebras or shrew or shrews or bison or bisons or buffalo or buffaloes or deer or deers or bear or bears or panda or pandas or wild hog or wild boar or fitchew or fitch or beaver or beavers or jerboa or jerboas or capybara or capybaras) Editions: WOS.SCI,WOS.SSCI,WOS.ISTP |
| 5 | #4 AND #3 AND #2 AND #1 Editions: WOS.SCI,WOS.SSCI,WOS.ISTP |

**Scopus**

( ( ( TITLE-ABS-KEY ( telemed* OR tele-med* OR telehealth* OR tele-health OR telemental* OR tele-mental* ) ) ) OR ( TITLE-ABS-KEY ( app OR apps ) OR TITLE ( online OR web OR internet OR digital* ) OR ABS ( ( online OR web OR internet OR digital* ) W/3 ( based OR application* OR intervention* OR program* OR therap* ) ) OR TITLE ( phone* OR telephone* OR smartphone* OR cellphone* OR smartwatch* ) OR ABS ( ( phone* OR telephone* OR smartphone* OR cellphone* OR smartwatch* ) W/3 ( based OR application* OR intervention* OR program* OR therap* ) ) OR TITLE ( "mobile health" OR mhealth OR m-health OR ehealth OR e-health OR emental OR e-mental ) OR ABS ( ( "mobile health" OR mhealth OR m-health OR ehealth OR e-health OR emental OR e-mental ) W/3 ( based OR application* OR intervention* OR program* OR therap* ) ) OR TITLE-ABS ( mobile* W/3 ( based OR application* OR intervention* OR device* OR technolog* ) ) ) ) AND ( ( TITLE-ABS-KEY ( corona* W/1 ( virus* OR viral* ) ) ) OR ( TITLE-ABS-KEY ( coronavirus* OR 2019ncov* OR 19ncov* OR "2019 novel*" OR ncov* OR n-cov OR sarscov* OR "severe acute respiratory syndrome*" OR covid* ) ) ) AND ( ( TITLE-ABS-KEY ( psychotherap* OR ( ( cognitive OR behavio* OR psycho ) W/1 therap* ) OR ( ( psycho* OR social* OR peer ) W/1 support ) OR counsel*ing OR mindfulness OR meditation OR wellness ) ) AND ( TITLE-ABS-KEY ( depress* OR anxiety OR anxious OR phobia* OR phobi* OR "obsessive compulsive disorder*" OR panic OR mood* OR ( ( psychological* OR mental* OR emotional* ) W/3 ( stress* OR distress* OR disorder* ) ) ) OR TITLE-ABS-KEY ( mental* W/2 ( ill* OR health OR disease ) ) ) ) AND ( ( TITLE-ABS-KEY ( random* OR blind* OR allocat* OR assign* OR trial* OR placebo* OR crossover* OR cross-over* OR multicenter OR "multi center" OR multicentre OR "multi centre" ) OR TITLE-ABS-KEY ( intervention* OR effect* OR impact* OR controlled OR "control group*" OR quasiexperiment* OR "quasi experiment*" OR "pseudo experiment" ) OR TITLE-ABS-KEY ( before W/5 after ) OR TITLE-ABS-KEY ( pre W/5 post ) OR ( TITLE-ABS-KEY ( pretest OR "pre test" ) AND TITLE-ABS-KEY ( posttest OR "post test" ) ) ) )

**ClinicalTrials.gov**

Other terms=depress* OR anxiety OR stress* OR distress* OR psych* OR mental* AND Condition=COVID-19 AND Intervention=online OR digital* OR internet* OR web* OR app OR apps OR telemed* OR tele-med OR telehealth OR tele-health OR "cell phone*" OR "cellular phone*" OR smartphone*

**WHO ICTRP**

Condition=depress* OR anxiety OR stress* OR distress* OR psych* OR mental* AND Intervention=online OR digital* OR internet* OR web* OR app OR apps OR telemed* OR tele-med OR telehealth OR tele-health OR "cell phone*" OR "cellular phone*" OR smartphone* - Restricted to Covid-19

**Additional online resources and citation searching**

In addition to the bibliographic databases and study registries, Google Scholar was searched as an additional online resource. This search identified 158 records, which were imported into Covidence for screening. Citation searching was also conducted and identified 1 additional record. All records identified through these additional methods were screened using the same eligibility criteria as the database search.

The database searches were updated by rerunning the original search strategies with date limits for newly published records. Update searches were conducted in August 2025 and again on 19 March 2026. The results of the update searches are reported in the Search Report.

**Reference:**

PRISMA-S: An Extension to the PRISMA Statement for Reporting Literature Searches in Systematic Reviews

Rethlefsen ML, Kirtley S, Waffenschmidt S, Ayala AP, Moher D, Page MJ, Koffel JB, PRISMA-S Group.

Last updated February 27, 2020.
