# Supplementary material for: Effectiveness of Telehealth Care Interventions for Women’s Mental Health During and After the COVID-19 Pandemic: Systematic Review and Meta-Analysis
Source: J Med Internet Res. 2026 Sep 21;28:e85494. doi: 10.2196/85494 (PMC13592667; doi:10.2196/85494)
Supplement: Supplementary file 2 [file jmir-v28-e85494-s002.docx]

***Table S1.*** Characteristics of the studies included in the systematic review

| **Study ID** | **Sample Population** | **Design** | **Telemental type/duration** | **Mode** | **Comparator** | **Key Outcomes** | **Key Findings** |
| --- | --- | --- | --- | --- | --- | --- | --- |
| Aligoltabar et al (2025) [100] | N=130 Pregnant women diagnosed with an anxiety disorder | RCT | Guided Emotion-focused CBT, Twice per week for 120 minutes | Internet-based, Synchronous | Standard care | Depression, Anxiety, Stress | The interventions led to significant improvements in anxiety symptoms, pregnancy-specific stress, uncertainty intolerance, and emotional regulation |
| Boucher et al. (2023) [109] | N=288 Healthcare workers | RCT | Unguided Exercise, 4 times per week for 12 weeks. | Web-based, asynchronous | Waitlist | Depression, Burnout, Absenteeism | Although exercise was able to reduce depressive symptoms among HCWs, adherence was low toward the end of the trial |
| Bryant et al (2022) [51] | N=240 pregnant and post-partum women | RCT | Guided Psychological, Mindfulness and behavioural strategies  Weekly for 8 weeks | Video conferencing,  Synchronous | Standard care | Depression, Anxiety, Sleep Quality, Traumatic Stress, Contamination Fears | Provides initial evidence that results in moderate reductions in common psychological problems |
| Chang et al (2022) [75] | N=67 Women with breast cancer | RCT | Guided Mindfulness, 6 times per week for 6 weeks | Video conferencing,  Synchronous | Waitlist | Depression, Anxiety, Stress, Self-efficacy | iMBSR as a program that can improve mental health, body image, and self-efficacy |
| De Kock et al. (2022) [76] | N=73 Health or Social Care worker during COVID-19 | RCT | Unguided Psychological Support, Daily for 4 weeks | Mobile app, Synchronous | Waitlist | Anxiety, Depression, Mental Toughness, Mental Well-being | Encouraging results for the use of brief digital psychological interventions in improving PWB among health and social care workers |
| Dominguez-Rodriguez et al (2024) [110] | N=608 Mexican population | RCT | Unguided Positive psychology, CBT, and BAT ,15 modules, self-paced | Web based platform, Asynchronous | Standard Care | Depression, Anxiety, Fears, Sleep, Coping, Suicide ideation | Seemed to improve the perception of the intervention's usefulness |
| Dominguez-Rodriguez et al (2023) [77] | N=94 Adults during Covid-19 | RCT | Unguided Psychological support, Every 3 days for 36 days | Web based platform, Asynchronous | Waitlist | Grief, Depression, Hopelessness, Anxiety, Suicide Risk | Effective in reducing symptoms of anxiety, depression, hopelessness, suicide risk, PTSD, and complicated grief disorder. |
| Dumarkaite et al (2023) [111] | N=168 Nurses | RCT | Guided CBT, Daily for 6 weeks | Internet-based, Synchronous | Waitlist | Depression, Anxiety, Stress | could increase stress recovery skills, psychological well-being, and reduce stress, anxiety, and depression symptoms |
| Ehlis et al (2024) [104] | N=138  Homestayers during lockdown | RCT | Exercises, Daily for 14 days | Text-based SNS Platform, Asynchronous | No care | Anxiety, Stress | findings encourage the use of low-threshold interventions to support mental health during pandemic crises |
| Fiol-DeRoque et al (2021) [78] | N=436  Health care workers during COVID-19 | RCT | Unguided Mindfulness, Psychological support, Daily for 2 weeks | Mobile app, Synchronous | Standard Care | Depression, Anxiety, Stress, Burnout, Insomnia, Self-efficacy | reduced mental health problems at 2 weeks only among HCW’s receiving psychotherapy or psychotropic medications. |
| Gomà et al (2023) [79] | N=72 Pregnant & postpartum women | RCT | Guided Interdisciplinary therapeutic groups (Bio-Psycho-social support), Weekly for 8 weeks | Videoconferencing, Synchronous | Waitlist | Anxiety, Depression | Helped in the reduction of the mothers’ depressive-anxiety symptomatology. |
| Guerrero-Pertiñez et al (2026) [112] | N=347 MindxYou participants | RCT | Unguided exercises, mindfulness, support, 2 weeks per module for 8 weeks | Web-based platform, Asynchronous | Standard Care | Stress, health, anxiety, psychological disorders | Results demonstrate resilience as the key psychological mediator. |
| Guney et al (2022) [80] | N=84  Pregnant women with a COVID infection within the last 3 months | RCT | Guided Mindfulness, 2 times per week for 4 weeks | Live webbased sessions, Synchronous | Standard Care | Depression, Anxiety, Childbirth fear | program may be utilized to reduce the distress, anxiety and childbirth fear levels of pregnant women diagnosed with COVID-19. |
| Jung and Ko (2024) [103] | N=80  Isolated Covid-19 patients | RCT | Guided Tele-rehabilitaion, 50 minutes daily for 7 days | Phone-based, synchronous | Standard Care | Depression, Anxiety, Insomnia | TR was effective in reducing anxiety and improving quality of life during hospitalization, but had no remained effects after discharge |
| Karing (2022) [82] | N=43  University students | RCT | Guided Mindfulness, weekly for 7 weeks | Videoconferencing group, Synchronous | Standard care | Mindfulness, Depression, Anxiety, Life Satisfaction | Interventions were effective in increasing mindfulness and life satisfaction |
| MacKinnon et al (2022) [113] | N=65 Mothers of pre-school children | RCT | Guided Therapy and parenting education, weekly for 10 weeks | Mobile app, Synchronous | Standard Care | Depression, parenting stress | feasible and acceptable Intervention for improving mental health. |
| Naja et al (2023) [83] | N=33 Pregnant women | RCT | Guided Psychosocial Intervention with videos, 2 sessions of 45 minutes | Videobased app, Synchronous | Standard care | Depression, Covid-19 Phobia | Low-intensity psychosocial telemental maternal sessions effectively reduce antenatal anxiety |
| Pratt et al (2023) [84] | N=102 Nurses at ICUs and Emergency departments | RCT | Unguided Mindfulness Intervention, 1 time | Mobile app, Asynchronous | Waitlist | Depression, Anxiety, Stress, Personal Accomplishment | Participants had a reduction in depression symptoms, but not in burnout. |
| Puertas-Gonzalez et al (2022) [85] | N=138 Pregnant women | RCT | Guided CBG Psychological Support, weekly for 8 weeks | Web based CBT, Synchronous | Standard Care | Depression, Anxiety, Stress, Resilience, Obsession-Compulsion | Participants presented lower rates of pregnancy-specific stress and perceived stress, and greater resilience, lower anxiety, depression and obsessions-compulsions symptoms |
| Rahgozar et al (2024) [86] | N=70  Students in European countries with moderate levels of anxiety | RCT | Guided Logotherapy group, weekly 1 hour for 6 weeks | Study website, Synchronous | Standard Care | Anxiety, Depression | effectively reducing depression and anxiety in such complex scenarios where psychological resources and cultural competencies are limited. |
| Ruzickova et al (2023) [114] | N=68  Mild to low mood individuals | RCT | Guided Behavioral Activation,  1 time | Videoconferencing, synchronous | Waitlist | Depression, Anxiety, Negative affective bias | BA group showed a significant decrease in depression, anxiety and anhedonia after the intervention |
| Sockalingam et al (2022) [87] | N=76 Patients after bariatric surgery | RCT | Guided behavioural therapy | Telephone based, Synchronous | Standard Care | Anxiety, Depression | Tele-CBT is a feasible and effective treatment for improving psychological distress and disordered eating |
| Shahamabadi et al (2022) [88] | N=40  Women with history of miscarriage anxiety and stress. | RCT | Guided Metacognitive education | Videoconferencing, synchronous, weekly for 8 weeks | Standard Care | Anxiety | Online educational counseling was more effective |
| Smith et al (2021) [89] | N=101 Pregnant women | RCT | Unguided Mindfulness, weekly for 30 days | Mobile app “Calm” Asynchronous | Standard Care | Stress, Depression, Anxiety, Sleep Disturbance | Intervention had significant reductions in perceived stress, depression, anxiety, and sleep disturbance |
| Ugarte et al (2023) [90] | N=290  Adults with anxiety | RCT | Guided Social support, 3 times per week for 6 weeks | Facebook, Asynchronous | Standard Care | Anxiety | HOPE intervention effectively promoted mental health help-seeking behavior and online engagement. |
| Van Lieshout et al (2021) [91] | N=404  Women with PPD | RCT | Guided CBT, 4 sessions 1 day | Video conferencing, Synchronous | Waitlist | Anxiety, Depression | effective, brief option for mothers, reducing PPD and anxiety. |
| Vlassopoulos et al (2021) [115] | N=2402 Women in Rural households | RCT | Guided Psychosocial support and telecounseling, Biweekly for 3 months | Telephone / phone calls, Synchronous | Standard Care | Stress, Depression | intervention leads to large improvements in measures of stress and depression severity |
| Wahlund et al (2020) [116] | N=670 Swedish adults | RCT | Unguided Online CBI, self guided time for 3 weeks | Study website, Asynchronous | Waitlist | Anxiety, Depression, Sleep quality | Can significantly reduce dysfunctional worry and associated behavioural symptoms related to the COVID-19 pandemic. |
| Wang et al (2023) [92] | N=70 Nurses | RCT | Guided mindfulness, twice per week for 3 weeks | Video conferencing, Synchronous | Waitlist | Anxiety, Depression, Stress | Proves to alleviate anxiety and related symptoms for up to 6 months. |
| Yook et al (2024) [93] | N=38  Female students | RCT | Guided mindfulness, Twice per week for 4 weeks | Video conferencing, Synchronous | Standard Care | Stress, Anxiety, Social connectedness | Effectively reduced stress and anxiety and improved social connectedness |
| Zhang et al (2023) [94] | N=236 Primiparous women | RCT | Guided Support groups, Twice per week for 3 months | Videoconferencing, Synchronous | Standard Care | Self-efficacy Depression | Significantly increased the levels of MSE, social support, and alleviate PPD symptoms |
| Zhou et al. (2022) [95] | N=118 Nurses on site During Covid-19 | RCT | Guided Sleep improvement tools, 6 weeks | Web-based, Synchronous | Standard Care | Sleep quality, Anxiety, depression | eCBT-I improved the sleep quality during COVID-19 prevention and control period and relieved anxiety and depression |
| Zhang et al (2025) [105] | N=35  Mother-infant dyads | RCT | Guided live educational courses, consultation, 8 weeks | Web-based apps, Synchronous | Standard Care | PPD, PTSD | Effective in improving mothers’ depressive symptoms and their preterm infants’ body growth. |
| Wi et al (2025) [102] | N=45  pregnant women | RCT | Unguided Mindfulness, 6 weeks | Web-based, Asynchronous | Standard Care | Sleep quality, depression | Improves subjective sleep quality and psychological distress, including fatigue, depression, and anxiety during pregnancy |
| Duggal et al (2025) [101] | N=40  perinatal women | RCT | Guided Counseling intervention, 14 calls pre-post pregnancy | Phone-based, Synchronous | Standard Care | Depression, adherence, illness perception, stigma | integration of mobile phone counselling into maternal HIV care shows promise. |
| Arias et al (2022) [52] | N=1579 Postpartum patients | Pre-Post Retrospective Cohort Study | Guided CBT for postpartum care, as needed for 14 weeks | Webbased video/audio,  Synchronous | None | Postpartum measurements | Tele-CBT is a feasible and effective treatment for improving psychological distress |
| Bantjes et al (2021) [67] | N=250 University students | Pre-Post pragmatic trial | Guided Group CBT, Weekly for 10 weeks | Videoconferencing tool, Synchronous | None | Anxiety, Depression | Proof of concept for the use of web-based group CBT to promote mental health |
| Bäuerle et al (2021) [68] | N=220 Adults | Pre-Post Prospective cohort | Unguided Mindfulness, Psychoeducational, Interactive skills. 4 sessions of 30 minutes | Web based education and interactive skills training, Asynchronous | None | Depression, Anxiety, Mindfulness, Self-efficacy | The “CoPE It” intervention seems to be effective |
| Boorboor et al (2022) [50] | N=28 Women suffering from depression | Quasi-experimental Study | Guided Behavioural Activation Therapy, Weekly for 6 weeks | Videoconferencing, Synchronous | Standard Care | Depression, Alexithymia | BA treatment had a positive impact on depression and alexithymia |
| Brooks et al (2024) [69] | N=12 Mothers with young children | Pre-Post Pilot study | Guided Psychoeducation/ Mindfulness, Weekly for 8 weeks | Videoconferencing, Synchronous | None | Anxiety, Depression, Parenting sense of competence | A virtual wellness group was both acceptable and feasible for the six AA mothers |
| Cavarretta et al (2025) [117] | N=95 Psychotherapists | Pre-Post exploratory study | Guided online therapy, 5 months | Videoconferencing, Synchronous | None | Burnout | Online therapy ensures greater workplace flexibility, serving as a protective factor in reducing psychotherapists’ burnout |
| Doornbos et al (2025) [118] | N=47 Women with Depression & Anxiety | Pre-Post test Study | Guided Women Supporting Women Zoom, Weekly for 6 weeks | Videoconferencing, Synchronous | None | Depression, Anxiety | The ability to offer evidence-based WSW may be a viable strategy for enhancing access to care for vulnerable women. |
| Farris et al (2021) [119] | N=233 US migraine patients & HC Providers | Pre-Post Prospective trial | Guided Online mindfulness meditation, single session of 15 minutes | Web-based video Asynchronous | None | Stress, Anxiety, Covid-19 concerns | Virtual mindfulness is an increasingly accessible available world-wide that may reduce psychological distress |
| Gemmill et al (2022) [70] | N=38 Pregnant women | Pre-Post  Feasibility Study | Guided Psychological Support, Weekly for 6 weeks | Video, telephone, Synchronous | None | Depression, Anxiety, Stress | Mum2BMoodBooster is an effective treatment for depressed pregnant women |
| Gonzalez-Garcia et al (2021) [120] | N=66 University students | Pre-Post feasibility study | Unguided Mindfulness/ Compassion-based Intervention, Daily for 16 days | Video’s on Web based training platform, Asynchronous | None | Stress, Anxiety, Self-compassion | brief online mindfulness and compassion intervention may be a feasible way of promoting mental health |
| Hahn et al (2024) [98] | N=161 Women under obstetric care | Prospective cohort | Unguided mindfulness, 6 times during pregnancy | “7mind”‑app, Asynchronous | Standard Care | Depression, Fatigue,  Engagement,  Anxiety | Seems to lead to a clear benefit in the prevention of general anxiety and COVID-19-related anxieties, but needs further research to represent a valid intervention for the prevention of PPD. |
| Haro-Ramos et al (2023) [54] | N=524 Adults with anxiety symptoms | Pre-Post Prospective cohort | Guided Psychological support, Daily for 60 days | One way text messages by StayWell, Asynchronous | None | Stress, Anxiety | StayWell helped reduced depression and anxiety symptoms in our sample of users |
| He et al (2022) [81] | N=62 Nurses | Pre-Post Prospective cohort | Guided  Psychological crisis intervention, around isolation ward time | Messaging app, Synchronous | None | Anxiety, depression levels, and physical symptoms | The intervention can be effective in reducing negative emotions, somatic symptoms and improving the mental health |
| Iravani et al (2023) [53] | N=100 Pregnant women | Quasi-experimental study | Guided Education, regular interactions during 8 weeks | Telephone, text messaging, messaging app, Synchronous | Standard Care | Anxiety | Using tele-med can reduce unnecessary referrals and risk of developing the disease and reduce anxiety. |
| Langhammer et al (2024) [99] | N=1261 Women with mental health concers | Pre-Post Quasi-experimental | Guided CBT, pshycoeducation, bahaviour modification Weekly 100 minutes for 6 weeks | Aury chatbot and digitized group intervention Synchronous | None | Depression, Anxiety | Effectively reduced pandemic-induced mental health issues, indicating its potential in crisis periods with limited health resources |
| Lestari et al (2021) [59] | N=92  Parents of children with autism | Pre-Post Quasi-Experimental Study | Guided Online Supportive Counselling, Twice per week for 4 weeks | Messaging app, Synchronous | None | Anxiety | There was an effect of online counseling on anxiety in parents of children with ASD during COVID19 pandemic |
| Liu et al (2021) [71] | N=125 Parents of children with autism | Quasi-Experimental Study | Guided Parenting Training, 6 sessions per week for 12-20 weeks | Messaging app, Synchronous | Standard Care | Anxiety, Depression, Parenting Stress, Hope | training is acceptable and appears to be an effective approach for reducing anxiety, depression, parenting stress, and hope |
| Merza et al (2023) [121] | N=64 Mothers with Postpartum Depression | Pre-Post  Pilot study | Guided Online Cognitive behavioral therapy-based workshop, 1 time | Videoconferencing, Synchronous | None | Postnatal depression, anxiety, Rejection and pathological anger | The 1-day CBT-based workshops for PPD were feasible and acceptable |
| Ornelas et al (2022) [72] | N=174 Latino immigrant women | Intervention comparison group trial | Guided Online Mindfulness, weekly for 8 weeks | App based, Synchronous | Standard Care | Depression, Anxiety | ALMA intervention is acceptable and feasible in this population. |
| Valinskas et al (2023) [73] | N=762 None clinical users | Pre-Post Cohort study | Unguided CBT, 30 minutes daily for 6 months | Mobile app, Asynchronous | None | Anxiety, Depression, Stress | Using the Sensa mobile app was related to decreased depression, anxiety, and stress symptoms. |
| Van Haeken et al (2024) [74] | N=83 Pregnant women | Quasi-experimental Pilot study | Guided Online Resilience-enhancing intervention, biweekly for 28 weeks | Mobile app & Phone, Synchronous | Standard Care | Resilience, Social Support, Self-esteem, Depression, Anxiety | potential value of a supported online intervention comprising different deployable components |
| Young et al (2025) [122] | N=68 People with emotional distress | Quasi-experimental Pilot study | Brief-Guided I-Cognitive Behavioural Therapy, weekly for 5 weeks | Web based platform & phonecalls, Synchronous | Standard Care | Stress, Anxiety, Depression | Results suggest that brief-guided iCBT is promising for people with emotional problems |

*Standard care participants received the usual, routine or other care/services available in their setting, without receiving the additional telehealth intervention evaluated in the study. *Waitlist participants did not receive the intervention during the study comparison period but were offered access to the intervention after the waiting period or after study completion. *None indicates no control group in the study.

**References**

- Aligoltabar S, Nasiri-Amiri F, Khafri S, et al. Feasibility and acceptability of internet-based emotion-focused cognitive behavioral therapy in women with suspected fetal anomalies: a quasi-experimental study. Jundishapur J Chronic Dis Care. 2025;14(1):e147163. doi:10.5812/jjcdc-147163.
- Boucher VG, Haight BL, Hives BA, et al. Effects of 12 weeks of at-home, application-based exercise on health care workers’ depressive symptoms, burnout, and absenteeism: a randomized clinical trial. JAMA Psychiatry. Nov 1, 2023;80(11):1101-1109. doi:10.1001/jamapsychiatry.2023.2706. Medline:37556150.
- Bryant RA, Dawson KS, Keyan D, et al. Effectiveness of a videoconferencing-delivered psychological intervention for mental health problems during COVID-19: a proof-of-concept randomized clinical trial. Psychother Psychosom. 2022;91(1):63-72. doi:10.1159/000520283. Medline:34875669.
- Chang YC, Chiu CF, Wang CK, Wu CT, Liu LC, Wu YC. Short-term effect of internet-delivered mindfulness-based stress reduction on mental health, self-efficacy, and body image among women with breast cancer during the COVID-19 pandemic. Front Psychol. 2022;13:949446. doi:10.3389/fpsyg.2022.949446. Medline:36389600.
- De Kock JH, Latham HA, Cowden RG, et al. Brief digital interventions to support the psychological well-being of NHS staff during the COVID-19 pandemic: 3-arm pilot randomized controlled trial. JMIR Ment Health. Apr 4, 2022;9(4):e34002. doi:10.2196/34002. Medline:35044927.
- Dominguez-Rodriguez A, Sanz-Gomez S, González Ramírez LP, et al. Evaluation and future challenges in a self-guided web-based intervention with and without chat support for depression and anxiety symptoms during the COVID-19 pandemic: randomized controlled trial. JMIR Form Res. Sep 30, 2024;8:e53767. doi:10.2196/53767. Medline:39348893.
- Dominguez-Rodriguez A, Sanz-Gomez S, González Ramírez LP, et al. The efficacy and usability of an unguided web-based grief intervention for adults who lost a loved one during the COVID-19 pandemic: randomized controlled trial. J Med Internet Res. Apr 6, 2023;25:e43839. doi:10.2196/43839. Medline:36877800.
- Dumarkaite A, Truskauskaite I, Andersson G, et al. The efficacy of the internet-based stress recovery intervention FOREST for nurses amid the COVID-19 pandemic: a randomized controlled trial. Int J Nurs Stud. Feb 2023;138:104408. doi:10.1016/j.ijnurstu.2022.104408. Medline:36527859.
- Ehlis AC, Aas B, Kieckhäfer C, et al. Move, connect and go outside! A randomized controlled trial of two online interventions and analysis of helpful coping strategies in COVID-19 “homestayers”. Appl Psychol Health Well Being. Feb 2025;17(1):e12603. doi:10.1111/aphw.12603. Medline:39414371.
- Fiol-DeRoque MA, Serrano-Ripoll MJ, Jiménez R, et al. A mobile phone-based intervention to reduce mental health problems in health care workers during the COVID-19 pandemic (PsyCovidApp): randomized controlled trial. JMIR Mhealth Uhealth. May 18, 2021;9(5):e27039. doi:10.2196/27039. Medline:33909587.
- Gomà M, Arias-Pujol E, Prims E, et al. Internet-based interdisciplinary therapeutic group (Grupo Interdisciplinar Online, GIO) for perinatal anxiety and depression—a randomized pilot study during COVID-19. Arch Womens Ment Health. Jun 2024;27(3):405-415. doi:10.1007/s00737-023-01412-2. Medline:38150150.
- Guerrero-Pertiñez G, Carbonell-Aranda V, Pérez-Guerrero G, et al. Resilience as a mediator in a web-based intervention (MINDxYOU) to reduce stress among health care professionals: stepped-wedge cluster randomized trial. JMIR Ment Health. Feb 9, 2026;13:e82905. doi:10.2196/82905. Medline:41662682.
- Güney E, Cengizhan SÖ, Karataş Okyay E, Bal Z, Uçar T. Effect of the Mindfulness-Based Stress Reduction program on stress, anxiety, and childbirth fear in pregnant women diagnosed with COVID-19. Complement Ther Clin Pract. May 2022;47:101566. doi:10.1016/j.ctcp.2022.101566. Medline:35219222.
- Jung JH, Ko JY. Depression, anxiety and insomnia among isolated COVID-19 patients: tele occupational therapy intervention vs. conventional one: a comparative study. BMC Psychol. Nov 12, 2024;12(1):649. doi:10.1186/s40359-024-02159-w. Medline:39533442.
- Karing C. The efficacy of online mindfulness-based interventions in a university student sample: videoconference- or podcast-delivered intervention. Appl Psychol Health Well-Being. May 2023;15(2):740-756. doi:10.1111/aphw.12408.
- MacKinnon AL, Simpson KM, Salisbury MR, et al. Building Emotional Awareness and Mental Health (BEAM): a pilot randomized controlled trial of an app-based program for mothers of toddlers. Front Psychiatry. 2022;13:880972. doi:10.3389/fpsyt.2022.880972. Medline:35815022.
- Naja S, Elyamani R, Chehab M, et al. The impact of telemental health interventions on maternal mental health outcomes: a pilot randomized controlled trial during the COVID-19 pandemic. Health Psychol Behav Med. 2023;11(1):1-21. doi:10.1080/21642850.2022.2155167. Medline:36606005.
- Pratt EH, Hall L, Jennings C, et al. Mobile mindfulness for psychological distress and burnout among frontline COVID-19 nurses: a pilot randomized trial. Ann Am Thorac Soc. Oct 2023;20(10):1475-1482. doi:10.1513/AnnalsATS.202301-025OC. Medline:37289650.
- Puertas-Gonzalez JA, Mariño-Narvaez C, Romero-Gonzalez B, Sanchez-Perez GM, Peralta-Ramirez MI. Online cognitive behavioural therapy as a psychological vaccine against stress during the COVID-19 pandemic in pregnant women: a randomised controlled trial. J Psychiatr Res. Aug 2022;152:397-405. doi:10.1016/j.jpsychires.2022.07.016. Medline:35830754.
- Rahgozar S, Giménez-Llort L. Design and effectiveness of an online group logotherapy intervention on the mental health of Iranian international students in European countries during the COVID-19 pandemic. Front Psychiatry. 2024;15:1323774. doi:10.3389/fpsyt.2024.1323774. Medline:38449504.
- Ruzickova T, Carson J, Argabright S, et al. Online behavioural activation during the COVID-19 pandemic decreases depression and negative affective bias. Psychol Med. Feb 2023;53(3):795-804. doi:10.1017/S0033291721002142. Medline:34399873.
- Sockalingam S, Leung SE, Ma C, et al. The impact of telephone-based cognitive behavioral therapy on mental health distress and disordered eating among bariatric surgery patients during COVID-19: preliminary results from a multisite randomized controlled trial. Obes Surg. Jun 2022;32(6):1884-1894. doi:10.1007/s11695-022-05981-6. Medline:35218006.
- Shahamabadi MG, Farajkhoda T, Mahmoodabadi HZ. Online versus face-to-face metacognitive educational counseling program on anxiety and meta-worry in women with a history of miscarriage: a randomized clinical trial. J Educ Health Promot. Jan 1, 2022;11:264. doi:10.4103/jehp.jehp_1292_21. Medline:36325205.
- Smith RB, Mahnert ND, Foote J, Saunders KT, Mourad J, Huberty J. Mindfulness effects in obstetric and gynecology patients during the coronavirus disease 2019 (COVID-19) pandemic: a randomized controlled trial. Obstet Gynecol. Jun 1, 2021;137(6):1032-1040. doi:10.1097/AOG.0000000000004316. Medline:33957663.
- Ugarte DA, Cumberland WG, Singh P, Saadat S, Garett R, Young SD. A HOPE online community peer support intervention for help seeking: a randomized controlled trial. Psychiatr Serv. Jun 1, 2023;74(6):648-651. doi:10.1176/appi.ps.20220168.
- Van Lieshout RJ, Layton H, Savoy CD, et al. Effect of online 1-day cognitive behavioral therapy-based workshops plus usual care vs usual care alone for postpartum depression: a randomized clinical trial. JAMA Psychiatry. Nov 1, 2021;78(11):1200-1207. doi:10.1001/jamapsychiatry.2021.2488. Medline:34495285.
- Vlassopoulos M, Siddique A, Rahman T, Pakrashi D, Islam A, Ahmed F. Improving women’s mental health during a pandemic. Am Econ J Appl Econ. Apr 1, 2024;16(2):422-455. doi:10.1257/app.20210655.
- Wahlund T, Mataix-Cols D, Olofsdotter Lauri K, et al. Brief online cognitive behavioural intervention for dysfunctional worry related to the COVID-19 pandemic: a randomised controlled trial. Psychother Psychosom. 2021;90(3):191-199. doi:10.1159/000512843. Medline:33212440.
- Wang M, Zhang H, Zhang X, et al. Effects of a online brief modified mindfulness-based stress reduction therapy for anxiety among Chinese adults: a randomized clinical trial. J Psychiatr Res. May 2023;161:27-33. doi:10.1016/j.jpsychires.2023.03.009. Medline:36893668.
- Yook YS, Lee JH, Park I, Cho HY. Effects of online mindful somatic psychoeducation program on mental health during the COVID-19. Psychiatry Investig. Jan 2024;21(1):63-73. doi:10.30773/pi.2023.0304. Medline:38198829.
- Zhang Y, Zhu J, Li S, Huang L, Fang Q, Zheng X. The effectiveness of an internet-based support program on maternal self-efficacy, postpartum depression and social support for primiparous women during the COVID-19 pandemic: randomized controlled trial. Front Public Health. 2023;11:1035872. doi:10.3389/fpubh.2023.1035872.
- Zhou K, Kong J, Wan Y, et al. Positive impacts of e-aid cognitive behavioural therapy on the sleep quality and mood of nurses on site during the COVID-19 pandemic. Sleep Breath. Dec 2022;26(4):1947-1951. doi:10.1007/s11325-021-02547-1. Medline:34997900.
- Zhang J, Chen B, Yang X, et al. An internet-based developmental home care support program improved maternal psychological symptoms and infant growth: a randomized controlled trial. J Perinat Neonatal Nurs. 2025;39(1):80-90. doi:10.1097/JPN.0000000000000823. Medline:39325979.
- Wi D, Lee RY, Kantrowitz-Gordon I. Efficacy of online mindfulness for the treatment of insomnia in pregnancy: a randomized clinical trial. PLoS One. 2025;20(5):e0322931. doi:10.1371/journal.pone.0322931. Medline:40343904.
- Duggal M, Sarna A, Dahiya N, et al. A pilot study to evaluate the feasibility and acceptability of a mobile phone counselling intervention for perinatal women living with HIV and depression in India. Oxf Open Digit Health. 2025;3:oqaf012. doi:10.1093/oodh/oqaf012. Medline:40486985.
- Arias MP, Wang E, Leitner K, et al. The impact on postpartum care by telehealth: a retrospective cohort study. Am J Obstet Gynecol MFM. May 2022;4(3):100611. doi:10.1016/j.ajogmf.2022.100611. Medline:35331971.
- Bantjes J, Kazdin AE, Cuijpers P, et al. A web-based group cognitive behavioral therapy intervention for symptoms of anxiety and depression among university students: open-label, pragmatic trial. JMIR Ment Health. May 27, 2021;8(5):e27400. doi:10.2196/27400. Medline:34042598.
- Bäuerle A, Jahre L, Teufel M, et al. Evaluation of the e-mental health mindfulness-based and skills-based “CoPE It” intervention to reduce psychological distress in times of COVID-19: results of a bicentre longitudinal study. Front Psychiatry. 2021;12:768132. doi:10.3389/fpsyt.2021.768132. Medline:34803775.
- Boorboor N, Haddadian Nekah M, Hamidi B, Uosefi M, Eskandarian F, Mousavi S. Online behavioral activation therapy on depression and alexithymia in infertile women during the COVID-19 pandemic lockdown. Arch Hyg Sci. Oct 10, 2022;11(3):210-215. doi:10.34172/AHS.11.3.315.4.
- Brooks KM, Charlot-Swilley D, Robertson HA, et al. “A judgment-free zone”: adaptation and pilot study of a virtual wellness group for African American mothers with young children. Int J Environ Res Public Health. Mar 23, 2024;21(4):390. doi:10.3390/ijerph21040390. Medline:38673302.
- Cavarretta MV, Pellerin H, Maurel E, et al. The effectiveness of online therapy in promoting wellbeing and reducing burnout among psychotherapists. Front Psychol. 2025;16:1510383. doi:10.3389/fpsyg.2025.1510383. Medline:40230990.
- Doornbos MM, Zandee GL, Bajema AG, Luchies LB. Women Supporting Women Zoom: outcomes of online supportive/educative groups for diverse women experiencing depression and anxiety. Arch Psychiatr Nurs. Aug 2025;57:151934. doi:10.1016/j.apnu.2025.151934. Medline:40816797.
- Farris SR, Grazzi L, Holley M, et al. Online mindfulness may target psychological distress and mental health during COVID-19. Glob Adv Health Med. 2021;10:21649561211002461. doi:10.1177/21649561211002461. Medline:34497735.
- Gemmill AW, Oliva JL, Ericksen J, Holt C, Holt CJ, Milgrom J. Web-based treatment for depression in pregnancy: a feasibility study of Mum2BMoodBooster. BMC Psychiatry. Jul 16, 2022;22(1):476. doi:10.1186/s12888-022-04111-x. Medline:35842616.
- González-García M, Álvarez JC, Pérez EZ, Fernandez-Carriba S, López JG. Feasibility of a brief online mindfulness and compassion-based intervention to promote mental health among university students during the COVID-19 pandemic. Mindfulness (N Y). 2021;12(7):1685-1695. doi:10.1007/s12671-021-01632-6. Medline:34025814.
- Hahn L, Thomann M, Dreyer EM, et al. Efficacy of the “7mind”-app as intervention to prevent postpartum depression in a low-risk sample group. Arch Gynecol Obstet. Dec 2024;310(6):3183-3193. doi:10.1007/s00404-024-07851-1. Medline:39630223.
- Haro-Ramos AY, Rodriguez HP, Aguilera A. Effectiveness and implementation of a text messaging intervention to reduce depression and anxiety symptoms among Latinx and Non-Latinx white users during the COVID-19 pandemic. Behav Res Ther. Jun 2023;165:104318. doi:10.1016/j.brat.2023.104318. Medline:37146444.
- He C, Chang S, Lu Y, et al. Effects of online psychological crisis intervention for frontline nurses in COVID-19 pandemic. Front Psychiatry. 2022;13:937573. doi:10.3389/fpsyt.2022.937573. Medline:35903639.
- Iravani M, Bahmaei H, Askari S, Ghanbari S, Nasab MB, Masihi S. Effect of tele-medicine on health anxiety and pregnancy-related anxiety in pregnant women during the COVID-19 epidemic in Iran. Iran J Nurs Midwifery Res. 2023;28(4):405-410. doi:10.4103/ijnmr.ijnmr_405_21. Medline:37694204.
- Langhammer T, Hilbert K, Wasenmueller R, et al. Evaluation of a CBT-based program for mental health in the general population during the COVID-19 pandemic: a stepped-care approach using a chatbot and digitized group intervention. Depress Anxiety. 2024;2024:8950388. doi:10.1155/2024/8950388. Medline:40226668.
- Lestari NE, Anindya I. Does online counseling reduce anxiety in parents of children with autism spectrum disorders during coronavirus disease 2019 pandemic? Open Access Maced J Med Sci. Sep 9, 2021;9(G):66-70. doi:10.3889/oamjms.2021.6492.
- Liu G, Wang S, Liao J, et al. The efficacy of WeChat-based parenting training on the psychological well-being of mothers with children with autism during the COVID-19 pandemic: quasi-experimental study. JMIR Ment Health. Feb 10, 2021;8(2):e23917. doi:10.2196/23917. Medline:33481751.
- Merza D, Amani B, Savoy C, et al. Online peer-delivered group cognitive-behavioral therapy for postpartum depression: a randomized controlled trial. Acta Psychiatr Scand. Nov 2024;150(5):422-432. doi:10.1111/acps.13611. Medline:37649448.
- Ornelas IJ, Perez G, Maurer S, et al. Amigas Latinas Motivando el Alma: in-person and online delivery of an intervention to promote mental health among Latina immigrant women. J Integr Complement Med. Oct 2022;28(10):821-829. doi:10.1089/jicm.2022.0491. Medline:35723668.
- Valinskas S, Nakrys M, Aleknavicius K, Jonusas J. Sensa mobile app for managing stress, anxiety, and depression symptoms: pilot cohort study. JMIR Form Res. Apr 13, 2023;7:e40671. doi:10.2196/40671. Medline:37052990.
- Van Haeken S, Braeken MAKA, Groenen A, Bogaerts A. A supported online resilience-enhancing intervention for pregnant women: a non-randomized pilot study. Int J Environ Res Public Health. Feb 10, 2024;21(2):209. doi:10.3390/ijerph21020209. Medline:38397698.
- Young DKW, Carlbring P, Ng SM, et al. Brief-guided internet-based cognitive behavioural therapy for people with emotional distress during the COVID-19 pandemic. Clin Soc Work J. Jun 2026;54(2):261-273. doi:10.1007/s10615-025-00990-1.
