# Supplementary material for: Effectiveness of Telehealth Care Interventions for Women’s Mental Health During and After the COVID-19 Pandemic: Systematic Review and Meta-Analysis
Source: J Med Internet Res. 2026 Sep 21;28:e85494. doi: 10.2196/85494 (PMC13592667; doi:10.2196/85494)
Supplement: Supplementary file 3 [file jmir-v28-e85494-s003.docx]

| **Study number** | **Study and publication year**  **Table S1. Results of the RoB 2.0 (2019) assessment for the randomized controlled studies included in the systematic review** | **randomization process** | **deviations from intended interventions** | **Missing outcome data** | **measurement of the outcome** | **selection of the reported result** | **Overall risk of bias (per outcome)** | **Justification** |
| --- | --- | --- | --- | --- | --- | --- | --- | --- |
| 1 | Aligatobar et al. (2025) [100] | Low | Low | Some concerns | Low | Low | Some Concerns | Some attrition found in the outcomes |
| 2 | Boucher et al. (2023) [109] | Low | Low | Some concerns | Low | Low | Some concerns | Study population >60% were nurses and >80% were females. Not representative of the full population in question |
| 3 | Bryant et al. (2022) [51] | Low | Low | Low | Some concerns | Low | Some Concerns | Self report only accurate for Depression, Anxiety and HADS scores. Conducted in Australia, the rates of COVID-19 infections, hospitalizations, and deaths much lower than in many other countries |
| 4 | Chang et al. (2022) [75] | Low | Some concerns | Low | Low | Low | Some Concerns | High dropout rate, switch from CG to intervention group. Intervention not focused on Depression. |
| 5 | De Kock et al. (2022) [76] | Low | Low | Low | Low | Low | Low | some outcomes may change more gradually and require a longer period to improve |
| 6 | Dominguez-Rodriguez et al. (2024) [110] | Low | Low | Some concerns | Low | Low | Some Concerns | Significant Difference in baselines scores between groups. Inclusion of Participants who had access to technology and were actively looking for support |
| 7 | Dominguez-Rodriguez et al. (2023) [77] | Low | Some concerns | High | Low | Low | High | Only 5% of IG participants completed intervention. |
| 8 | Dumarkaite et.al (2023) [111] | Low | Low | Low | Low | Low | Low | Focussed on medical nurses which limits generalizability. All participants were self-referred |
| 9 | Ehlis et al. (2024) [104] | Low | Low | Some concerns | Low | Low | Some Concerns | Small sample size with mostly young people. Dropouts had higher depression, Stress and well-being scores |
| 10 | Fiol-DeRoque et al. (2021) [78] | Low | Low | Low | Low | Low | Low | Population mental health data was not unform at baseline. |
| 11 | Goma et al. (2023) [79] | Low | Low | Low | Low | Some concerns | Some Concerns | Small sample size. Short follow-up |
| 12 | Guerrero-Pertiñez et al. (2026) [112] | Low | Low | Some concerns | Some concerns | Low | High | Volunteer population and study lacked a parallel control group |
| 13 | Guney et al. (2022) [80] | Low | Low | Some concerns | Some concerns | Low | High | Not measured long term effects |
| 14 | Jung & Ko (2024) [103] | Low | Low | Some concerns | Low | Low | Some Concerns | Small sample size. Significant difference in baseline depression scores |
| 15 | Karing et al. (2022) [82] | Low | Low | Some concerns | Low | Low | Some Concerns | Self-reported measures and no follow-up |
| 16 | MacKinnon et al (2022) [113] | Low | Low | Some concerns | Some concerns | Low | High | High attrition rate (45.5%) and small sample size.  There were no differences between groups in medication or mental health service use at enrollment, however data was not collected on other forms of treatment during the trial, which could potentially obscure the findings |
| 17 | Naja et al. (2023) [83] | Low | Low | Low | Low | Low | Low | Study conducted in Qatar during Covid-19 |
| 18 | Pratt et al. (2023) [84] | Some concerns | Some concerns | Some concerns | Some concerns | Low | High | individual-based wellness interventions may be susceptible to selection bias. Sample population does not reflect the true diversity of the nursing community at large |
| 19 | Puertas-Gonzalez et al. (2022) [85] | Low | Low | Low | Low | Low | Low | variables were measured at two different moments in time without any follow-up. |
| 20 | Rahgozar et al. (2024) [86] | Low | Low | Low | Low | Low | Low | Iranian international students in Europe could influence outcomes |
| 21 | Ruzickova et al. (2023) [114] | Low | Low | Low | Low | Low | Low | No serious bias |
| 22 | Sockalingam et al. (2022) [87] | Low | Some concerns | Low | Low | Low | Some Concerns | should only include participants with clinically significant distress |
| 23 | Shahamabadi et al. (2022) [88] | Low | Low | Low | Low | Low | Low | Homogenous sample. |
| 24 | Smith et al. (2021) [89] | Low | Low | Low | Low | Low | Low | No serious bias |
| 25 | Ugarte et al. (2023) [90] | Low | Low | Low | Low | Low | Low | No serious bias |
| 26 | Van Lieshout et al. (2021) [91] | Low | Low | Low | Low | Low | Low | No serious bias |
| 27 | Vlassopoulos et al. (2021) [115] | Low | Low | Low | Low | Low | Low | Big sample of women in rural households in Bangladesh. |
| 28 | Wahlund et al. (2020) [116] | Low | Low | Low | Some concerns | Low | Some Concerns | self-reported outcomes. Limited generalizability of findings to other contexts |
| 29 | Wang et al. (2023) [92] | Low | Some concerns | Low | Low | Low | Some Concerns | over representation of females and high educational level in participant groups However, a higher prevalence of anxiety and depression is typically reported in women |
| 30 | Yook et al. (2024) [93] | Some concerns | Low | Low | Low | Low | Low | Small sample size. Participants could self-apply |
| 31 | Zhang et al. (2023) [94] | Low | Low | Low | Low | Low | Low | No serious bias |
| 32 | Zhou et al. (2022) [95] | Low | Low | Low | Low | Low | Low | No serious bias |
| 33 | Zhang et al. (2025) [105] | Low | Low | Low | Low | Low | Low | No serious bias |
| 34 | Wi et al. (2025) [102] | Low | Low | Some concerns | Low | Low | Some Concerns | Small sample and absence of demographic diversity |
| 35 | Duggal et al. (2025) [101] | Low | Low | Some concerns | Low | Low | Some Concerns | Small sample size, self reported outcomes |

| Study  **Table S2. Results of the ROBINS-I (2016) assessment for the non-randomized studies included in the systematic review** | Bias due to confounding | Bias in selection of participants | Bias in classification of interventions | Bias due to deviations from intended interventions | Bias due to missing data | Bias in measurement of outcomes | Bias in selection of the reported result | Overall RoB | Justification |
| --- | --- | --- | --- | --- | --- | --- | --- | --- | --- |
| Arias et al. (2022) [52] | Serious | Moderate | Moderate | Serious | Low | Low | Moderate | Serious | Pre-post covid implementation comparison. Care likely more then intervention alone. Adjusts for some confounding but can not exclude the pandemic |
| Bantjes et al. (2021) [67] | Serious | Serious | Low | Moderate | Serious | Moderate | Moderate | Critical | No control group but used ANOVA for confounding Only analysis of patients who completed follow-up. Self reported outcomes |
| Bäuerle et al. (2021) [68] | Serious | Low | Low | Moderate | Serious | Moderate | Low | Serious | No control group but used ANOVA for confounding , overall dropout rate of 74.09%, self reported outcomes |
| Boorboor et al. (2022) [50] | Moderate | Low | Low | Moderate | Moderate | Moderate | Moderate | Moderate | Used a validated tool(ANCOVA) to control confounding |
| Brooks et al. (2024) [69] | Critical | Serious | Low | Moderate | Low | Moderate | Moderate | Critical | Selected based on interest and availability, also rewarded. Outcomes based on participants’ own reports in a very small sample and no control group |
| Cavarretta et al. (2025) [117] | Critical | Moderate | Moderate | Low | Moderate | Moderate | Moderate | Critical | Exposure was not randomly assigned. Self reported outcomes |
| Doornbos et al. (2025) [118] | Critical | Low | Low | Moderate | Serious | Moderate | Moderate | Critical | Analysed only participants with complete follow-up, Self reported outcomes and no control group |
| Farris et al. (2021) [119] | Critical | Low | Low | Moderate | Serious | Serious | Moderate | Critical | Only 144/233 pre-surveys were used for analysis, self reported outcomes after only one session and no control group |
| Gemmill et al. (2022) [70] | Critical | Moderate | Low | Serious | Serious | Moderate | Moderate | Critical | Extra support (coach calls), observed-case analysis, self reported outcomes and no control group |
| Gonzalez-Garcia et al. (2021) [120] | Critical | Low | Low | Moderate | Moderate | Moderate | Moderate | Critical | No control group and no confounding tool. Self reported outcomes |
| Hahn et al. (2024) [98] | Moderate | Low | Low | Serious | Moderate | Moderate | Moderate | Serious | Randomized groups and used ANOVA but baseline EPDS score is already low. Women with more burden used the app less frequent |
| Haro-Ramos et al. (2023) [54] | Critical | Moderate | Low | Moderate | Serious | Moderate | Moderate | Critical | 65.8% completed pre and post surveys, Self reported outcomes and no control group |
| He et al. (2022) [81] | Critical | Moderate | Low | Serious | Serious | Moderate | Moderate | Critical | 120 enrolments only 62 completions, Self reported outcomes and no control group |
| Iravani et al. (2023) [53] | Moderate | Moderate | Low | Serious | Low | Moderate | Moderate | Serious | Intervention group can contact the researcher 24/7 |
| Langhammer et al. (2024) [99] | Serious | Serious | Moderate | Serious | Serious | Moderate | Moderate | Critical | No control group. Selection Second round of intervention after first round based on symptoms |
| Lestari et al. (2021) [59] | Critical | Moderate | Low | Moderate | Low | Serious | Moderate | Critical | No control group and no tool used for confounding. Self reported outcomes |
| Liu et al. (2021) [71] | Moderate | Moderate | Low | Moderate | Moderate | Moderate | Low | Moderate | Confounding could affect both group choice and outcomes, although baseline characteristics were reported as not significantly different. |
| Merza et al. (2023) [121] | Critical | Moderate | Low | Moderate | Moderate | Serious | Moderate | Critical | No control group, self referral and no confounding tool, Self reported outcomes |
| Ornelas et al. (2022) [72] | Serious | Moderate | Low | Low | Moderate | Moderate | Moderate | Serious | Delivery “ after the onset of the pandemic” No tool used for confounding |
| Valinskas et al. (2023) [73] | Serious | Moderate | Low | Moderate | Serious | Moderate | Moderate | Serious | No control group but used ANOVA for confounding. Only participants who completed DASS-21 assessment at least 2 times. Self-reported data |
| Van Haeken et al. (2024) [74] | Low | Moderate | Low | Moderate | Serious | Moderate | Moderate | Serious | Controlled for confounding, Technical problems at follow-up T6 |
| Young et al. (2025) [122] | Low | Moderate | Low | Moderate | Moderate | Moderate | Moderate | Moderate | quasi-experimental research design with a 3-arm study, used ANOVA for confounding. |

**Table S3. Results of the GRADE per outcome for the randomized studies included in the systematic review**

| **Study ID** | **Outcome** | **No. Of participants** | **Risk of Bias (limitations)** | **Inconsistancy** | **Indirectness** | **Imprecision** | **Certainty (GRADE)** | **Comments** |
| --- | --- | --- | --- | --- | --- | --- | --- | --- |
| Aligatobar et al. (2025) [100] | State Anxiety | 130 | Serious | Not applicable | Serious | Not serious | ⊕⊕◯◯ | Strong population no big baseline differences. |
| Aligatobar et al. (2025) [100] | Pregnancy Stress | 130 | Serious | Not applicable | Serious | Not serious | ⊕⊕◯◯ |  |
|  |  |  |  |  |  |  |  |  |
| Boucher et al. (2023) [109] | Depression | 288 | Serious | Not applicable | Serious | Not serious | ⊕⊕◯◯ | Study population should cover all HCW, yet >60% were nures and >80% were females. Not representative of the full population in question |
|  |  |  |  |  |  |  |  |  |
| Bryant et al. (2022) [51] | Depression | 240 | Not Serious | Not applicable | Not serious | Not serious | ⊕⊕⊕⊕ | No serious risk of bias. Anhedonia outcome only had relativly wide 95% CI ranges |
| Bryant et al. (2022) [51] | Anxiety | 240 | Serious | Not applicable | Not serious | Not serious | ⊕⊕⊕◯ |  |
| Bryant et al. (2022) [51] | Traumatic stress | 240 | Serious | Not applicable | Not serious | Not serious | ⊕⊕⊕◯ |  |
|  |  |  |  |  |  |  |  |  |
| Chang et al. (2022) [75] | Depression | 67 | Serious | Not applicable | Not serious | Not serious | ⊕⊕⊕◯ | Small sample size for an RCT with high rate of drop-out and between groups shift in participants (from control group to intrvention group) |
| Chang et al. (2022) [75] | Anxiety | 67 | Not serious | Not applicable | Not serious | Not serious | ⊕⊕⊕⊕ |  |
| Chang et al. (2022) [75] | Stress | 67 | Not serious | Not applicable | Not serious | Serious | ⊕⊕⊕◯ |  |
|  |  |  |  |  |  |  |  |  |
| De Kock et al. (2022) [76] | Depression | 169 | Serious | Not applicable | Not serious | Very Serious | ⊕◯◯◯ | High attrition rate (36%). Wide 95% probably due to small sample size (may have contributed to statistical uncertainty). High risk of bias due to a small sample of HSCWs from a single NHS site |
| De Kock et al. (2022) [76] | Anxiety | 169 | Not serious | Not applicable | Not serious | Very Serious | ⊕◯◯◯ |  |
|  |  |  |  |  |  |  |  |  |
| Dominguez-Rodriguez et al. (2024) [110] | Depression | 36 | Serious | Not applicable | Serious | Not serious | ⊕⊕◯◯ | Small sample size for an RCT, significant difference in depressive symptoms at baseline between the participants of both groups, and bias in inclusion of participants who had access to tchnology and were actively seeking menatal support. |
| Dominguez-Rodriguez et al. (2024) [110] | Anxiety | 36 | Serious | Not applicable | Not serious | Not serious | ⊕⊕⊕◯ |  |
| Dominguez-Rodriguez et al. (2024) [110] | PTSD | 36 | Serious | Not applicable | Not serious | Not serious | ⊕⊕⊕◯ |  |
|  |  |  |  |  |  |  |  |  |
| Dominguez-Rodriguez et al. (2023) [77] | Depression | 114 | Very serious | Not applicable | Not serious | Not serious | ⊕⊕◯◯ | Very high drop-out rate, where only 5% of IG participants completed the intervention. |
| Dominguez-Rodriguez et al. (2023) [77] | Anxiety | 114 | Very serious | Not applicable | Not serious | Not serious | ⊕⊕◯◯ |  |
|  |  |  |  |  |  |  |  |  |
| Dumarkaite et.al (2023) [111] | Perceived Stress | 168 | Serious | Not applicable | Not serious | Not serious | ⊕⊕⊕◯ | No major risk of bias on study design or exceution of the RCT affecting the certainty of evidence |
| Dumarkaite et.al (2023) [111] | Depression | 168 | Not serious | Not applicable | Not serious | Not serious | ⊕⊕⊕⊕ |  |
| Dumarkaite et.al (2023) [111] | Anxiety | 168 | Not serious | Not applicable | Not serious | Not serious | ⊕⊕⊕⊕ |  |
|  |  |  |  |  |  |  |  |  |
| Ehlis et al. (2024) [104] | Depression | 73 | Serious | Not applicable | Serious | Not serious | ⊕⊕◯◯ | Small sample size, mostly young people. Short follow-up period. More dropouts in intervention groups then Control group. Dropouts higher depression, Stress and well-being scores |
| Ehlis et al. (2024) [104] | Anxiety | 73 | Not serious | Not applicable | Serious | Not serious | ⊕⊕⊕◯ |  |
| Ehlis et al. (2024) [104] | Stress | 73 | Serious | Not applicable | Serious | Not serious | ⊕⊕◯◯ |  |
|  |  |  |  |  |  |  |  |  |
| Fiol-DeRoque et al. (2021) [78] | Depression | 482 | Serious | Not applicable | Not serious | Not serious | ⊕⊕⊕◯ | Short follow-up duration for only 2 weeks post-intervention, population mental health status was not uniform at baseline, it was technically unfeasible to monitor use of the intervention, which prevented us to explore a dose-response effect. |
| Fiol-DeRoque et al. (2021) [78] | Anxiety | 482 | Serious | Not applicable | Not serious | Not serious | ⊕⊕⊕◯ |  |
| Fiol-DeRoque et al. (2021) [78] | Stress | 482 | Serious | Not applicable | Not serious | Not serious | ⊕⊕⊕◯ |  |
|  |  |  |  |  |  |  |  |  |
| Goma et al. (2023) [79] | Depression | 72 | Not serious | Not applicable | Serious | Not serious | ⊕⊕⊕◯ | Small sample size, history of mental health problems was not uniform across treatemnt and control group leading to high risk of bias |
| Goma et al. (2023) [79] | State-Anxiety | 72 | Very serious | Not applicable | Serious | Not serious | ⊕◯◯◯ |  |
|  |  |  |  |  |  |  |  |  |
| Guerrero-Pertiñez et al. (2026) [112] | Perceived Stress | 357 | Serious | Not applicable | Not serious | Not serious | ⊕⊕⊕◯ | Study lacked a parallel control group, which restricts the ability to draw firm causal conclusions beyond the stepped-wedge design. sample may present a selection bias, as it consisted of HCPs who voluntarily chose to participate |
| Guerrero-Pertiñez et al. (2026) [112] | Depression | 357 | Very serious | Not applicable | Not serious | Not serious | ⊕⊕◯◯ |  |
| Guerrero-Pertiñez et al. (2026) [112] | Anxiety | 357 | Serious | Not applicable | Not serious | Not serious | ⊕⊕⊕◯ |  |
|  |  |  |  |  |  |  |  |  |
| Guney et al. (2022) [80] | Anxiety | 84 | Very serious | Not applicable | Not serious | Not serious | ⊕⊕◯◯ | small sample size. Longterm effect of intervention in the post-natal duration was not evaluated. |
|  |  |  |  |  |  |  |  |  |
| Jung & Ko (2024) [103] | Depression | 40 | Serious | Not applicable | not serious | not serious | ⊕⊕⊕◯ | Very small sample size. Significant difference in baseline depression scores. Self report outcomes |
| Jung & Ko (2024) [103] | Anxiety | 40 | Serious | Not applicable | not serious | not serious | ⊕⊕⊕◯ |  |
|  |  |  |  |  |  |  |  |  |
| Karing et al. (2022) [82] | Depression | 50 | Serious | Not applicable | Serious | Not serious | ⊕⊕◯◯ | Small sample size and high drop-out rate. risk of response bias and common method bias due to self-report measures only. absence of a follow-up assessment beyond the end of the intervention |
| Karing et al. (2022) [82] | Anxiety | 50 | Serious | Not applicable | Serious | Not serious | ⊕⊕◯◯ |  |
|  |  |  |  |  |  |  |  |  |
| MacKinnon et al (2022) [113] | Maternal Depression | 65 | Very serious | Not applicable | Not serious | Not serious | ⊕⊕◯◯ | small sample size. High attrition rate (45.5%) |
| MacKinnon et al (2022) [113] | Parenting stress | 65 | Very serious | Not applicable | Not serious | Not serious | ⊕⊕◯◯ |  |
| MacKinnon et al (2022) [113] | Anxiety | 65 | Serious | Not applicable | Not serious | Not serious | ⊕⊕⊕◯ |  |
|  |  |  |  |  |  |  |  |  |
| Naja et al. (2023) [83] | Antenatal anxiety | 58 | Very serious | Not applicable | Not serious | Not serious | ⊕⊕◯◯ | small sample size and higher than expected attrition rate |
| Naja et al. (2023) [83] | Depression | 58 | Not serious | Not applicable | Not serious | Not serious | ⊕⊕⊕⊕ |  |
|  |  |  |  |  |  |  |  |  |
| Pratt et al. (2023) [84] | Depression | 102 | Very serious | Not applicable | serious | not serious | ⊕◯◯◯ | individual-based wellness interventions may be susceptible to selection bias. Sample population does not reflect the true diversity of the nursing community at large |
| Pratt et al. (2023) [84] | Anxiety | 102 | Very serious | Not applicable | serious | not serious | ⊕◯◯◯ |  |
| Pratt et al. (2023) [84] | Stress | 102 | Serious | Not applicable | serious | not serious | ⊕⊕◯◯ |  |
|  |  |  |  |  |  |  |  |  |
| Puertas-Gonzalez et al. (2022) [85] | Stress | 207 | Not serious | Not applicable | not serious | not serious | ⊕⊕⊕⊕ | variables were measured at two different moments in time without any follow-up. |
| Puertas-Gonzalez et al. (2022) [85] | Depression | 207 | Serious | Not applicable | not serious | not serious | ⊕⊕⊕◯ |  |
| Puertas-Gonzalez et al. (2022) [85] | Anxiety | 207 | Serious | Not applicable | not serious | not serious | ⊕⊕⊕◯ |  |
|  |  |  |  |  |  |  |  |  |
| Rahgozar et al. (2024) [86] | Anxiety | 70 | Not serious | Not applicable | serious | not serious | ⊕⊕⊕◯ | small sample size. variations in cultural contexts among international students could introduce potential biases and influence the study’s outcomes |
| Rahgozar et al. (2024) [86] | Depression | 70 | Not serious | Not applicable | serious | not serious | ⊕⊕⊕◯ |  |
|  |  |  |  |  |  |  |  |  |
| Ruzickova et al. (2023) [114] | Depression | 68 | Not serious | Not applicable | not serious | not serious | ⊕⊕⊕⊕ | small sample size. lack of control over the conditions under which participants completed online data collection |
| Ruzickova et al. (2023) [114] | Anxiety | 68 | Not serious | Not applicable | not serious | not serious | ⊕⊕⊕⊕ |  |
|  |  |  |  |  |  |  |  |  |
| Sockalingam et al. (2022) [87] | Anxiety | 81 | Serious | Not applicable | not serious | not serious | ⊕⊕⊕◯ | short FU period and small sample size. Sel-report measures only lead to common method bias, should only include participants with clinically significant distress |
| Sockalingam et al. (2022) [87] | Depression | 81 | Serious | Not applicable | not serious | not serious | ⊕⊕⊕◯ |  |
|  |  |  |  |  |  |  |  |  |
| Shahamabadi et al. (2022) [88] | Anxiety | 80 | Not serious | Not applicable | not serious | not serious | ⊕⊕⊕⊕ | small sample size but homogenous population of women with a history of miscarriage |
|  |  |  |  |  |  |  |  |  |
| Smith et al. (2021) [89] | Perceived Stress | 101 | Not serious | Not applicable | serious | serious | ⊕⊕◯◯ | including obstetric and gynecology patients, the population was heterogeneous. small sample size has limited statistical power. recruitment bias to patients interested in mobile wellness apps. |
| Smith et al. (2021) [89] | Depression | 101 | Not serious | Not applicable | serious | not serious | ⊕⊕⊕◯ |  |
| Smith et al. (2021) [89] | Anxiety | 101 | Not serious | Not applicable | serious | not serious | ⊕⊕⊕◯ |  |
|  |  |  |  |  |  |  |  |  |
| Van Lieshout et al. (2021) [91] | Depression | 403 | Not serious | Not applicable | not serious | not serious | ⊕⊕⊕⊕ | limited genralizability |
| Van Lieshout et al. (2021) [91] | Anxiety | 403 | Not serious | Not applicable | not serious | not serious | ⊕⊕⊕⊕ |  |
|  |  |  |  |  |  |  |  |  |
| Ugarte et al. (2023) [90] | Anxiety | 300 | Not serious | Not applicable | not serious | not serious | ⊕⊕⊕⊕ | self-reported measures. |
|  |  |  |  |  |  |  |  |  |
| Vlassopoulos et al. (2021) [115] | Perceived stress | 2402 | Not serious | Not applicable | not serious | not serious | ⊕⊕⊕⊕ |  |
| Vlassopoulos et al. (2021) [115] | Depression | 2402 | Not serious | Not applicable | not serious | not serious | ⊕⊕⊕⊕ |  |
|  |  |  |  |  |  |  |  |  |
| Wahlund et al. (2020) [116] | Covid-related worry | 670 | not serious | Not applicable | not serious | not serious | ⊕⊕⊕⊕ | self-reported outcomes. Limited generalizability of findings to other contexts |
| Wahlund et al. (2020) [116] | Depression | 670 | Serious | Not applicable | not serious | not serious | ⊕⊕⊕◯ |  |
|  |  |  |  |  |  |  |  |  |
| Wang et al. (2023) [92] | Anxiety | 150 | Not serious | Not applicable | not serious | not serious | ⊕⊕⊕⊕ | small sample size |
| Wang et al. (2023) [92] | Depression | 150 | Serious | Not applicable | not serious | not serious | ⊕⊕⊕◯ |  |
| Wang et al. (2023) [92] | Stress | 150 | Not serious | Not applicable | not serious | not serious | ⊕⊕⊕⊕ |  |
|  |  |  |  |  |  |  |  |  |
| Yook et al. (2024) [93] | Stress | 38 | Serious | Not applicable | not serious | serious | ⊕⊕◯◯ | very small sample size limits the generalizability. study only measured pre-and post-intervention. we cannot identify differences in effectiveness between face-toface and non-face-to-face programs |
| Yook et al. (2024) [93] | Anxiety | 38 | Not serious | Not applicable | not serious | serious | ⊕⊕⊕◯ |  |
|  |  |  |  |  |  |  |  |  |
| Zhang et al. (2023) [94] | Postnatal Depression | 242 | Not serious | Not applicable | not serious | not serious | ⊕⊕⊕⊕ | no blindning of researchers, self-reported measures, only included those with access to internet.. Limiting genralizaibility |
|  |  |  |  |  |  |  |  |  |
| Zhou et al. (2022) [95] | Anxiety | 118 | Not serious | Not applicable | not serious | serious | ⊕⊕⊕◯ | potential Hawthorne effect due to blank control. |
| Zhou et al. (2022) [95] | Depression | 118 | Not serious | Not applicable | not serious | serious | ⊕⊕⊕◯ |  |
|  |  |  |  |  |  |  |  |  |
| Zhang et al. (2025) [105] | Posttraumatic stress | 35 | Not serious | Not applicable | not serious | serious | ⊕⊕⊕◯ | Very small sample size. |
| Zhang et al. (2025) [105] | Postpartum Depression | 35 | Not serious | Not applicable | not serious | serious | ⊕⊕⊕◯ |  |
|  |  |  |  |  |  |  |  |  |
| Wi et al. (2025) [102] | Anxiety | 45 | Serious | Not applicable | serious | serious | ⊕◯◯◯ | Very small sample size. Absence of demographic diversity. The choice of an EOC group may have diluted the observed intervention effects |
| Wi et al. (2025) [102] | Depression | 45 | Serious | Not applicable | serious | serious | ⊕◯◯◯ |  |
|  |  |  |  |  |  |  |  |  |
| Duggal et al. (2025) [101] | Depression | 40 | Serious | Not applicable | not serious | serious | ⊕⊕◯◯ | Very small sample size. several of the outcomes were measured with self-report, which may be subject to social desirability bias. Not powered to assess efficacy |

**Table S4. Results of GRADE per outcome for the non-randomized studies included in the systematic review**

| **Study ID** | **Outcome** | **No. Of participants** | **Risk of Bias** | **Inconsistancy** | **Indirectness** | **Imprecision** | **Certainty (GRADE)** | **Comments** |
| --- | --- | --- | --- | --- | --- | --- | --- | --- |
| Arias et al. (2022) [52] | Postpartum visit attendance rate | 1579 | Serious | Not applicable | Not Serious | Not Serious | ⊕⊕⊕◯ | Pre-post covid implementation comparison. Care likely more then intervention alone. Adjusts for some confounding but can not exclude the pandemic. Imprecision was not considered serious because the study included 1579 participants and reported narrow confidence intervals for the main outcomes. |
|  | Postpartum depression screening | 1579 | Serious | Not applicable | Not Serious | Not Serious | ⊕⊕⊕◯ |  |
|  | Contraception decision made at postpartum visit | 1579 | Serious | Not applicable | Not Serious | Not Serious | ⊕⊕⊕◯ |  |
|  | LARC or permanent sterilization | 1579 | Serious | Not applicable | Not Serious | Not Serious | ⊕⊕⊕◯ |  |
|  | Any breastfeeding at postpartum visit | 1579 | Serious | Not applicable | Not Serious | Not Serious | ⊕⊕⊕◯ |  |
|  | Completion of postpartum glucose tolerance test | 104 | Serious | Not applicable | Not Serious | Not Serious | ⊕⊕⊕◯ |  |
|  | Cardiology follow-up visit attendance rate | 115 | Serious | Not applicable | Not Serious | Not Serious | ⊕⊕⊕◯ |  |
|  |  |  |  |  |  |  |  |  |
| Bantjes et al. (2021) [67] | Anxiety (GAD-7) | 125 | Serious | Not applicable | Not Serious | Not Serious | ⊕⊕⊕◯ | Serious rob but Imprecision was not considered serious because 158 participants initiated treatment and the clinically symptomatic subgroup analyses were based on adequate numbers with large observed effects |
|  | Depression (PHQ-9) | 125 | Serious | Not applicable | Not Serious | Not Serious | ⊕⊕⊕◯ |  |
|  |  |  |  |  |  |  |  |  |
| Bäuerle et al. (2021) [68] | Perceived Stress (PSQ-20) | 110 | Serious | Not applicable | Not Serious | Not Serious | ⊕⊕⊕◯ | Dropout rate of 74.09% - no control group, Imprecision was not considered serious because 110 participants were included in the analysis and statistically significant changes were reported across the main outcomes. |
|  | Depression (PHQ-8) | 110 | Serious | Not applicable | Not Serious | Not Serious | ⊕⊕⊕◯ |  |
|  | Anxiety (GAD-7) | 110 | Serious | Not applicable | Not Serious | Not Serious | ⊕⊕⊕◯ |  |
|  | Self-Efficacy (GSES) | 110 | Serious | Not applicable | Not Serious | Not Serious | ⊕⊕⊕◯ |  |
|  | Mindfulness (FMI) | 110 | Serious | Not applicable | Not Serious | Not Serious | ⊕⊕⊕◯ |  |
|  |  |  |  |  |  |  |  |  |
| Boorboor et al. (2022) [50] | Depression (BDI-II) | 28 | Not Serious | Not applicable | Not Serious | Serious | ⊕⊕⊕◯ | Serious imprecision was judged because the study included only 28 participants in total (14 per group), which limits the precision of the estimated effects. |
|  | Alexithymia (AAS-26) | 28 | Not Serious | Not applicable | Not Serious | Serious | ⊕⊕⊕◯ |  |
|  |  |  |  |  |  |  |  |  |
| Brooks et al. (2024) [69] | Parenting Sense of Competence | 6 | Very Serious | Not applicable | Not Serious | Very Serious | ⊕◯◯◯ | Very serious imprecision was judged because the study included only six participants, and the authors stated that the quantitative results were not statistically meaningful due to the small sample size. |
|  | Depression | 6 | Very Serious | Not applicable | Not Serious | Very Serious | ⊕◯◯◯ |  |
|  | Anxiety | 6 | Very Serious | Not applicable | Not Serious | Very Serious | ⊕◯◯◯ |  |
|  |  |  |  |  |  |  |  |  |
| Cavarretta et al. (2025) [117] | Emotional Exhaustion (EE) | 95 | Serious | Not applicable | Not Serious | Not Serious | ⊕⊕⊕◯ | The certainty of evidence was rated because the main concern was serious risk of bias related to the cross-sectional observational design, while indirectness and imprecision were not considered serious. |
|  | Depersonalization (DP) | 95 | Serious | Not applicable | Not Serious | Not Serious | ⊕⊕⊕◯ |  |
|  | Personal Accomplishment (PA) | 95 | Serious | Not applicable | Not Serious | Not Serious | ⊕⊕⊕◯ |  |
|  |  |  |  |  |  |  |  |  |
| Doornbos et al. (2025) [118] | Depression (PHQ-9) | 47 | Very Serious | Not applicable | Not Serious | Not Serious | ⊕⊕◯◯ | The main concern was very serious risk of bias related to the uncontrolled pre-post design, while indirectness and imprecision were not considered serious. |
|  | Anxiety (GAD-7) | 47 | Very Serious | Not applicable | Not Serious | Not Serious | ⊕⊕◯◯ |  |
|  |  |  |  |  |  |  |  |  |
| Farris et al. (2021) [119] | Momentary Anxiety | 233 | Very Serious | Not applicable | Not Serious | Not Serious | ⊕⊕◯◯ | Very serious risk of bias related to the uncontrolled single-arm design, while indirectness and imprecision were not considered serious. |
|  | Momentary Stress | 233 | Very Serious | Not applicable | Not Serious | Not Serious | ⊕⊕◯◯ |  |
|  | COVID Concerns | 233 | Very Serious | Not applicable | Not Serious | Not Serious | ⊕⊕◯◯ |  |
|  |  |  |  |  |  |  |  |  |
| Gemmill et al. (2022) [70] | Depression | 27 | Very Serious | Not applicable | Not Serious | Serious | ⊕◯◯◯ | Serious imprecision was judged because the study included only 27 women overall, with smaller numbers contributing outcome data at post-treatment and follow-up |
|  | Anxiety | 27 | Very Serious | Not applicable | Not Serious | Serious | ⊕◯◯◯ |  |
|  | Stress | 27 | Very Serious | Not applicable | Not Serious | Serious | ⊕◯◯◯ |  |
|  | PHQ-9 | 27 | Very Serious | Not applicable | Not Serious | Serious | ⊕◯◯◯ |  |
|  |  |  |  |  |  |  |  |  |
| Gonzalez-Garcia et al. (2021) [120] | Stress (PSS-10) | 66 | Very Serious | Not applicable | Not Serious | Not Serious | ⊕⊕◯◯ | Certainty due to very serious risk of bias in an uncontrolled pre-post feasibility study, despite significant improvements in stress, anxiety, and self-compassion. |
|  | Anxiety (STAI-t) | 66 | Very Serious | Not applicable | Not Serious | Not Serious | ⊕⊕◯◯ |  |
|  | Self-compassion (SCS) | 66 | Very Serious | Not applicable | Not Serious | Not Serious | ⊕⊕◯◯ |  |
|  |  |  |  |  |  |  |  |  |
| Hahn et al. (2024) [98] | PPD (EPDS) | 145 | Serious | Not applicable | Not Serious | Not Serious | ⊕⊕⊕◯ | Serious risk of bias due to women with greater symptoms using the app less frequent |
|  | Mood (BSF) | 145 | Serious | Not applicable | Not Serious | Not Serious | ⊕⊕⊕◯ |  |
|  | COVID-19-related-anxieties | 145 | Serious | Not applicable | Not Serious | Not Serious | ⊕⊕⊕◯ |  |
|  |  |  |  |  |  |  |  |  |
| Haro-Ramos et al. (2023) [54] | Depression (PHQ-8) | 398 | Very Serious | Not applicable | Not Serious | Not Serious | ⊕⊕◯◯ | Very serious rob: 65.8% completed pre and post surveys, Self reported outcomes and no control group |
|  | Anxiety (GAD-7) | 398 | Very Serious | Not applicable | Not Serious | Not Serious | ⊕⊕◯◯ |  |
|  |  |  |  |  |  |  |  |  |
| He et al. (2022) [81] | Anxiety (GAD-7) | 62 | Very Serious | Not applicable | Not Serious | Not Serious | ⊕⊕◯◯ | Very serious rob, Imprecision was not considered serious because 62 nurses completed the study and statistically significant differences were reported for the main outcomes across the intervention period. |
|  | Depression (PHQ-9) | 62 | Very Serious | Not applicable | Not Serious | Not Serious | ⊕⊕◯◯ |  |
|  | Self-rating Somatic Symptom Scale (SSS) | 62 | Very Serious | Not applicable | Not Serious | Not Serious | ⊕⊕◯◯ |  |
|  |  |  |  |  |  |  |  |  |
| Iravani et al. (2023) [53] | Health anxiety | 100 | Not Serious | Not applicable | Not Serious | Not Serious | ⊕⊕⊕⊕ | Moderate rob and Imprecision was not considered serious because 100 participants completed the study, evenly distributed across intervention and control groups, and statistically significant between-group differences were reported for both outcomes. |
|  | Pregnant-related anxiety | 100 | Not Serious | Not applicable | Not Serious | Not Serious | ⊕⊕⊕⊕ |  |
|  |  |  |  |  |  |  |  |  |
| Langhammer et al. (2024) [99] | Depression (PHQ-8) | 27 | Serious | Not applicable | Not Serious | Serious | ⊕⊕◯◯ | No control group. Selection Second round of intervention after first round based on symptoms. 1261 baseline and only 142 in main analysis. |
|  | Stress (PSS-10) | 27 | Serious | Not applicable | Not Serious | Serious | ⊕⊕◯◯ |  |
|  | Sleep Quality (PSQI) | 27 | Serious | Not applicable | Not Serious | Serious | ⊕⊕◯◯ |  |
|  |  |  |  |  |  |  |  |  |
| Lestari et al. (2021) [59] | Anxiety (GAD-7) | 46 | Very Serious | Not applicable | Not Serious | Serious | ⊕◯◯◯ | Serious imprecision was judged because the study included only 46 participants, and the effect estimate was based on a small uncontrolled sample. |
|  |  |  |  |  |  |  |  |  |
| Liu et al. (2021) [71] | Anxiety (SAS) | 125 | Not Serious | Not applicable | Not Serious | Not Serious | ⊕⊕⊕⊕ | Moderate rob and Imprecision was not considered serious because 125 participants were enrolled, over 100 completed follow-up, and statistically significant group-by-time effects were reported for the main outcomes. |
|  | Depression (SDS) | 125 | Not Serious | Not applicable | Not Serious | Not Serious | ⊕⊕⊕⊕ |  |
|  | Parenting stress (PSI-SF) | 125 | Not Serious | Not applicable | Not Serious | Not Serious | ⊕⊕⊕⊕ |  |
|  | Hope (HHI) | 125 | Not Serious | Not applicable | Not Serious | Not Serious | ⊕⊕⊕⊕ |  |
|  |  |  |  |  |  |  |  |  |
| Merza et al. (2023) [121] | PPD (EPDS) | 64 | Very Serious | Not applicable | Not Serious | Serious | ⊕◯◯◯ | Very serious rob and Serious imprecision was judged because only 47 participants attended the workshops and provided outcome data, which limits the precision of the estimates in this pilot study |
|  | Aniety (GAD-7) | 64 | Very Serious | Not applicable | Not Serious | Serious | ⊕◯◯◯ |  |
|  | Social provicions | 64 | Very Serious | Not applicable | Not Serious | Serious | ⊕◯◯◯ |  |
|  | Postpartum bonding | 64 | Very Serious | Not applicable | Not Serious | Serious | ⊕◯◯◯ |  |
|  | Infant bonding | 64 | Very Serious | Not applicable | Not Serious | Serious | ⊕◯◯◯ |  |
|  |  |  |  |  |  |  |  |  |
| Ornelas et al. (2022) [72] | Depression | 226 | Serious | Not applicable | Serious | Serious | ⊕◯◯◯ | Outcomes not directely reported so ceirtainty can not be properply estimted for this study |
|  | Anxiety | 226 | Serious | Not applicable | Serious | Serious | ⊕◯◯◯ |  |
|  | Mindfulness | 226 | Serious | Not applicable | Serious | Serious | ⊕◯◯◯ |  |
|  |  |  |  |  |  |  |  |  |
| Valinskas et al. (2023) [73] | Depression (DDAS-21) | 381 | Serious | Not applicable | Not Serious | Not Serious | ⊕⊕⊕◯ | Serious rob but Imprecision was not considered serious because the study included 381 users, repeated symptom assessments were available, and confidence intervals were reported for the main logistic regression findings |
|  | Anxiety (DDAS-21) | 381 | Serious | Not applicable | Not Serious | Not Serious | ⊕⊕⊕◯ |  |
|  | Stress (DDAS-21) | 381 | Serious | Not applicable | Not Serious | Not Serious | ⊕⊕⊕◯ |  |
|  |  |  |  |  |  |  |  |  |
| Van Haeken et al. (2024) [74] | Resiliance (CD-RISC) | 102 | Not Serious | Not applicable | Not Serious | Not Serious | ⊕⊕⊕⊕ | Low rob and Imprecision was not considered serious because the study included both an intervention and a control group, repeated follow-up assessments, and standardized outcome measures, although it remained a pilot study. |
|  | Social Support (MSPSS) | 102 | Not Serious | Not applicable | Not Serious | Not Serious | ⊕⊕⊕⊕ |  |
|  | Self-esteem (RSES) | 102 | Not Serious | Not applicable | Not Serious | Not Serious | ⊕⊕⊕⊕ |  |
|  | Self-efficacy (GSES) | 102 | Not Serious | Not applicable | Not Serious | Not Serious | ⊕⊕⊕⊕ |  |
|  | Sense of mastery (FFMQ) | 102 | Not Serious | Not applicable | Not Serious | Not Serious | ⊕⊕⊕⊕ |  |
|  | Depression (EPDS) | 102 | Not Serious | Not applicable | Not Serious | Not Serious | ⊕⊕⊕⊕ |  |
|  | Anxiety (STAI) | 102 | Not Serious | Not applicable | Not Serious | Not Serious | ⊕⊕⊕⊕ |  |
|  |  |  |  |  |  |  |  |  |
| Young et al. (2025) [122] | Stress | 68 | Not Serious | Not applicable | Not Serious | Not Serious | ⊕⊕⊕⊕ | Low rob and Imprecision was not considered serious because the study included a control group, reported effect sizes, and found significant between-group differences for the main outcomes of total emotional distress and stress, although it remained a pilot study with modest group sizes |
|  | Anxiety | 68 | Not Serious | Not applicable | Not Serious | Not Serious | ⊕⊕⊕⊕ |  |
|  | Depression | 68 | Not Serious | Not applicable | Not Serious | Not Serious | ⊕⊕⊕⊕ |  |

**References**

- Aligoltabar S, Nasiri-Amiri F, Khafri S, et al. Feasibility and acceptability of internet-based emotion-focused cognitive behavioral therapy in women with suspected fetal anomalies: a quasi-experimental study. Jundishapur J Chronic Dis Care. 2025;14(1):e147163. doi:10.5812/jjcdc-147163.
- Boucher VG, Haight BL, Hives BA, et al. Effects of 12 weeks of at-home, application-based exercise on health care workers’ depressive symptoms, burnout, and absenteeism: a randomized clinical trial. JAMA Psychiatry. Nov 1, 2023;80(11):1101-1109. doi:10.1001/jamapsychiatry.2023.2706. Medline:37556150.
- Bryant RA, Dawson KS, Keyan D, et al. Effectiveness of a videoconferencing-delivered psychological intervention for mental health problems during COVID-19: a proof-of-concept randomized clinical trial. Psychother Psychosom. 2022;91(1):63-72. doi:10.1159/000520283. Medline:34875669.
- Chang YC, Chiu CF, Wang CK, Wu CT, Liu LC, Wu YC. Short-term effect of internet-delivered mindfulness-based stress reduction on mental health, self-efficacy, and body image among women with breast cancer during the COVID-19 pandemic. Front Psychol. 2022;13:949446. doi:10.3389/fpsyg.2022.949446. Medline:36389600.
- De Kock JH, Latham HA, Cowden RG, et al. Brief digital interventions to support the psychological well-being of NHS staff during the COVID-19 pandemic: 3-arm pilot randomized controlled trial. JMIR Ment Health. Apr 4, 2022;9(4):e34002. doi:10.2196/34002. Medline:35044927.
- Dominguez-Rodriguez A, Sanz-Gomez S, González Ramírez LP, et al. Evaluation and future challenges in a self-guided web-based intervention with and without chat support for depression and anxiety symptoms during the COVID-19 pandemic: randomized controlled trial. JMIR Form Res. Sep 30, 2024;8:e53767. doi:10.2196/53767. Medline:39348893.
- Dominguez-Rodriguez A, Sanz-Gomez S, González Ramírez LP, et al. The efficacy and usability of an unguided web-based grief intervention for adults who lost a loved one during the COVID-19 pandemic: randomized controlled trial. J Med Internet Res. Apr 6, 2023;25:e43839. doi:10.2196/43839. Medline:36877800.
- Dumarkaite A, Truskauskaite I, Andersson G, et al. The efficacy of the internet-based stress recovery intervention FOREST for nurses amid the COVID-19 pandemic: a randomized controlled trial. Int J Nurs Stud. Feb 2023;138:104408. doi:10.1016/j.ijnurstu.2022.104408. Medline:36527859.
- Ehlis AC, Aas B, Kieckhäfer C, et al. Move, connect and go outside! A randomized controlled trial of two online interventions and analysis of helpful coping strategies in COVID-19 “homestayers”. Appl Psychol Health Well Being. Feb 2025;17(1):e12603. doi:10.1111/aphw.12603. Medline:39414371.
- Fiol-DeRoque MA, Serrano-Ripoll MJ, Jiménez R, et al. A mobile phone-based intervention to reduce mental health problems in health care workers during the COVID-19 pandemic (PsyCovidApp): randomized controlled trial. JMIR Mhealth Uhealth. May 18, 2021;9(5):e27039. doi:10.2196/27039. Medline:33909587.
- Gomà M, Arias-Pujol E, Prims E, et al. Internet-based interdisciplinary therapeutic group (Grupo Interdisciplinar Online, GIO) for perinatal anxiety and depression—a randomized pilot study during COVID-19. Arch Womens Ment Health. Jun 2024;27(3):405-415. doi:10.1007/s00737-023-01412-2. Medline:38150150.
- Guerrero-Pertiñez G, Carbonell-Aranda V, Pérez-Guerrero G, et al. Resilience as a mediator in a web-based intervention (MINDxYOU) to reduce stress among health care professionals: stepped-wedge cluster randomized trial. JMIR Ment Health. Feb 9, 2026;13:e82905. doi:10.2196/82905. Medline:41662682.
- Güney E, Cengizhan SÖ, Karataş Okyay E, Bal Z, Uçar T. Effect of the Mindfulness-Based Stress Reduction program on stress, anxiety, and childbirth fear in pregnant women diagnosed with COVID-19. Complement Ther Clin Pract. May 2022;47:101566. doi:10.1016/j.ctcp.2022.101566. Medline:35219222.
- Jung JH, Ko JY. Depression, anxiety and insomnia among isolated COVID-19 patients: tele occupational therapy intervention vs. conventional one: a comparative study. BMC Psychol. Nov 12, 2024;12(1):649. doi:10.1186/s40359-024-02159-w. Medline:39533442.
- Karing C. The efficacy of online mindfulness-based interventions in a university student sample: videoconference- or podcast-delivered intervention. Appl Psychol Health Well-Being. May 2023;15(2):740-756. doi:10.1111/aphw.12408.
- MacKinnon AL, Simpson KM, Salisbury MR, et al. Building Emotional Awareness and Mental Health (BEAM): a pilot randomized controlled trial of an app-based program for mothers of toddlers. Front Psychiatry. 2022;13:880972. doi:10.3389/fpsyt.2022.880972. Medline:35815022.
- Naja S, Elyamani R, Chehab M, et al. The impact of telemental health interventions on maternal mental health outcomes: a pilot randomized controlled trial during the COVID-19 pandemic. Health Psychol Behav Med. 2023;11(1):1-21. doi:10.1080/21642850.2022.2155167. Medline:36606005.
- Pratt EH, Hall L, Jennings C, et al. Mobile mindfulness for psychological distress and burnout among frontline COVID-19 nurses: a pilot randomized trial. Ann Am Thorac Soc. Oct 2023;20(10):1475-1482. doi:10.1513/AnnalsATS.202301-025OC. Medline:37289650.
- Puertas-Gonzalez JA, Mariño-Narvaez C, Romero-Gonzalez B, Sanchez-Perez GM, Peralta-Ramirez MI. Online cognitive behavioural therapy as a psychological vaccine against stress during the COVID-19 pandemic in pregnant women: a randomised controlled trial. J Psychiatr Res. Aug 2022;152:397-405. doi:10.1016/j.jpsychires.2022.07.016. Medline:35830754.
- Rahgozar S, Giménez-Llort L. Design and effectiveness of an online group logotherapy intervention on the mental health of Iranian international students in European countries during the COVID-19 pandemic. Front Psychiatry. 2024;15:1323774. doi:10.3389/fpsyt.2024.1323774. Medline:38449504.
- Ruzickova T, Carson J, Argabright S, et al. Online behavioural activation during the COVID-19 pandemic decreases depression and negative affective bias. Psychol Med. Feb 2023;53(3):795-804. doi:10.1017/S0033291721002142. Medline:34399873.
- Sockalingam S, Leung SE, Ma C, et al. The impact of telephone-based cognitive behavioral therapy on mental health distress and disordered eating among bariatric surgery patients during COVID-19: preliminary results from a multisite randomized controlled trial. Obes Surg. Jun 2022;32(6):1884-1894. doi:10.1007/s11695-022-05981-6. Medline:35218006.
- Shahamabadi MG, Farajkhoda T, Mahmoodabadi HZ. Online versus face-to-face metacognitive educational counseling program on anxiety and meta-worry in women with a history of miscarriage: a randomized clinical trial. J Educ Health Promot. Jan 1, 2022;11:264. doi:10.4103/jehp.jehp_1292_21. Medline:36325205.
- Smith RB, Mahnert ND, Foote J, Saunders KT, Mourad J, Huberty J. Mindfulness effects in obstetric and gynecology patients during the coronavirus disease 2019 (COVID-19) pandemic: a randomized controlled trial. Obstet Gynecol. Jun 1, 2021;137(6):1032-1040. doi:10.1097/AOG.0000000000004316. Medline:33957663.
- Ugarte DA, Cumberland WG, Singh P, Saadat S, Garett R, Young SD. A HOPE online community peer support intervention for help seeking: a randomized controlled trial. Psychiatr Serv. Jun 1, 2023;74(6):648-651. doi:10.1176/appi.ps.20220168.
- Van Lieshout RJ, Layton H, Savoy CD, et al. Effect of online 1-day cognitive behavioral therapy-based workshops plus usual care vs usual care alone for postpartum depression: a randomized clinical trial. JAMA Psychiatry. Nov 1, 2021;78(11):1200-1207. doi:10.1001/jamapsychiatry.2021.2488. Medline:34495285.
- Vlassopoulos M, Siddique A, Rahman T, Pakrashi D, Islam A, Ahmed F. Improving women’s mental health during a pandemic. Am Econ J Appl Econ. Apr 1, 2024;16(2):422-455. doi:10.1257/app.20210655.
- Wahlund T, Mataix-Cols D, Olofsdotter Lauri K, et al. Brief online cognitive behavioural intervention for dysfunctional worry related to the COVID-19 pandemic: a randomised controlled trial. Psychother Psychosom. 2021;90(3):191-199. doi:10.1159/000512843. Medline:33212440.
- Wang M, Zhang H, Zhang X, et al. Effects of a online brief modified mindfulness-based stress reduction therapy for anxiety among Chinese adults: a randomized clinical trial. J Psychiatr Res. May 2023;161:27-33. doi:10.1016/j.jpsychires.2023.03.009. Medline:36893668.
- Yook YS, Lee JH, Park I, Cho HY. Effects of online mindful somatic psychoeducation program on mental health during the COVID-19. Psychiatry Investig. Jan 2024;21(1):63-73. doi:10.30773/pi.2023.0304. Medline:38198829.
- Zhang Y, Zhu J, Li S, Huang L, Fang Q, Zheng X. The effectiveness of an internet-based support program on maternal self-efficacy, postpartum depression and social support for primiparous women during the COVID-19 pandemic: randomized controlled trial. Front Public Health. 2023;11:1035872. doi:10.3389/fpubh.2023.1035872.
- Zhou K, Kong J, Wan Y, et al. Positive impacts of e-aid cognitive behavioural therapy on the sleep quality and mood of nurses on site during the COVID-19 pandemic. Sleep Breath. Dec 2022;26(4):1947-1951. doi:10.1007/s11325-021-02547-1. Medline:34997900.
- Zhang J, Chen B, Yang X, et al. An internet-based developmental home care support program improved maternal psychological symptoms and infant growth: a randomized controlled trial. J Perinat Neonatal Nurs. 2025;39(1):80-90. doi:10.1097/JPN.0000000000000823. Medline:39325979.
- Wi D, Lee RY, Kantrowitz-Gordon I. Efficacy of online mindfulness for the treatment of insomnia in pregnancy: a randomized clinical trial. PLoS One. 2025;20(5):e0322931. doi:10.1371/journal.pone.0322931. Medline:40343904.
- Duggal M, Sarna A, Dahiya N, et al. A pilot study to evaluate the feasibility and acceptability of a mobile phone counselling intervention for perinatal women living with HIV and depression in India. Oxf Open Digit Health. 2025;3:oqaf012. doi:10.1093/oodh/oqaf012. Medline:40486985.
- Arias MP, Wang E, Leitner K, et al. The impact on postpartum care by telehealth: a retrospective cohort study. Am J Obstet Gynecol MFM. May 2022;4(3):100611. doi:10.1016/j.ajogmf.2022.100611. Medline:35331971.
- Bantjes J, Kazdin AE, Cuijpers P, et al. A web-based group cognitive behavioral therapy intervention for symptoms of anxiety and depression among university students: open-label, pragmatic trial. JMIR Ment Health. May 27, 2021;8(5):e27400. doi:10.2196/27400. Medline:34042598.
- Bäuerle A, Jahre L, Teufel M, et al. Evaluation of the e-mental health mindfulness-based and skills-based “CoPE It” intervention to reduce psychological distress in times of COVID-19: results of a bicentre longitudinal study. Front Psychiatry. 2021;12:768132. doi:10.3389/fpsyt.2021.768132. Medline:34803775.
- Boorboor N, Haddadian Nekah M, Hamidi B, Uosefi M, Eskandarian F, Mousavi S. Online behavioral activation therapy on depression and alexithymia in infertile women during the COVID-19 pandemic lockdown. Arch Hyg Sci. Oct 10, 2022;11(3):210-215. doi:10.34172/AHS.11.3.315.4.
- Brooks KM, Charlot-Swilley D, Robertson HA, et al. “A judgment-free zone”: adaptation and pilot study of a virtual wellness group for African American mothers with young children. Int J Environ Res Public Health. Mar 23, 2024;21(4):390. doi:10.3390/ijerph21040390. Medline:38673302.
- Cavarretta MV, Pellerin H, Maurel E, et al. The effectiveness of online therapy in promoting wellbeing and reducing burnout among psychotherapists. Front Psychol. 2025;16:1510383. doi:10.3389/fpsyg.2025.1510383. Medline:40230990.
- Doornbos MM, Zandee GL, Bajema AG, Luchies LB. Women Supporting Women Zoom: outcomes of online supportive/educative groups for diverse women experiencing depression and anxiety. Arch Psychiatr Nurs. Aug 2025;57:151934. doi:10.1016/j.apnu.2025.151934. Medline:40816797.
- Farris SR, Grazzi L, Holley M, et al. Online mindfulness may target psychological distress and mental health during COVID-19. Glob Adv Health Med. 2021;10:21649561211002461. doi:10.1177/21649561211002461. Medline:34497735.
- Gemmill AW, Oliva JL, Ericksen J, Holt C, Holt CJ, Milgrom J. Web-based treatment for depression in pregnancy: a feasibility study of Mum2BMoodBooster. BMC Psychiatry. Jul 16, 2022;22(1):476. doi:10.1186/s12888-022-04111-x. Medline:35842616.
- González-García M, Álvarez JC, Pérez EZ, Fernandez-Carriba S, López JG. Feasibility of a brief online mindfulness and compassion-based intervention to promote mental health among university students during the COVID-19 pandemic. Mindfulness (N Y). 2021;12(7):1685-1695. doi:10.1007/s12671-021-01632-6. Medline:34025814.
- Hahn L, Thomann M, Dreyer EM, et al. Efficacy of the “7mind”-app as intervention to prevent postpartum depression in a low-risk sample group. Arch Gynecol Obstet. Dec 2024;310(6):3183-3193. doi:10.1007/s00404-024-07851-1. Medline:39630223.
- Haro-Ramos AY, Rodriguez HP, Aguilera A. Effectiveness and implementation of a text messaging intervention to reduce depression and anxiety symptoms among Latinx and Non-Latinx white users during the COVID-19 pandemic. Behav Res Ther. Jun 2023;165:104318. doi:10.1016/j.brat.2023.104318. Medline:37146444.
- He C, Chang S, Lu Y, et al. Effects of online psychological crisis intervention for frontline nurses in COVID-19 pandemic. Front Psychiatry. 2022;13:937573. doi:10.3389/fpsyt.2022.937573. Medline:35903639.
- Iravani M, Bahmaei H, Askari S, Ghanbari S, Nasab MB, Masihi S. Effect of tele-medicine on health anxiety and pregnancy-related anxiety in pregnant women during the COVID-19 epidemic in Iran. Iran J Nurs Midwifery Res. 2023;28(4):405-410. doi:10.4103/ijnmr.ijnmr_405_21. Medline:37694204.
- Langhammer T, Hilbert K, Wasenmueller R, et al. Evaluation of a CBT-based program for mental health in the general population during the COVID-19 pandemic: a stepped-care approach using a chatbot and digitized group intervention. Depress Anxiety. 2024;2024:8950388. doi:10.1155/2024/8950388. Medline:40226668.
- Lestari NE, Anindya I. Does online counseling reduce anxiety in parents of children with autism spectrum disorders during coronavirus disease 2019 pandemic? Open Access Maced J Med Sci. Sep 9, 2021;9(G):66-70. doi:10.3889/oamjms.2021.6492.
- Liu G, Wang S, Liao J, et al. The efficacy of WeChat-based parenting training on the psychological well-being of mothers with children with autism during the COVID-19 pandemic: quasi-experimental study. JMIR Ment Health. Feb 10, 2021;8(2):e23917. doi:10.2196/23917. Medline:33481751.
- Merza D, Amani B, Savoy C, et al. Online peer-delivered group cognitive-behavioral therapy for postpartum depression: a randomized controlled trial. Acta Psychiatr Scand. Nov 2024;150(5):422-432. doi:10.1111/acps.13611. Medline:37649448.
- Ornelas IJ, Perez G, Maurer S, et al. Amigas Latinas Motivando el Alma: in-person and online delivery of an intervention to promote mental health among Latina immigrant women. J Integr Complement Med. Oct 2022;28(10):821-829. doi:10.1089/jicm.2022.0491. Medline:35723668.
- Valinskas S, Nakrys M, Aleknavicius K, Jonusas J. Sensa mobile app for managing stress, anxiety, and depression symptoms: pilot cohort study. JMIR Form Res. Apr 13, 2023;7:e40671. doi:10.2196/40671. Medline:37052990.
- Van Haeken S, Braeken MAKA, Groenen A, Bogaerts A. A supported online resilience-enhancing intervention for pregnant women: a non-randomized pilot study. Int J Environ Res Public Health. Feb 10, 2024;21(2):209. doi:10.3390/ijerph21020209. Medline:38397698.
- Young DKW, Carlbring P, Ng SM, et al. Brief-guided internet-based cognitive behavioural therapy for people with emotional distress during the COVID-19 pandemic. Clin Soc Work J. Jun 2026;54(2):261-273. doi:10.1007/s10615-025-00990-1.
- Sterne JAC, Higgins JPT, Elbers RG, Reeves BC and the development group for ROBINS- I. Risk Of Bias In Non-randomized Studies of Interventions (ROBINS-I): detailed guidance, updated 12 October 2016. Available <http://www.riskofbias.info> [accessed 10/04/2026]

<https://drive.google.com/file/d/0B7IQVI0kum0kenN0SGktSnNHTEE/view?resourcekey=0-ogEhK_bXwu9ZKdrSIdDxNQ>

- Sterne JAC, Savović J, Page MJ, Elbers RG, Blencowe NS, Boutron I, Cates CJ, Cheng H-Y, Corbett MS, Eldridge SM, Hernán MA, Hopewell S, Hróbjartsson A, Junqueira DR, Jüni P,  Kirkham JJ, Lasserson T, Li T, McAleenan A, Reeves BC, Shepperd S, Shrier I, Stewart LA, Tilling K, White IR, Whiting PF, Higgins JPT. RoB 2: a revised tool for assessing risk of bias in randomised trials. *BMJ* 2019; **366**: l4898.
