# Supplementary material for: Effectiveness of Telehealth Care Interventions for Women’s Mental Health During and After the COVID-19 Pandemic: Systematic Review and Meta-Analysis
Source: J Med Internet Res. 2026 Sep 21;28:e85494. doi: 10.2196/85494 (PMC13592667; doi:10.2196/85494)
Supplement: Supplementary file 4 [file jmir-v28-e85494-s004.docx]

**Multimedia Appendix 4. Publication bias, sensitivity analyses, and subgroup analyses**

**Figure S1: Funnel plot used to test publication bias for depression outcomes**

**Figure S2: Sensitivity analysis: overall effect of telehealth intervention on depression outcomes following the exclusion of Zhang et al. (2023) (outlier)**

**Figure S3: Subgroup analysis of depression outcomes based on type of study control (waitlist or standard care)**

**Figure S4: Subgroup analysis of depression outcomes based on % of female participants**

**Figure S5: Subgroup analysis of depression outcomes based on assessment timepoint (⪬1 month, 1-3 months or <3 months post-intervention)**

**Figure S6: Funnel plot used to test publication bias for anxiety outcomes**

**Figure S7: Subgroup analysis of anxiety outcomes based on type of study control (waitlist or standard care)**

**Figure S8: Subgroup analysis of depression outcomes based on % of female participants**

**Figure S9: Subgroup analysis of anxiety outcomes based on assessment timepoint (⪬1 month, 1-3 months or <3 months post-intervention)**

**Figure S10: Funnel plot used to test publication bias for stress outcomes**

**Figure S11: Subgroup analysis of stress outcomes based on % of female participants**

**Figure S12: Subgroup analysis of stress outcomes based on assessment timepoint (⪬1 month or 1-3 months post-intervention)**

**References**

- Wahlund T, Mataix-Cols D, Olofsdotter Lauri K, et al. Brief online cognitive behavioural intervention for dysfunctional worry related to the COVID-19 pandemic: a randomised controlled trial. Psychother Psychosom. 2021;90(3):191-199. doi: 10.1159/000512843. PMID: 33212440.
- Puertas-Gonzalez JA, Mariño-Narvaez C, Romero-Gonzalez B, Sanchez-Perez GM, Peralta-Ramirez MI. Online cognitive behavioural therapy as a psychological vaccine against stress during the COVID-19 pandemic in pregnant women: a randomised controlled trial. J Psychiatr Res. 2022;152:397-405. doi: 10.1016/j.jpsychires.2022.07.016. PMID: 35830754.
- Bryant RA, Dawson KS, Keyan D, et al. Effectiveness of a videoconferencing-delivered psychological intervention for mental health problems during COVID-19: a proof-of-concept randomized clinical trial. Psychother Psychosom. 2022;91(1):63-72. doi: 10.1159/000520283. PMID: 34875669.
- Chang YC, Chiu CF, Wang CK, Wu CT, Liu LC, Wu YC. Short-term effect of internet-delivered mindfulness-based stress reduction on mental health, self-efficacy, and body image among women with breast cancer during the COVID-19 pandemic. Front Psychol. 2022;13:949446. doi: 10.3389/fpsyg.2022.949446. PMID: 36389600.
- De Kock JH, Latham HA, Cowden RG, et al. Brief digital interventions to support the psychological well-being of NHS staff during the COVID-19 pandemic: 3-arm pilot randomized controlled trial. JMIR Ment Health. 2022;9(4):e34002. doi: 10.2196/34002. PMID: 35044927.
- Dominguez-Rodriguez A, Sanz-Gomez S, González Ramírez LP, et al. Evaluation and future challenges in a self-guided web-based intervention with and without chat support for depression and anxiety symptoms during the COVID-19 pandemic: randomized controlled trial. JMIR Form Res. 2024;8:e53767. doi: 10.2196/53767. PMID: 39348893.
- Dominguez-Rodriguez A, Sanz-Gomez S, González Ramírez LP, et al. The efficacy and usability of an unguided web-based grief intervention for adults who lost a loved one during the COVID-19 pandemic: randomized controlled trial. J Med Internet Res. 2023;25:e43839. doi: 10.2196/43839. PMID: 36877800.
- Fiol-DeRoque MA, Serrano-Ripoll MJ, Jiménez R, et al. A mobile phone-based intervention to reduce mental health problems in health care workers during the COVID-19 pandemic (PsyCovidApp): randomized controlled trial. JMIR Mhealth Uhealth. 2021;9(5):e27039. doi: 10.2196/27039. PMID: 33909587.
- Gomà M, Arias-Pujol E, Prims E, et al. Internet-based interdisciplinary therapeutic group (Grupo Interdisciplinar Online, GIO) for perinatal anxiety and depression—a randomized pilot study during COVID-19. Arch Womens Ment Health. 2024;27(3):405-415. doi: 10.1007/s00737-023-01412-2. PMID: 38150150.
- Güney E, Cengizhan SÖ, Karataş Okyay E, Bal Z, Uçar T. Effect of the Mindfulness-Based Stress Reduction program on stress, anxiety, and childbirth fear in pregnant women diagnosed with COVID-19. Complement Ther Clin Pract. 2022;47:101566. doi: 10.1016/j.ctcp.2022.101566. PMID: 35219222.
- Karing C. The efficacy of online mindfulness-based interventions in a university student sample: videoconference- or podcast-delivered intervention. Appl Psychol Health Well-Being. 2023;15(2):740-756. doi: 10.1111/aphw.12408.
- Naja S, Elyamani R, Chehab M, et al. The impact of telemental health interventions on maternal mental health outcomes: a pilot randomized controlled trial during the COVID-19 pandemic. Health Psychol Behav Med. 2023;11(1):1-21. doi: 10.1080/21642850.2022.2155167. PMID: 36606005.
- Pratt EH, Hall L, Jennings C, et al. Mobile mindfulness for psychological distress and burnout among frontline COVID-19 nurses: a pilot randomized trial. Ann Am Thorac Soc. 2023;20(10):1475-1482. doi: 10.1513/AnnalsATS.202301-025OC. PMID: 37289650.
- Sockalingam S, Leung SE, Ma C, et al. The impact of telephone-based cognitive behavioral therapy on mental health distress and disordered eating among bariatric surgery patients during COVID-19: preliminary results from a multisite randomized controlled trial. Obes Surg. 2022;32(6):1884-1894. doi: 10.1007/s11695-022-05981-6. PMID: 35218006.
- Shahamabadi MG, Farajkhoda T, Mahmoodabadi HZ. Online versus face-to-face metacognitive educational counseling program on anxiety and meta-worry in women with a history of miscarriage: a randomized clinical trial. J Educ Health Promot. 2022;11:264. doi: 10.4103/jehp.jehp_1292_21. PMID: 36325205.
- Smith RB, Mahnert ND, Foote J, Saunders KT, Mourad J, Huberty J. Mindfulness effects in obstetric and gynecology patients during the coronavirus disease 2019 (COVID-19) pandemic: a randomized controlled trial. Obstet Gynecol. 2021;137(6):1032-1040. doi: 10.1097/AOG.0000000000004316. PMID: 33957663.
- Van Lieshout RJ, Layton H, Savoy CD, et al. Effect of online 1-day cognitive behavioral therapy-based workshops plus usual care vs usual care alone for postpartum depression: a randomized clinical trial. JAMA Psychiatry. 2021;78(11):1200-1207. doi: 10.1001/jamapsychiatry.2021.2488. PMID: 34495285.
- Yook YS, Lee JH, Park I, Cho HY. Effects of online mindful somatic psychoeducation program on mental health during the COVID-19. Psychiatry Investig. 2024;21(1):63-73. doi: 10.30773/pi.2023.0304. PMID: 38198829.
- Zhang Y, Zhu J, Li S, Huang L, Fang Q, Zheng X. The effectiveness of an internet-based support program on maternal self-efficacy, postpartum depression and social support for primiparous women during the COVID-19 pandemic: randomized controlled trial. Front Public Health. 2023;11:1035872. doi: 10.3389/fpubh.2023.1035872.
- Zhou K, Kong J, Wan Y, et al. Positive impacts of e-aid cognitive behavioural therapy on the sleep quality and mood of nurses on site during the COVID-19 pandemic. Sleep Breath. 2022;26(4):1947-1951. doi: 10.1007/s11325-021-02547-1. PMID: 34997900.
- Zhang J, Chen B, Yang X, et al. An internet-based developmental home care support program improved maternal psychological symptoms and infant growth: a randomized controlled trial. J Perinat Neonatal Nurs. 2025;39(1):80-90. doi: 10.1097/JPN.0000000000000823. PMID: 39325979.
- Wi D, Lee RY, Kantrowitz-Gordon I. Efficacy of online mindfulness for the treatment of insomnia in pregnancy: a randomized clinical trial. PLoS One. 2025;20(5):e0322931. doi: 10.1371/journal.pone.0322931. PMID: 40343904.
- Duggal M, Sarna A, Dahiya N, et al. A pilot study to evaluate the feasibility and acceptability of a mobile phone counselling intervention for perinatal women living with HIV and depression in India. Oxf Open Digit Health. 2025;3:oqaf012. doi: 10.1093/oodh/oqaf012. PMID: 40486985.
