# Supplementary material for: Effectiveness of Telehealth Care Interventions for Women’s Mental Health During and After the COVID-19 Pandemic: Systematic Review and Meta-Analysis
Source: J Med Internet Res. 2026 Sep 21;28:e85494. doi: 10.2196/85494 (PMC13592667; doi:10.2196/85494)
Supplement: Supplementary file 5 [file jmir-v28-e85494-s005.docx]

| **Section and Topic** | **Item #** | **Checklist item** | **Location where item is reported** |
| --- | --- | --- | --- |
| **TITLE** | | |  |
| Title | 1 | Identify the report as a systematic review. | Title (page 1) |
| **ABSTRACT** | | |  |
| Abstract | 2 | See the PRISMA 2020 for Abstracts checklist. | Page 2 |
| **INTRODUCTION** | | |  |
| Rationale | 3 | Describe the rationale for the review in the context of existing knowledge. | Background section of Introduction (page 4) |
| Objectives | 4 | Provide an explicit statement of the objective(s) or question(s) the review addresses. | Study Objectives section of Introduction (page 4,5) |
| **METHODS** | | |  |
| Eligibility criteria | 5 | Specify the inclusion and exclusion criteria for the review and how studies were grouped for the syntheses. | Inclusion and exclusion criteria section of the Methods (page 6,7,8) |
| Information sources | 6 | Specify all databases, registers, websites, organisations, reference lists and other sources searched or consulted to identify studies. Specify the date when each source was last searched or consulted. | Search strategy section of the Methods (page 6) |
| Search strategy | 7 | Present the full search strategies for all databases, registers and websites, including any filters and limits used. | Detailed data extraction strategy in Multimedia Appendix 1 |
| Selection process | 8 | Specify the methods used to decide whether a study met the inclusion criteria of the review, including how many reviewers screened each record and each report retrieved, whether they worked independently, and if applicable, details of automation tools used in the process. | Data extraction section of the Methods (page 8) |
| Data collection process | 9 | Specify the methods used to collect data from reports, including how many reviewers collected data from each report, whether they worked independently, any processes for obtaining or confirming data from study investigators, and if applicable, details of automation tools used in the process. | Data extraction section of the Methods (page 8) |
| Data items | 10a | List and define all outcomes for which data were sought. Specify whether all results that were compatible with each outcome domain in each study were sought (e.g. for all measures, time points, analyses), and if not, the methods used to decide which results to collect. | Outcomes Measurement Tool section of the Methods (page 8) |
|  | 10b | List and define all other variables for which data were sought (e.g. participant and intervention characteristics, funding sources). Describe any assumptions made about any missing or unclear information. | Outcomes Measurement Tool section of the Methods (page 8), Table 1.1 (page 14), and table 1 (Multimedia Appendix 2) |
| Study risk of bias assessment | 11 | Specify the methods used to assess risk of bias in the included studies, including details of the tool(s) used, how many reviewers assessed each study and whether they worked independently, and if applicable, details of automation tools used in the process. | Risk of Bias Assessment section in Methods (page 8,9) |
| Effect measures | 12 | Specify for each outcome the effect measure(s) (e.g. risk ratio, mean difference) used in the synthesis or presentation of results. | Statistical Data Analysis section in Methods (page 9) |
| Synthesis methods | 13a | Describe the processes used to decide which studies were eligible for each synthesis (e.g. tabulating the study intervention characteristics and comparing against the planned groups for each synthesis (item #5)). | Statistical Data Analysis section in Methods (page 9) |
|  | 13b | Describe any methods required to prepare the data for presentation or synthesis, such as handling of missing summary statistics, or data conversions. | Statistical Data Analysis section in Methods (page 9) |
|  | 13c | Describe any methods used to tabulate or visually display results of individual studies and syntheses. | Statistical Data Analysis section in Methods (page 9) |
|  | 13d | Describe any methods used to synthesize results and provide a rationale for the choice(s). If meta-analysis was performed, describe the model(s), method(s) to identify the presence and extent of statistical heterogeneity, and software package(s) used. | Statistical Data Analysis section in Methods (page 9) |
|  | 13e | Describe any methods used to explore possible causes of heterogeneity among study results (e.g. subgroup analysis, meta-regression). | Statistical Data Analysis section in Methods (page 9) |
|  | 13f | Describe any sensitivity analyses conducted to assess robustness of the synthesized results. | Statistical Data Analysis section in Methods (page 9) |
| Reporting bias assessment | 14 | Describe any methods used to assess risk of bias due to missing results in a synthesis (arising from reporting biases). | Risk of Bias Assessment section in Methods (page 8,9) |
| Certainty assessment | 15 | Describe any methods used to assess certainty (or confidence) in the body of evidence for an outcome. | Certainty of Evidence assessment (GRADE) section in Methods  (page 9,10) |
| **RESULTS** | | |  |
| Study selection | 16a | Describe the results of the search and selection process, from the number of records identified in the search to the number of studies included in the review, ideally using a flow diagram. | Study Selection section of the Results (page 11) and Figure 1 |
|  | 16b | Cite studies that might appear to meet the inclusion criteria, but which were excluded, and explain why they were excluded. | Figure 1 |
| Study characteristics | 17 | Cite each included study and present its characteristics. | General Studies Characteristics section in Results (page 13), Table 1.1, Multimedia Appendix 2 |
| Risk of bias in studies | 18 | Present assessments of risk of bias for each included study. | Risk of Bias Assessment section in Results (Page 17) and Tables 2,3 |
| Results of individual studies | 19 | For all outcomes, present, for each study: (a) summary statistics for each group (where appropriate) and (b) an effect estimate and its precision (e.g. confidence/credible interval), ideally using structured tables or plots. | (Page 11-24) And Multimedia Appendix 2,3,4 |
| Results of syntheses | 20a | For each synthesis, briefly summarise the characteristics and risk of bias among contributing studies. | General Studies Characteristics section in Results (page 13), Risk of Bias Assessment section in Results (Page 17) |
|  | 20b | Present results of all statistical syntheses conducted. If meta-analysis was done, present for each the summary estimate and its precision (e.g. confidence/credible interval) and measures of statistical heterogeneity. If comparing groups, describe the direction of the effect. | Meta-analysis Findings section of the Results (pages 20-23) and Figures 2-4 |
|  | 20c | Present results of all investigations of possible causes of heterogeneity among study results. | Meta-analysis Findings section of the Results (pages 20-23), Figures 2-4, and Multimedia Appendix 3 |
|  | 20d | Present results of all sensitivity analyses conducted to assess the robustness of the synthesized results. | Meta-analysis Findings section of the Results (pages 20-23), Figures 2-4, and Multimedia Appendix 3 |
| Reporting biases | 21 | Present assessments of risk of bias due to missing results (arising from reporting biases) for each synthesis assessed. | Meta-analysis Findings section of the Results (pages 20-23) and Tables 2-3 |
| Certainty of evidence | 22 | Present assessments of certainty (or confidence) in the body of evidence for each outcome assessed. | Quality of Evidence section in Results (page 24) and Multimedia Appendix 4 |
| **DISCUSSION** | | |  |
| Discussion | 23a | Provide a general interpretation of the results in the context of other evidence. | Main Findings and Comparison with Prior Work section in the Discussion (pages 25) |
|  | 23b | Discuss any limitations of the evidence included in the review. | Limitations section of the Discussion (pages 27) |
|  | 23c | Discuss any limitations of the review processes used. | Limitations section of the Discussion (pages 27) |
|  | 23d | Discuss implications of the results for practice, policy, and future research. | Further Work and Implications for policy and practice sections of the Discussion (page 26, 28) |
| **OTHER INFORMATION** | | |  |
| Registration and protocol | 24a | Provide registration information for the review, including register name and registration number, or state that the review was not registered. | Abstract (page 2,3) |
|  | 24b | Indicate where the review protocol can be accessed, or state that a protocol was not prepared. | Abstract (page 2,3) and Ethical Considerations (page 6) |
|  | 24c | Describe and explain any amendments to information provided at registration or in the protocol. | Ethical Considerations (page 6) |
| Support | 25 | Describe sources of financial or non-financial support for the review, and the role of the funders or sponsors in the review. | Funding declaration (page 30) |
| Competing interests | 26 | Declare any competing interests of review authors. | Declaration of interests (page 30) |
| Availability of data, code and other materials | 27 | Report which of the following are publicly available and where they can be found: template data collection forms; data extracted from included studies; data used for all analyses; analytic code; any other materials used in the review. | Data Availability (page 30) |

*From:*  Page MJ, McKenzie JE, Bossuyt PM, Boutron I, Hoffmann TC, Mulrow CD, et al. The PRISMA 2020 statement: an updated guideline for reporting systematic reviews. BMJ 2021;372:n71. doi: 10.1136/bmj.n71. This work is licensed under CC BY 4.0. To view a copy of this license, visit <https://creativecommons.org/licenses/by/4.0/>

**PRISMA 2020 for Abstracts Checklist**

| **Section and Topic** | **Item**  **#** | **Checklist item** | **Reported**  **(Yes/No)** |
| --- | --- | --- | --- |
| **TITLE** | | |  |
| Title | 1 | Identify the report as a systematic review. | Yes, Title |
| **BACKGROUND** | | |  |
| Objectives | 2 | Provide an explicit statement of the main objective(s) or question(s) the review addresses. | Yes, Objective |
| **METHODS** | | |  |
| Eligibility criteria | 3 | Specify the inclusion and exclusion criteria for the review. | Yes, Methods |
| Information sources | 4 | Specify the information sources (e.g. databases, registers) used to identify studies and the date when each was last searched. | Yes, Methods |
| Risk of bias | 5 | Specify the methods used to assess risk of bias in the included studies. | Yes, Methods |
| Synthesis of results | 6 | Specify the methods used to present and synthesise results. | Yes, Methods |
| **RESULTS** | | |  |
| Included studies | 7 | Give the total number of included studies and participants and summarise relevant characteristics of studies. | Yes, Results |
| Synthesis of results | 8 | Present results for main outcomes, preferably indicating the number of included studies and participants for each. If meta-analysis was done, report the summary estimate and confidence/credible interval. If comparing groups, indicate the direction of the effect (i.e. which group is favoured). | Yes, Results |
| **DISCUSSION** | | |  |
| Limitations of evidence | 9 | Provide a brief summary of the limitations of the evidence included in the review (e.g. study risk of bias, inconsistency and imprecision). | Yes, Results |
| Interpretation | 10 | Provide a general interpretation of the results and important implications. | Yes, Conclusion |
| **OTHER** | | |  |
| Funding | 11 | Specify the primary source of funding for the review. | Yes, Listed under funding section |
| Registration | 12 | Provide the register name and registration number. | Yes, PROSPERO registration |

*From:* Page MJ, McKenzie JE, Bossuyt PM, Boutron I, Hoffmann TC, Mulrow CD, et al. The PRISMA 2020 statement: an updated guideline for reporting systematic reviews. BMJ 2021;372:n71. doi: 10.1136/bmj.n71. This work is licensed under CC BY 4.0. To view a copy of this license, visit <https://creativecommons.org/licenses/by/4.0/>
